# Metabolomic response to collegiate football participation: *Pre*- and *Post*-season analysis

**DOI:** 10.1038/s41598-022-07079-6

**Published:** 2022-02-23

**Authors:** Nicole L. Vike, Sumra Bari, Khrystyna Stetsiv, Thomas M. Talavage, Eric A. Nauman, Linda Papa, Semyon Slobounov, Hans C. Breiter, Marilyn C. Cornelis

**Affiliations:** 1 Warren Wright Adolescent Center Department of Psychiatry and Behavioral Sciences, Feinberg School of Medicine, Northwestern University, Chicago, IL USA; 2 Department of Biomedical Engineering, University of Cincinnati, Cincinnati, OH USA; 3 Weldon School of Biomedical Engineering, Purdue University, West Lafayette, IN USA; 4 School of Mechanical Engineering, Purdue University, West Lafayette, IN USA; 5 Department of Basic Medical Sciences, Purdue University, West Lafayette, IN USA; 6 Department of Emergency Medicine, Orlando Regional Medical Center, Orlando, FL USA; 7 Department of Kinesiology, Pennsylvania State University, University Park, PA USA; 8 Laboratory of Neuroimaging and Genetics, Department of Psychiatry, Massachusetts General Hospital and Harvard School of Medicine, Boston, MA USA; 9 Department of Preventive Medicine, Northwestern University Feinberg School of Medicine, Chicago, IL USA

**Keywords:** Transcriptomics, Biomarkers, Brain injuries

## Abstract

Contact sports participation has been shown to have both beneficial and detrimental effects on health, however little is known about the metabolic sequelae of these effects. We aimed to identify metabolite alterations across a collegiate American football season. Serum was collected from 23 male collegiate football athletes before the athletic season (*Pre*) and after the last game (*Post*). Samples underwent nontargeted metabolomic profiling and 1131 metabolites were included for univariate, pathway enrichment, and multivariate analyses. Significant metabolites were assessed against head acceleration events (HAEs). 200 metabolites changed from *Pre* to *Post* (P < 0.05 and Q < 0.05); 160 had known identity and mapped to one of 57 pre-defined biological pathways. There was significant enrichment of metabolites belonging to five pathways (P < 0.05): xanthine, fatty acid (acyl choline), medium chain fatty acid, primary bile acid, and glycolysis, gluconeogenesis, and pyruvate metabolism. A set of 12 metabolites was sufficient to discriminate *Pre* from *Post* status, and changes in 64 of the 200 metabolites were also associated with HAEs (P < 0.05). In summary, the identified metabolites, and candidate pathways, argue there are metabolic consequences of both physical training and head impacts with football participation. These findings additionally identify a potential set of objective biomarkers of repetitive head injury.

## Introduction

Recent research has pointed to the potentially deleterious consequences of participating in contact sports, such as American football and soccer^1–3^. These athletes experience impacts to both body and head during play and brain alterations related to head acceleration events (HAEs) have been documented^3–6^. Despite these consequences, athletic participation and training has many benefits, including improved cardiovascular endurance and psychosocial development^7^. Given this dichotomy, it is imperative to understand the balance between positive and negative health consequences of contact sport participation.

High-throughput metabolite profiling techniques enable comprehensive studies of an individual’s metabolic response to certain conditions^8,9^. Here, metabolite profiling could provide new mechanistic insight into the dichotomy between exercise benefits and HAE-related consequences. Further, knowledge of an objective ‘metabolite signature’ reflecting potential changes from baseline may also be used to optimize clinical and epidemiological tools for physical assessment in this population of athletes. Previous studies have observed peripheral metabolite changes in contact athletes, but the relationship of these changes to HAEs has not yet been investigated^8–10^. Additionally, magnetic resonance spectroscopy, a noninvasive neuroimaging technique, has revealed localized neurometabolic changes in contact sport athletes^11,12^.

In the current study, we assessed the metabolomics of collegiate American football athletes before seasonal play (*Pre*) and within one week after the last game of the season (*Post*). Our primary objective was to identify individual metabolite changes in response to athletic participation to gain insight into biological mechanisms by which football participation may impact performance, both positively and negatively. Our secondary objective was to identify metabolite signatures that discriminate periods of competitive play from periods of off-season rest which thus have potential utility in clinical or epidemiological studies of football and health. Finally, we assessed associations *post-hoc* between HAEs and metabolite change between *Pre* and *Post* to provide insight into metabolic change that may be, in part, due to head impacts. Together, these findings may elucidate a set of objective biomarkers that could be used to flag signs of head injury that are not easily detected with traditional behavioral assessments^13,14^.

## Methods

### Participants and sample collection

Twenty-three male collegiate American football athletes were recruited for this study as reported previously^15^. These athletes were well-seasoned starters, 16 were non-speed linemen, who experience a high number of HAEs^16,17^. The study was approved by the Pennsylvania State University Institutional Review Board in accordance with the Declaration of Helsinki and written informed consent was obtained from each subject. Demographic information was obtained from each athlete and confirmed by a team physician: age (mean = 21 ± 1 year), race (12 white, 11 African American), years of play experience (YoE; mean = 11 ± 3 years), player position (16 non-speed linemen, 7 speed), and history of diagnosed concussion (HoC; 9 with positive history)^15^. None of the athletes received a concussion diagnosis in the 9 months preceding preseason data collection. Five mL of venous blood was collected from each athlete before contact practices (*Pre*) and within one week following the last regular season game (*Post*). Athletes were not required to fast prior to blood collection given they were enrolled in strict nutritional programs. Samples were placed in a serum separator tube, allowed to clot at room temperature, and then centrifuged. Serum was extracted from each tube and pipetted into bar-coded aliquot tubes. Serum samples were stored at – 70 °C until they were transported to Metabolon Inc. (Durham, NC, USA) for blinded metabolite analysis. HAEs were collected at each practice session (i.e., between *Pre* and *Post* blood sampling) using BodiTrak’s Head Health Network sensor system. For each athlete, HAEs were quantified as the cumulative number of hits exceeding the threshold Th = 25G and 80G (e.g., cHAE25G and cHAE80G for each athlete) as well as the average number of hits exceeding 25G and 80G per practice session with recorded impacts (aHAE25G and aHAE80G)^12^. The HAE monitoring protocol and index derivation are detailed in Supplemental Digital Content. Because the majority of head impacts occur during practices and not games^18^, and to avoid disrupting game preparation, HAEs were monitored at practice sessions only*.*

### Metabolomics assay, data acquisition and processing

Serum samples were subject to nontargeted metabolomic profiling using UPLC-ESI–MS/MS as previously described^19,20^ and detailed in the Supplemental Digital Content. Mass spectral peaks, retention times, and m/z were used to determine the relative quantities of each metabolite. Missing values were imputed with the observed minimum value following normalization and scaling steps. Individual metabolites that contained more than 50% missing values in both *Pre* and *Post* samples were not included for statistical analysis (64 metabolites). The final 1131 metabolites analyzed in the current study are listed in Supplemental Table S1; of those, 209 have not been identified with a known chemical structure and are indicated with prefix “X-” followed by a number (e.g., X-23665).

### Statistical analysis

Statistical analyses were performed using R, SAS version 9.2 (SAS Institute Inc, Cary, NC, USA), MetaboAnalyst, or Matlab using log-transformed metabolite values. We first performed standard principal component analysis (PCA) and multilevel PCA to explore the data and identify any outlier samples^21^. For the latter, we generated a data matrix of the within-person variation by subtracting individual metabolite values from the mean metabolite value of *Pre* and *Post*, per participant, per metabolite.

#### Univariate analysis: individual metabolite changes in response to football participation

Paired t-tests were used to identify metabolites that differed significantly between *Pre* and *Post*. Statistical significance was defined as P < 0.05 and FDR (Q value) < 0.05. Pathway enrichment analysis was performed using MetaboLync (Metabolon Inc., Durham, NC, USA), with all metabolites and their pre-assigned pathways as background and reference pathways, respectively. Analyses were restricted to the 67 pathways containing at least 5 metabolite members. Correction for multiple hypothesis testing in pathway enrichment analysis was performed using an FDR of 5%. We computed both Pearson correlations and pairwise partial correlations to explore the latent relationships of changes in identified metabolites ($\Delta$metabolite, see Supplemental Digital Content). Correlation networks were constructed using Cytoscape^22^.

#### Multivariate analysis: sample classification and predictive metabolite-screening

Multilevel partial least squares discriminant analysis (mPLSDA)^23^ was performed to examine whether systemic metabolic changes occurred over a single season of football and which metabolites were the most differentiating biomarkers. The prediction error of the mPLSDA model was determined and expressed in terms of number of misclassifications (NMC) and Q^2^ by a fivefold cross model validation (CMV) scheme^24^. To obtain stable class predictions, and stable metabolite selections, the average result of 20 CMVs was calculated. To validate whether the prediction error of the mPLSDA model was not obtained by chance, a comparison was made with the prediction errors from 1000 randomly permutated data sets representing the H_0_-distribution of no-effect. The football season effect was considered statistically significant if the P value obtained from this permutation test was < 0.05. To select the most discriminative metabolites between *Pre* and *Post*, the metabolites were ranked according to their absolute size in the mPLSDA regression coefficient based on the average result of 20 CMVs. Metabolites with the lowest rank product (RP) values have the strongest discriminative power. The RP values of the mPLSDA model were compared with the RP values obtained from 1000 permutations and those with a P value < 0.05 were considered significantly discriminative. Random Forest (RF) analysis, a non-parametric technique unaffected by feature scale, was also implemented as a secondary multivariate analysis (Supplemental Digital Content).

#### Hierarchical clustering analysis (HCA)

Multilevel HCA was used to further visualize pathway enrichment and the discriminatory ability of the significant metabolites identified by univariate and multivariate analysis. HCA was performed using Euclidean distance and complete linkage for grouping of clusters (samples only).

## Results

PCA demonstrated separation of *Pre* and *Post* samples along the first component (Supplemental Fig S1). The first component accounted for 9.6% (standard PCA, Supplemental Fig S1a) of the total variance of the data while the multilevel PCA (Supplemental Fig S1b) was able to describe 19.9% of the *within-person* variation. Outliers were vetted for potential technical errors but provided no reason to exclude any samples from our primary analysis.

### Individual metabolite changes in response to football participation

**Figure 1:**
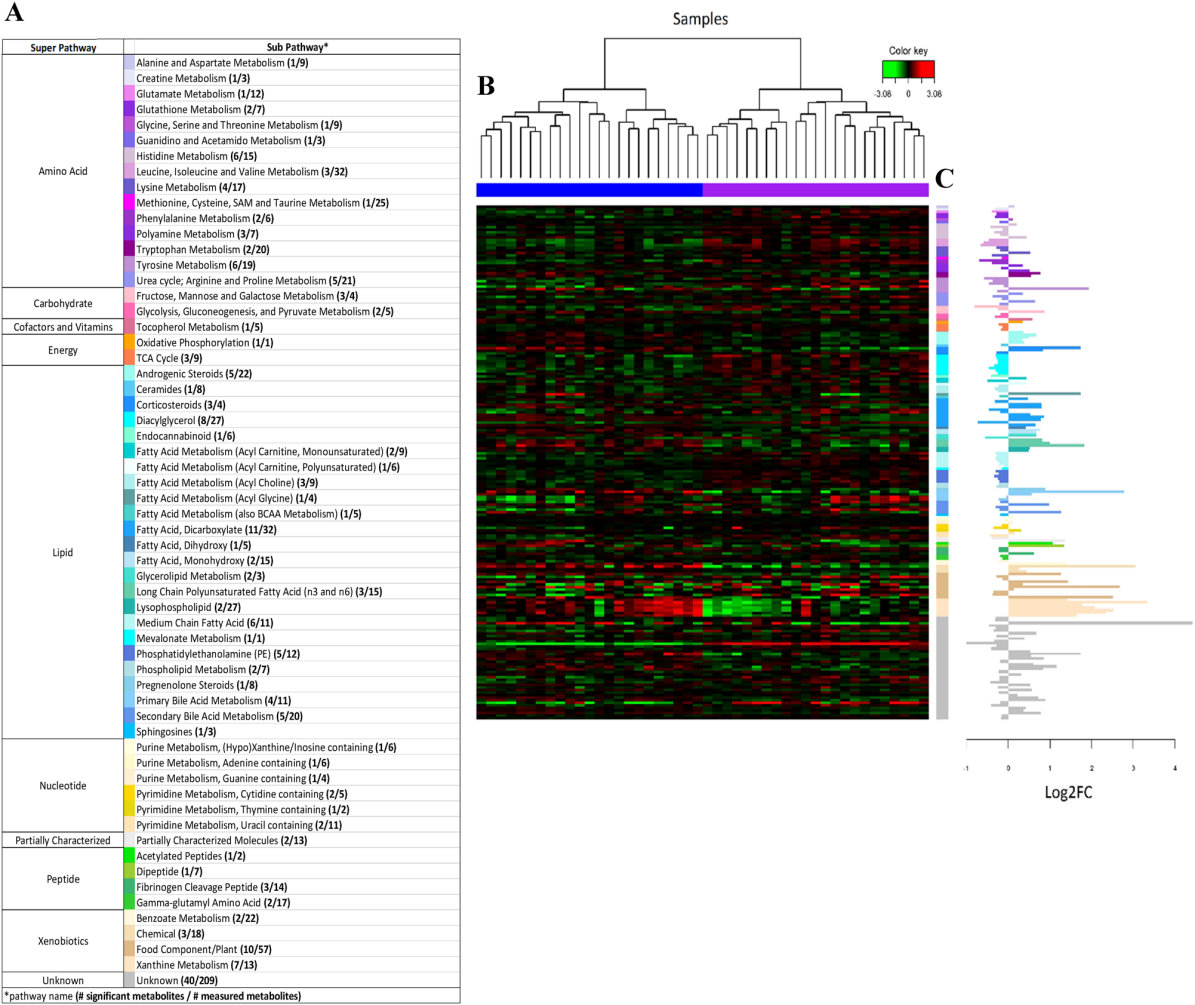
(**A**) Summary of pathways with metabolites significantly (P < 0.05, Q < 0.05) changed after a season of football. (**B**) Multilevel hierarchical clustering (samples only) of the 200 identified metabolites. Green and red cells correspond to low and high metabolite levels, respectively. Columns are samples and rows are metabolites organized by subpathway (see **A** for color key). (**C**) Log2 fold change (FC) for each significant metabolite organized by subpathway (see (**A**) for color key and Supplemental Table S2 for details).

A total of 200 metabolites significantly changed over the course of the football season and these mapped to 57 subpathways (Fig. 1, Supplemental Table S2). Of these metabolites, 90 and 110 demonstrated increases and decreases, respectively. Forty metabolites were of unknown identity.

The metabolomics dataset was significantly enriched for metabolite members of xanthine metabolism (P = 2.2 × 10^–05^, 3.2-fold-enrichment), fatty acid metabolism (acyl choline, P = 0.002, 2.9-fold enrichment), medium chain fatty acid (MCFA, P = 0.01, 2.4-fold-enrichment), primary bile acid metabolism (P = 0.01, 2.4-fold enrichment) and glycolysis, gluconeogenesis, and pyruvate metabolism (P = 0.02, 3.0-fold enrichment) pathways. Metabolite members of these pathways that achieved only nominal significance (i.e., P < 0.05 but Q > 0.05) are also listed in Supplemental Table S2.

**Figure 2:**
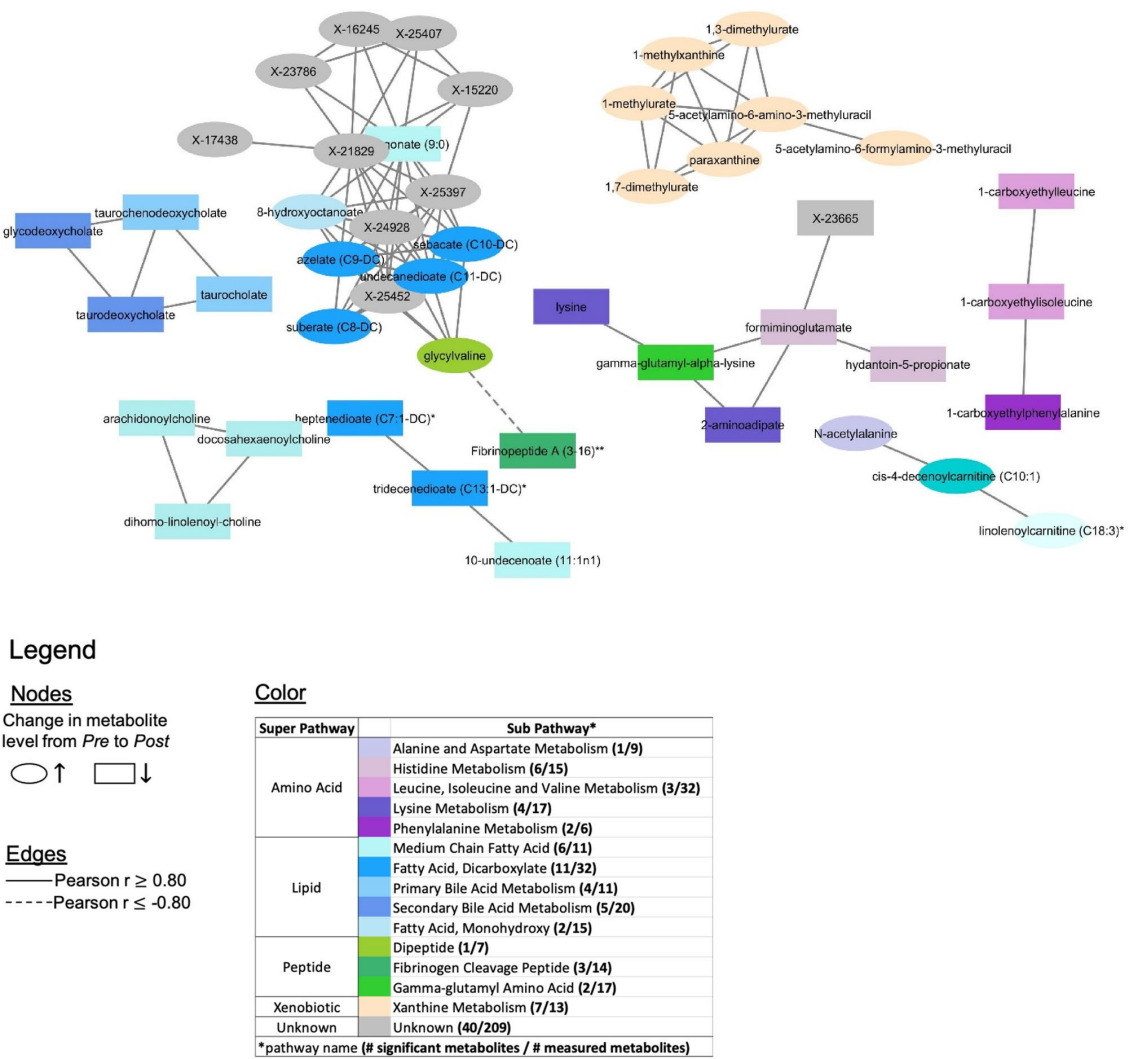
Pearson correlations (r) of changes in metabolite levels after a season of football. Edges correspond to r and are shown if |r| > 0.80. Distances between nodes have no meaning. Only networks of at least 3 members are shown. See Fig. 1A for color key and Supplemental Fig S2 for all networks.

Focusing only on the 200 significant metabolites, with the exception of three metabolites (1-methyl-4-imidazoleacetate, fructose, X-21467), change in each metabolite was significantly correlated with change in at least one other metabolite based on Pearson |r| = 0.50; forming a single network of 197 nodes (metabolites) and 1062 edges (correlations). Applying the more stringent threshold |r| = 0.80 resulted in 7 networks of 3 or more nodes that were largely consistent with pathway membership (Fig. 2). Nine metabolites of unknown identity formed a network with pelargonate, 8-hydroxyoctanoate, glycylvaline, and 4 dicarboxylate fatty acids. Supplemental Figure S2 presents the *partial* correlation networks for changes in metabolite levels which, as expected, are much weaker (max |r_par_| = 0.10) and sparser (77 edges) then their corresponding ordinary correlation networks, because they aim to capture only direct connections between metabolites.

### *Pre* and *Post* classification and predictive metabolite-screening

**Figure 3:**
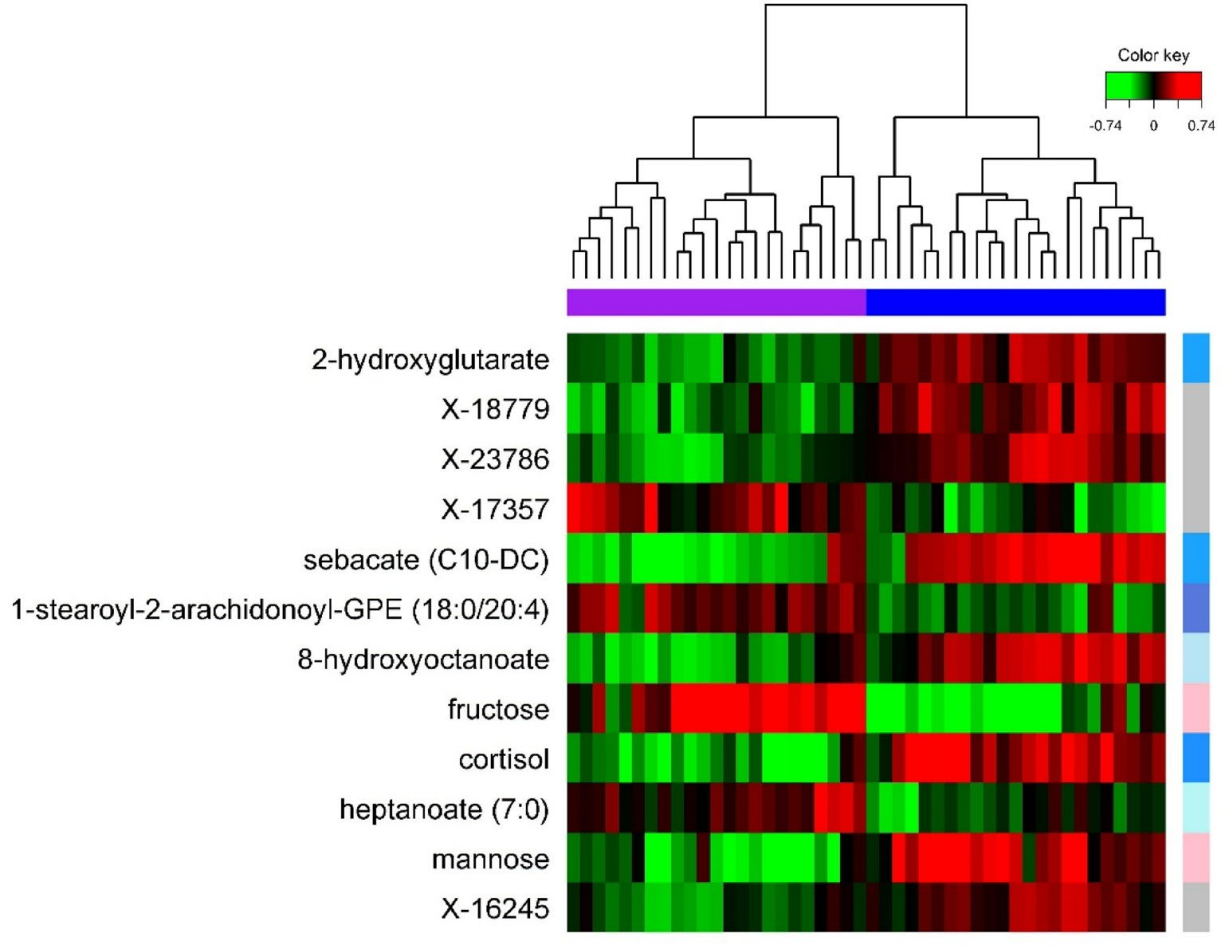
Multilevel hierarchical clustering (samples only) based on the 12 metabolites identified by mPLSDA. Green and red cells correspond to low and high metabolite levels, respectively. Columns are participant samples (i.e., two samples per athlete) and rows are metabolites colored and organized by subpathway (see Fig. 1A for color key).

Supplemental Figure S3 presents the results of mPLSDA. On average 2 out of 46 samples (4%) were misclassified (P < 0.05 classification model). Significant season effects were also observed based on the Q^2^ classification criteria (data not shown). The mPLSDA model yielded 12 metabolites that significantly (P < 0.05) discriminated between *Pre* and *Post* (Fig. 3, Supplemental Table S3); all were significant in the univariate analysis. Secondary multilevel RF analysis identified a set of 9 metabolites that yielded optimal classification performance (Supplemental Fig. S4); 5 of these overlapped with the 12 identified by mPLSDA.

### Hierarchical clustering analysis

Multilevel HCA based on (1) all 200 identified metabolites (Fig. 1B) or (2) the subset of metabolites showing greatest discriminative performance based on mPLSDA (Fig. 3) demonstrate excellent sample clustering performance.

### Post-hoc metabolite and HAE regression analysis

Regression analyses conducted between all 200 identified metabolites and five HAE metrics (number of practice sessions with recorded impacts, cHAE_25G,_ cHAE_80G_, aHAE_25G_ and aHAE_80G_) revealed 64 significant (P < 0.05) regressions between five HAE metrics and 49 metabolites (Supplemental Table S4). The largest class of metabolites involved in these relationships were lipids, followed by unidentified metabolites, amino acids, xenobiotics, carbohydrates, cofactors, peptides, and energy metabolites. Forty-six of the 64 associations were directionally consistent with those observed for changes *Pre*- and *Post*-season (i.e., metabolites that increased at *Post* also increased with the number of HAE and vice versa).

## Discussion

The current study aimed to identify individual metabolite changes in response to collegiate American football participation over a single season. Serum concentrations of 200 metabolites changed and we observed significant enrichment for metabolite members of five pathways. A subset of 12 metabolites was sufficient to accurately discriminate *Post*- from *Pre*-season status and 64 metabolites showed significant associations with HAEs. Our discussion focuses on key metabolic pathways and predictors. Hypotheses connecting other significant metabolites to the *Pre*- and *Post*-season transition are presented in Supplemental Table S2.

Caffeine metabolites (*Xanthine metabolism*) collectively presented with the relatively largest changes *Post*-season. Given known metabolite markers of coffee^25^ and tea^26^ consumption did not significantly change, athletes likely increased their caffeine intake through consumption of soda, energy drinks, or supplements. The over fivefold increase in serum saccharin may also suggest some of these sources were sugar-free. To our knowledge, Full Throttle is the only popular sugar-free energy drink containing saccharin in the US; all others are sweetened with acesulfame, aspartame, and/or sucralose^27^. Caffeine offers a potential edge in athletic performance and is likely the reason for increased caffeine intake by athletes in competition^28^. Because our athletes were not required to fast prior to blood collection, the marked increase in caffeine metabolite levels may reflect recent or habitual intakes. Accumulating evidence supports a protective role of caffeine against brain injury^29–31^. Whether habitual caffeine consumption benefits long-term neurological health of football athletes is unknown but warrants consideration.

Most of the assayed *Acyl choline fatty acids* decreased *Post*-season which represent novel findings in the context of chronic physical training and contact sports. Aside from acetylcholine (ACh, not assayed), our knowledge of other acyl cholines is limited. Based on ACh metabolism, we might infer the synthesis of other acyl cholines begins with the activation of the parent fatty acid by an acid-thiol ligase in the presence of coenzyme A and ATP and transfer to choline by choline acetylase^32^. Arachidonoylcholine, docosahexaenoylcholine, alpha-linoleoylcholine, and oleoylcholine, are reportedly weak to moderate inhibitors of the muscle-type and α7 nicotinic ACh receptor (nAChR)^33–35^. Since activated α7nAChR exhibits anti-inflammatory and neuroprotective properties, it is possible that the decreased serum levels of acyl choline fatty acids observed may be a benefit of physical training and exercise, that in turn may offer protection against HAE-related injury during a season of football.

**Figure 4:**
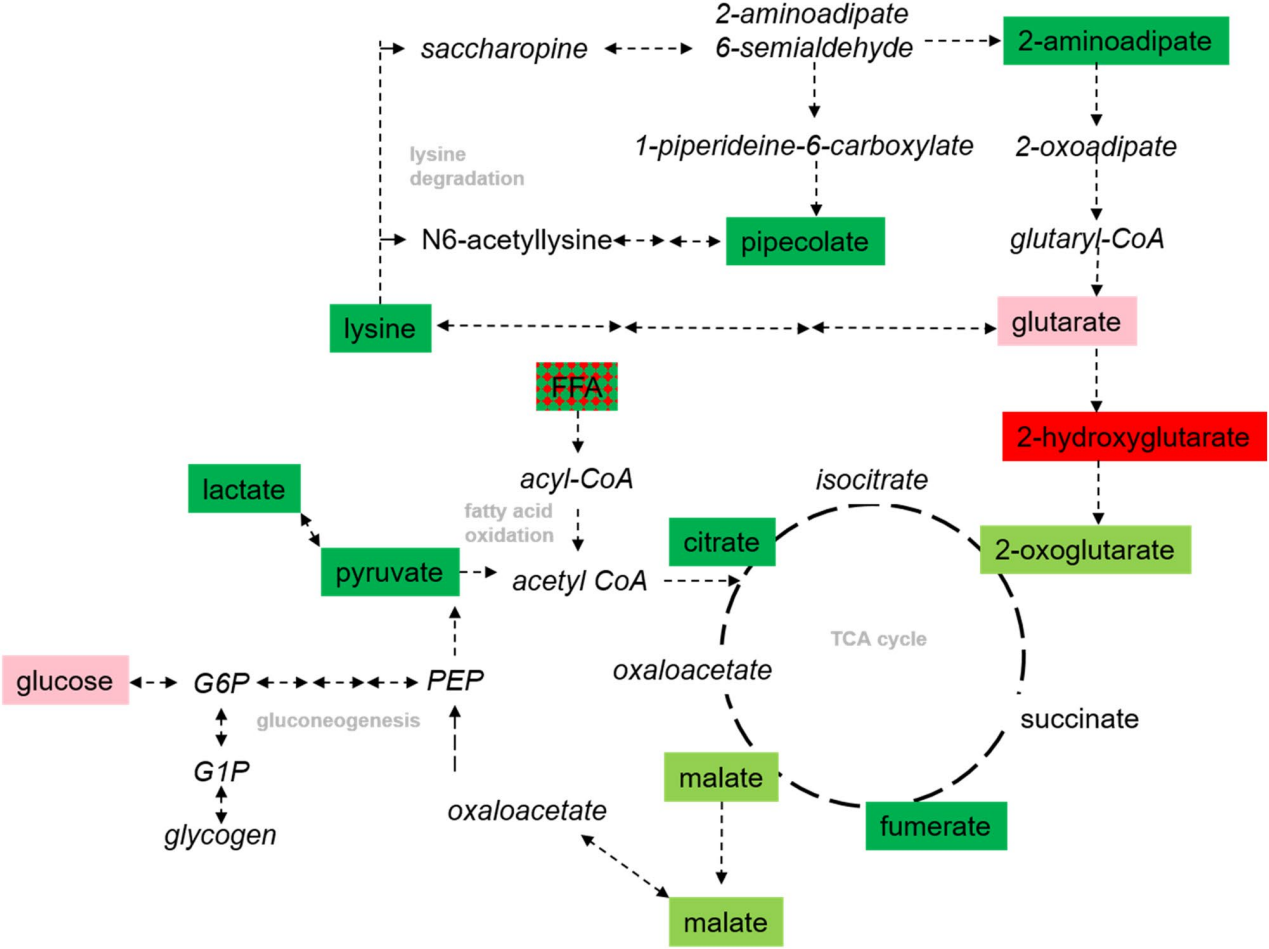
Shifts in energy metabolism from *Pre-* to *Post-*season. Metabolites that significantly (P < 0.05, Q < 0.05) increased and decreased from *Pre* to *Post* are displayed in red and green boxes, respectively. Corresponding but nominally significant (P < 0.05, Q > 0.05) metabolites are in light red and light green boxes. Metabolites in *italics* were not measured. Intersecting arrows (↔ ↔) imply additional metabolic steps not shown.

Altered *MCFA* and *Glycolysis, gluconeogenesis, and pyruvate metabolism* could reflect two etiologies: increased metabolic efficiency incurred with months of training and conditioning over the football season or mitochondrial beta-oxidation dysfunction which would contribute to the decrease in TCA cycle metabolites we observed. Glucose and fatty acids are the dominant fuels oxidized by the muscle for energy production during exercise and the contribution of these fuels can be influenced by diet, muscle glycogen content, exercise intensity, duration, and training status^36^. Pyruvate, an end product of the glycolysis pathway which feeds into the TCA cycle, lactate, and several TCA cycle intermediates decreased *Post*-season (Fig. 4). Most of the MCFA assayed in the current study decreased, while glycerol and long-chain fatty acids (LCFA) generally increased. These patterns of findings suggest a shift in reliance on glucose to fatty acids as a fuel source which also results in improved ability to maintain glycogen stores^36,37^. MCFA, in particular, provides a more readily usable energy source than LCFA, possibly due to enhanced cellular uptake and entry into the mitochondria^38,39^. Indeed, different rates of fatty acid oxidation might explain why serum MCFA decreased and LCFA increased. The former may result from a higher fatty acid oxidation rate than lipolysis rate and vice versa for LCFA. The increased levels of several oxidized medium and short chain (saturated) fatty acids would support this notion; though the saturation-specificity is more difficult to interpret. In particular, elevated LCFA may signal mitochondrial beta-oxidation dysfunction which would contribute to the decrease in TCA cycle metabolites we observed. LCFAs (7-hydroxyoctanoate, 8-hydroxyoctanoate, suberate, and sebacate) have been observed to increase in persons with medium chain acyl coenzyme A dehydrogenase deficiency, a rare genetic disorder characterized by dysfunctional mitochondrial beta oxidation^40,41^.

*Primary bile acids* are synthesized in the liver, actively secreted into bile and stored in the gallbladder. Bile is discharged into the intestinal lumen in the presence of food and thus bile levels increase after food intake and decrease with fasting^42^. In the current study, cholate and chenodeoxycholate increased while their conjugated metabolites generally decreased. A similar pattern was also observed for several secondary bile acids, which are formed by the intestinal microbiome when some of the primary bile acids enter the colon^42^. Apart from their role as digestive surfactants, bile acids have emerged as important signaling molecules with functions in systemic lipid metabolism, energy metabolism, immune homeostasis, and electrolyte balance^42^. Evidence also suggests that bile acids cross the blood–brain barrier where they might also function in neural health^43–45^.

Besides changes in the aforementioned metabolic pathways, serum levels of metabolites spanning multiple pathways related to glycerphospholipids also changed *Post*-season. Phosphatidylethanolamines (PEs) levels decreased while others assayed including phosphatidylcholines (PCs), phosphatidylinositols (PI), and phosphatidylserines (PS) did not significantly change. PE is the second most abundant phospholipid in human lipids, after PCs, and higher proportions are found in mitochondria than in other organelles^46^. The majority of PE is synthesized by the CDP-ethanolamine pathway in the endoplasmic reticulum and the PS decarboxylase pathway in mitochondrial inner membranes^46,47^. Metabolites of the former pathway including phosphoethanolamine, sphinganine-1-phosphate, and several diacylglycerols also decreased. Glycerophospholipids are important structural components of biological membranes^48^. Decreases in PE decrease the fluidity of the membrane and should theoretically compromise membrane integrity and potential^49^. In the mitochondria, PE also facilitates oxidative phosphorylation via the electron transport chain^46^. Endurance exercise training induces proliferation of skeletal muscle mitochondria and recent data suggests a parallel change in mitochondrial phospholipid composition whereby a disproportionate increase in PE occurs compared to other phospholipids^50^. As serum is free of mitochondria, we cannot extrapolate our findings to this organelle and thus the clinical significance of decreased serum PE levels observed in the current study is unknown but warrants further study. Interestingly, the activation of phospholipases that, in turn, degrade cell membranes, is among mechanisms that underlie the neurological damage that accompanies head injury^51^. Preliminary human and animal studies report lower plasma levels of PE but also PC and PI with brain injury occurrence^15^.

A season of competitive football elicited a plethora of indirect and systemic associations and thus it was not unexpected that a much smaller set of metabolites was sufficient to accurately discriminate periods of competitive play from periods of off-season rest. 2-hydoxyglutarate carried the most discriminative weight and was one of several fatty acid oxidative products that increased *Post*-season. 2-hydroxyglutarate is unique, however, since it is converted to 2-oxoglutarate which then enters the TCA cycle. Since 2-oxoglutarate and several other TCA cycle intermediates at least nominally decreased *Post*-season (Fig. 4), the accumulation of 2-hydroxyglutarate may again reflect greater reliance on fatty acid oxidation for energy. However, this metabolite is also an established oncometabolite that may increase in response to oxidative stress^52^ and thus an alternate mechanism linking this metabolite to football participation cannot be discounted.

Studies exploring exercise-induced alterations of the human metabolome have largely focused on the period up to 24 h after intense and prolonged exercise^53,54^. Although changes vary by protocol, generally lactate, pyruvate, TCA cycle intermediates, nucleotide degradation products, glycerol, fatty acids, acylcarnitines, and ketone bodies increase after exercise, whereas bile acids decrease. Concentrations of amino acids change in different directions; likely explained by their multiple functions and synthetic pathways^54^. With few exceptions, changes in the metabolome of football athletes after a season of competitive football did not mirror those observed shortly after exercise (Supplemental Table S2) and thus lend confidence to the approach taken to address our study objectives, such as the potential relationship of metabolomic change to contact. Potential metabolite markers of physical fatigue, overtraining, and muscle damage also did not significantly change^38^. Although cortisol, which increased, is often viewed as having a counter-productive role in exercise due to its catabolic nature, it also plays a key role in exercise adaptations such as stimulation of gluconeogenesis and lipolysis^55^. Urea, a by-product of protein catabolism, did not change. Serum steroid metabolite levels also increased, thus favoring an anabolic over a catabolic state *Post*-season.

As is typical of elite athletes, our sample of football athletes likely maintained some degree of fitness during the off-season and changes in the metabolome reflect football participation, per se. Although athlete anthropometry indices were not measured, creatinine, which serves as a rough measure of muscle mass^56^, did not significantly change. Recently, Koay et al.^57^ examined the metabolic effects of an 80-day aerobic and strength exercise intervention in newly enlisted male soldiers. While the sample and study design are somewhat similar to the current study, the intervention did not include physical contact. Few of their findings overlapped with those of the current study (Supplemental Table S2). Although comparable studies are limited, metabolomic patterns reported in the current study are likely unique to athletes engaged in football; a sport characterized by its high contact nature and accumulation of HAEs.

Forty-nine of the 200 metabolites that changed from *Pre* to *Post* also associated with HAE metrics and suggest that the metabolic changes observed might also be related to head impact events that players endure across the football season. The majority of associations involved HAEs exceeding 25G but not 80G; possibly explained by fewer hits exceeding 80G in this sample. Whether HAEs mediate/moderate *Pre*- and *Post*-season metabolite levels will be the topic of future investigations.

Although the paired-sample design and relatively homogenous sample with respect to age, sex, fitness, and football experience are key strengths of the current study, several weaknesses should be acknowledged in addition to those mentioned above. Given these were elite athletes enrolled in strict nutritional programs, they were not required to fast prior to blood collection. Some of the significant changes in metabolites, non-lipids in particular, may reflect changes in recent food intake prior to *Pre* and *Post* blood collections. Future work will seek to incorporate collection of food frequency diaries to better monitor dietary intake. Given the length of the football season, we cannot rule out an impact of time-varying factors that may induce significant associations. Supervised discriminative techniques tend to over-fit the data and thus our panel of predictive metabolites needs to be validated in an independent sample and further tested for specificity. The current analysis did not account for athlete differences in playing time; this variation will be examined in future analysis as more data become available. In this regard, others have compared metabolomic profiles of athletes sustaining head injuries to comparable healthy athletes, or correlated metabolomic profiles with athlete measures of external load^8,58,59^. These have highlighted altered blood or urine levels of specific fatty acids, phospholipids, steroids, bile acids, and metabolites of tyrosine and tryptophan metabolism; some of which we observe *Post*-season in the current analysis (see Supplemental Table S2). While the small sample size (N = 23) was adequately powered to detect larger metabolic differences, future work with larger samples may reveal additional, smaller differences.

In summary, our study provides a thorough analysis of the metabolomic changes in response to a full season of collegiate American football participation. While some results support athletes’ improved metabolic efficiency, others (i.e., significant metabolite-HAE relationships) suggest potential metabolic changes related to physical contact endured with football participation. The novel metabolites and candidate pathways we have identified may provide new insight to metabolic consequences of the physical training and contact endured with football participation.

## Supplementary Information

Supplementary Information.
