# Supplementary material for: Metabolomic response to collegiate football participation: Pre- and Post-season analysis
Source: Sci Rep. 2022 Feb 23;12:3091. doi: 10.1038/s41598-022-07079-6 (PMC8866500; doi:10.1038/s41598-022-07079-6)
Supplement: Supplementary file 1 — Supplementary Information. [file 41598_2022_7079_MOESM1_ESM.docx]

Supplemental Digital Content

Metabolomic response to collegiate football participation: *Pre*- and *Post*-season analysis

# SUPPLEMENTAL METHODS

## Head Acceleration Event (HAE) monitoring

HAEs were monitored at all contact practice sessions (max = 53) using BodiTrak’s Head Health Network sensor system. It should be noted that other studies have found the majority of head impacts occurred during practices and not games ^1^. Prior to each contact practice, sensors were individually mounted on the inner surface of each active player’s helmet, between the shell and the padding, using 3M VHB adhesive. Sensors were monitored throughout the season for integrity and functionality and outputs included peak translational acceleration (PTA; G-units) and impact location. Two G-unit thresholds, $\geq$25G to < 80G (25G) and $\geq$80G (80G), were selected based on both pilot data and previous reports of impacts related to brain health and injury ^2,3^. For each *i*-th athlete, HAEs were quantified as the cumulative number of hits exceeding the threshold *Th* = 25G and 80G (cHAE_25G,i_ and cHAE_80G,i_):

$${cHAE}_{Th,i}= \sum_{k=1}^{N} u\left( {PTA}_{k,i}-Th \right)$$

$$where u\left( x \right)=\left\{ \begin{matrix} 1 if x>0 \\ 0 if x \leq0 \end{matrix} \right.$$

The average number of hits exceeding 25G and 80G per session for *i*-th athlete (aHAE_25G,i_ and aHAE_80G,i_) is given as

$${aHAE}_{Th,i}=\frac{{cHAE}_{Th,i}}{{sessions}_{i}} .$$

## Metabolomics assay, data acquisition and processing (descriptions provided by Metabolon)

## *Data Quality: Instrument and Process Variability*

| ***QC Sample*** | ***Measurement*** | ***Median RSD*** |
| --- | --- | --- |
| Internal Standards | Instrument Variability | 5% |
| Endogenous Biochemicals | Total Process Variability | 8% |

Instrument variability was determined by calculating the median relative standard deviation (RSD) for the internal standards that were added to each sample prior to injection into the mass spectrometers. Overall process variability was determined by calculating the median RSD for all endogenous metabolites (i.e., non-instrument standards) present in 100% of the Client Matrix samples, which are technical replicates of pooled client samples. Overall process variability was determined by calculating the median RSD for all endogenous metabolites (i.e., non-instrument standards) present in the CMTRX technical replicates. Values for instrument and process variability meet Metabolon’s acceptance criteria as shown in the table above.

***Sample Accessioning*:** Following receipt, samples were inventoried and immediately stored at -80^o^C. Each sample received was accessioned into the Metabolon Laboratory Information Management System (LIMS) system and was assigned by the LIMS a unique identifier that was associated with the original source identifier only. This identifier was used to track all sample handling, tasks, results, etc. The samples (and all derived aliquots) were tracked by the LIMS system. All portions of any sample were automatically assigned their own unique identifiers by the LIMS when a new task was created; the relationship of these samples was also tracked. All samples were maintained at -80^o^C until processed.

***Sample Preparation*:** Samples were prepared using the automated MicroLab STAR® system from Hamilton Company. Several recovery standards were added prior to the first step in the extraction process for QC purposes. To remove protein, dissociate small molecules bound to protein or trapped in the precipitated protein matrix, and to recover chemically diverse metabolites, proteins were precipitated with methanol under vigorous shaking for 2 min (Glen Mills GenoGrinder 2000) followed by centrifugation. The resulting extract was divided into five fractions: two for analysis by two separate reverse phase (RP)/UPLC-MS/MS methods with positive ion mode electrospray ionization (ESI), one for analysis by RP/UPLC-MS/MS with negative ion mode ESI, one for analysis by HILIC/UPLC-MS/MS with negative ion mode ESI, and one sample was reserved for backup. Samples were placed briefly on a TurboVap® (Zymark) to remove the organic solvent. The sample extracts were stored overnight under nitrogen before preparation for analysis.

***QA/QC*:** Several types of controls were analyzed in concert with the experimental samples: a pooled matrix sample generated by taking a small volume of each experimental sample (or alternatively, use of a pool of well-characterized human plasma) served as a technical replicate throughout the data set; extracted water samples served as process blanks; and a cocktail of QC standards that were carefully chosen not to interfere with the measurement of endogenous compounds were spiked into every analyzed sample, allowed instrument performance monitoring and aided chromatographic alignment. The two tables below describe these QC samples and standards, respectively.

| **Type** | **Sample Description** | **Purpose** |
| --- | --- | --- |
| MTRX | Large pool of human plasma maintained by Metabolon that has been characterized extensively. | Assure that all aspects of the Metabolon process are operating within specifications. |
| CMTRX | Pool created by taking a small aliquot from every customer sample. | Assess the effect of a non-plasma matrix on the Metabolon process and distinguish biological variability from process variability. |
| PRCS | Aliquot of ultra-pure water | Process Blank used to assess the contribution to compound signals from the process. |
| SOLV | Aliquot of solvents used in extraction. | Solvent Blank used to segregate contamination sources in the extraction. |

| **Type** | **Standard Description** | **Purpose** |
| --- | --- | --- |
| RS | Recovery Standard | Assess variability and verify performance of extraction and instrumentation. |
| IS | Internal Standard | Assess variability and performance of instrument. |

Instrument variability was determined by calculating the median relative standard deviation (RSD) for the standards that were added to each sample prior to injection into the mass spectrometers. Overall process variability was determined by calculating the median RSD for all endogenous metabolites (i.e., non-instrument standards) present in 100% of the pooled matrix samples. Experimental samples were randomized across the platform run with QC samples spaced evenly among the injections, as outlined in the figure below.

For this process, a small aliquot of each client sample (colored cylinders) is pooled to create a CMTRX technical replicate sample (multi-colored cylinder), which is then injected periodically throughout the platform run. Variability among consistently detected biochemicals can be used to calculate an estimate of overall process and platform variability.

***Ultrahigh Performance Liquid Chromatography-Tandem Mass Spectroscopy (UPLC-MS/M*S):** All methods utilized a Waters ACQUITY ultra-performance liquid chromatography (UPLC) and a Thermo Scientific Q-Exactive high resolution/accurate mass spectrometer interfaced with a heated electrospray ionization (HESI-II) source and Orbitrap mass analyzer operated at 35,000 mass resolution. The sample extract was dried then reconstituted in solvents compatible to each of the four methods. Each reconstitution solvent contained a series of standards at fixed concentrations to ensure injection and chromatographic consistency. One aliquot was analyzed using acidic positive ion conditions, chromatographically optimized for more hydrophilic compounds. In this method, the extract was gradient eluted from a C18 column (Waters UPLC BEH C18-2.1x100 mm, 1.7 µm) using water and methanol, containing 0.05% perfluoropentanoic acid (PFPA) and 0.1% formic acid (FA). Another aliquot was also analyzed using acidic positive ion conditions, however it was chromatographically optimized for more hydrophobic compounds. In this method, the extract was gradient eluted from the same afore mentioned C18 column using methanol, acetonitrile, water, 0.05% PFPA and 0.01% FA and was operated at an overall higher organic content. Another aliquot was analyzed using basic negative ion optimized conditions using a separate dedicated C18 column. The basic extracts were gradient eluted from the column using methanol and water, however with 6.5mM Ammonium Bicarbonate at pH 8. The fourth aliquot was analyzed via negative ionization following elution from a HILIC column (Waters UPLC BEH Amide 2.1x150 mm, 1.7 µm) using a gradient consisting of water and acetonitrile with 10mM Ammonium Formate, pH 10.8. The MS analysis alternated between MS and data-dependent MSn scans using dynamic exclusion. The scan range varied slighted between methods but covered 70-1000 m/z. Raw data files are archived and extracted as described below.

***Bioinformatics*:** The informatics system consisted of four major components, the Laboratory Information Management System (LIMS), the data extraction and peak-identification software, data processing tools for QC and compound identification, and a collection of information interpretation and visualization tools for use by data analysts. The hardware and software foundations for these informatics components were the LAN backbone, and a database server running Oracle 10.2.0.1 Enterprise Edition.

***LIMS*:**  The purpose of the Metabolon LIMS system was to enable fully auditable laboratory automation through a secure, easy to use, and highly specialized system. The scope of the Metabolon LIMS system encompasses sample accessioning, sample preparation and instrumental analysis and reporting and advanced data analysis. All of the subsequent software systems are grounded in the LIMS data structures. It has been modified to leverage and interface with the in-house information extraction and data visualization systems, as well as third party instrumentation and data analysis software.

***Data Extraction and Compound Identification*:** Raw data was extracted, peak-identified and QC processed using Metabolon’s hardware and software. These systems are built on a web-service platform utilizing Microsoft’s .NET technologies, which run on high-performance application servers and fiber-channel storage arrays in clusters to provide active failover and load-balancing. Compounds were identified by comparison to library entries of purified standards or recurrent unknown entities. Metabolon maintains a library based on authenticated standards that contains the retention time/index (RI), mass to charge ratio (*m/z)*, and chromatographic data (including MS/MS spectral data) on all molecules present in the library. Furthermore, biochemical identifications are based on three criteria: retention index within a narrow RI window of the proposed identification, accurate mass match to the library +/- 10 ppm, and the MS/MS forward and reverse scores between the experimental data and authentic standards. The MS/MS scores are based on a comparison of the ions present in the experimental spectrum to the ions present in the library spectrum. While there may be similarities between these molecules based on one of these factors, the use of all three data points can be utilized to distinguish and differentiate biochemicals. More than 3300 commercially available purified standard compounds have been acquired and registered into LIMS for analysis on all platforms for determination of their analytical characteristics. Additional mass spectral entries have been created for structurally unnamed biochemicals, which have been identified by virtue of their recurrent nature (both chromatographic and mass spectral). These compounds have the potential to be identified by future acquisition of a matching purified standard or by classical structural analysis.

***Curation*:** A variety of curation procedures were carried out to ensure that a high quality data set was made available for statistical analysis and data interpretation. The QC and curation processes were designed to ensure accurate and consistent identification of true chemical entities, and to remove those representing system artifacts, mis-assignments, and background noise. Metabolon data analysts use proprietary visualization and interpretation software to confirm the consistency of peak identification among the various samples. Library matches for each compound were checked for each sample and corrected if necessary.

***Metabolite Quantification and Data Normalization*:** Peaks were quantified using area-under-the-curve. For studies that did not require more than one day of analysis, no normalization is necessary, other than for purposes of data visualization. In certain instances, biochemical data may have been normalized to an additional factor (e.g., cell counts, total protein as determined by Bradford assay, osmolality, etc.) to account for differences in metabolite levels due to differences in the amount of material present in each sample.

## Statistical analysis

***Correlation analysis****:* We computed both ordinary Pearson correlations and pairwise partial correlations to explore the latent relationships of changes in identified metabolites. For partial correlations, we used the shrinkage method for estimation ^6^ and statistical significance was determined by comparing the observed partial correlation coefficient to expected partial correlation coefficients from 1000 randomly permuted data sets representing the H_0_-distribution of no-correlation. The observed partial correlation coefficient was considered statistically significant if the P value obtained from this permutation test was <0.05.

***Random Forest (RF) analysis****:* RF analysis, a non-parametric technique unaffected by feature scale, was also implemented as a secondary multivariate analysis. The tree number of the forest was set to 100, the number of input variables tried for each node was the square root of the number of total variables (i.e. 33 for 1131 variables), and the minimum size of the terminal nodes was set to 2. Ten-fold CMV was employed 100 times to estimate the prediction ability with low bias and low variance. The Gini “score”, the scaled sum of votes derived from the trained trees for out-of-bag samples, was used as an indicator of how much the metabolite contributes to predictive accuracy. Because the standard RF technique does not account for sample pairing, *multilevel* RF was employed using the within-person data matrix generated for multilevel PCA.

# SUPPLEMENTAL TABLES AND FIGURES

# Table S1: Metabolites analyzed in the current study

| **Super**  **Pathway** | **Sub Pathway** | **Biochemical Name** |
| --- | --- | --- |
| Amino Acid | Alanine and Aspartate Metabolism | alanine |
|  | Alanine and Aspartate Metabolism | asparagine |
|  | Alanine and Aspartate Metabolism | aspartate |
|  | Alanine and Aspartate Metabolism | hydroxyasparagine** |
|  | Alanine and Aspartate Metabolism | N,N-dimethylalanine |
|  | Alanine and Aspartate Metabolism | N-acetylalanine |
|  | Alanine and Aspartate Metabolism | N-acetylasparagine |
|  | Alanine and Aspartate Metabolism | N-acetylaspartate (NAA) |
|  | Alanine and Aspartate Metabolism | N-carbamoylalanine |
|  | Creatine Metabolism | creatine |
|  | Creatine Metabolism | creatinine |
|  | Creatine Metabolism | guanidinoacetate |
|  | Glutamate Metabolism | 4-hydroxyglutamate |
|  | Glutamate Metabolism | alpha-ketoglutaramate* |
|  | Glutamate Metabolism | beta-citrylglutamate |
|  | Glutamate Metabolism | citramalate |
|  | Glutamate Metabolism | glutamate |
|  | Glutamate Metabolism | glutamate, gamma-methyl ester |
|  | Glutamate Metabolism | glutamine |
|  | Glutamate Metabolism | N-acetyl-aspartyl-glutamate (NAAG) |
|  | Glutamate Metabolism | N-acetylglutamate |
|  | Glutamate Metabolism | N-acetylglutamine |
|  | Glutamate Metabolism | pyroglutamine* |
|  | Glutamate Metabolism | S-1-pyrroline-5-carboxylate |
|  | Glutathione Metabolism | 2-aminobutyrate |
|  | Glutathione Metabolism | 2-hydroxybutyrate/2-hydroxyisobutyrate |
|  | Glutathione Metabolism | 5-oxoproline |
|  | Glutathione Metabolism | cys-gly, oxidized |
|  | Glutathione Metabolism | cysteine-glutathione disulfide |
|  | Glutathione Metabolism | cysteinylglycine |
|  | Glutathione Metabolism | cysteinylglycine disulfide* |
|  | Glycine, Serine and Threonine Metabolism | betaine |
|  | Glycine, Serine and Threonine Metabolism | dimethylglycine |
|  | Glycine, Serine and Threonine Metabolism | glycine |
|  | Glycine, Serine and Threonine Metabolism | N-acetylglycine |
|  | Glycine, Serine and Threonine Metabolism | N-acetylserine |
|  | Glycine, Serine and Threonine Metabolism | N-acetylthreonine |
|  | Glycine, Serine and Threonine Metabolism | sarcosine |
|  | Glycine, Serine and Threonine Metabolism | serine |
|  | Glycine, Serine and Threonine Metabolism | threonine |
|  | Guanidino and Acetamido Metabolism | 1-methylguanidine |
|  | Guanidino and Acetamido Metabolism | 4-guanidinobutanoate |
|  | Guanidino and Acetamido Metabolism | guanidinosuccinate |
|  | Histidine Metabolism | 1-methyl-4-imidazoleacetate |
|  | Histidine Metabolism | 1-methyl-5-imidazoleacetate |
|  | Histidine Metabolism | 1-methylhistidine |
|  | Histidine Metabolism | 1-ribosyl-imidazoleacetate* |
|  | Histidine Metabolism | 3-methylhistidine |
|  | Histidine Metabolism | formiminoglutamate |
|  | Histidine Metabolism | histidine |
|  | Histidine Metabolism | hydantoin-5-propionate |
|  | Histidine Metabolism | imidazole lactate |
|  | Histidine Metabolism | imidazole propionate |
|  | Histidine Metabolism | N-acetyl-1-methylhistidine* |
|  | Histidine Metabolism | N-acetyl-3-methylhistidine* |
|  | Histidine Metabolism | N-acetylcarnosine |
|  | Histidine Metabolism | N-acetylhistidine |
|  | Histidine Metabolism | trans-urocanate |
|  | Leucine, Isoleucine and Valine Metabolism | 1-carboxyethylisoleucine |
|  | Leucine, Isoleucine and Valine Metabolism | 1-carboxyethylleucine |
|  | Leucine, Isoleucine and Valine Metabolism | 1-carboxyethylvaline |
|  | Leucine, Isoleucine and Valine Metabolism | 2,3-dihydroxy-2-methylbutyrate |
|  | Leucine, Isoleucine and Valine Metabolism | 2-hydroxy-3-methylvalerate |
|  | Leucine, Isoleucine and Valine Metabolism | 2-ketocaprylate |
|  | Leucine, Isoleucine and Valine Metabolism | 2-methylbutyrylcarnitine (C5) |
|  | Leucine, Isoleucine and Valine Metabolism | 3-hydroxy-2-ethylpropionate |
|  | Leucine, Isoleucine and Valine Metabolism | 3-hydroxyisobutyrate |
|  | Leucine, Isoleucine and Valine Metabolism | 3-methyl-2-oxobutyrate |
|  | Leucine, Isoleucine and Valine Metabolism | 3-methyl-2-oxovalerate |
|  | Leucine, Isoleucine and Valine Metabolism | 3-methylglutaconate |
|  | Leucine, Isoleucine and Valine Metabolism | 3-methylglutarylcarnitine (2) |
|  | Leucine, Isoleucine and Valine Metabolism | 4-methyl-2-oxopentanoate |
|  | Leucine, Isoleucine and Valine Metabolism | alpha-hydroxyisocaproate |
|  | Leucine, Isoleucine and Valine Metabolism | alpha-hydroxyisovalerate |
|  | Leucine, Isoleucine and Valine Metabolism | beta-hydroxyisovalerate |
|  | Leucine, Isoleucine and Valine Metabolism | beta-hydroxyisovaleroylcarnitine |
|  | Leucine, Isoleucine and Valine Metabolism | ethylmalonate |
|  | Leucine, Isoleucine and Valine Metabolism | isobutyrylcarnitine (C4) |
|  | Leucine, Isoleucine and Valine Metabolism | isobutyrylglycine |
|  | Leucine, Isoleucine and Valine Metabolism | isoleucine |
|  | Leucine, Isoleucine and Valine Metabolism | isovalerate (i5:0) |
|  | Leucine, Isoleucine and Valine Metabolism | isovalerylcarnitine (C5) |
|  | Leucine, Isoleucine and Valine Metabolism | isovalerylglycine |
|  | Leucine, Isoleucine and Valine Metabolism | leucine |
|  | Leucine, Isoleucine and Valine Metabolism | methylsuccinate |
|  | Leucine, Isoleucine and Valine Metabolism | N-acetylisoleucine |
|  | Leucine, Isoleucine and Valine Metabolism | N-acetylleucine |
|  | Leucine, Isoleucine and Valine Metabolism | N-acetylvaline |
|  | Leucine, Isoleucine and Valine Metabolism | tiglylcarnitine (C5:1-DC) |
|  | Leucine, Isoleucine and Valine Metabolism | valine |
|  | Lysine Metabolism | 2-aminoadipate |
|  | Lysine Metabolism | 5-(galactosylhydroxy)-L-lysine |
|  | Lysine Metabolism | 5-hydroxylysine |
|  | Lysine Metabolism | 6-oxopiperidine-2-carboxylate |
|  | Lysine Metabolism | fructosyllysine |
|  | Lysine Metabolism | glutarylcarnitine (C5-DC) |
|  | Lysine Metabolism | hydroxy-N6,N6,N6-trimethyllysine* |
|  | Lysine Metabolism | lysine |
|  | Lysine Metabolism | N,N,N-trimethyl-5-aminovalerate |
|  | Lysine Metabolism | N2-acetyl,N6,N6-dimethyllysine |
|  | Lysine Metabolism | N2-acetyl,N6-methyllysine |
|  | Lysine Metabolism | N6,N6,N6-trimethyllysine |
|  | Lysine Metabolism | N6,N6-dimethyllysine |
|  | Lysine Metabolism | N6-acetyllysine |
|  | Lysine Metabolism | N6-methyllysine |
|  | Lysine Metabolism | N-acetyl-cadaverine |
|  | Lysine Metabolism | pipecolate |
|  | Methionine, Cysteine, SAM and Taurine Metabolism | 2,3-dihydroxy-5-methylthio-4-pentenoate (DMTPA)* |
|  | Methionine, Cysteine, SAM and Taurine Metabolism | 2-hydroxy-4-(methylthio)butanoic acid |
|  | Methionine, Cysteine, SAM and Taurine Metabolism | 5-methylthioribose** |
|  | Methionine, Cysteine, SAM and Taurine Metabolism | alpha-ketobutyrate |
|  | Methionine, Cysteine, SAM and Taurine Metabolism | cystathionine |
|  | Methionine, Cysteine, SAM and Taurine Metabolism | cysteine |
|  | Methionine, Cysteine, SAM and Taurine Metabolism | cysteine s-sulfate |
|  | Methionine, Cysteine, SAM and Taurine Metabolism | cysteine sulfinic acid |
|  | Methionine, Cysteine, SAM and Taurine Metabolism | cystine |
|  | Methionine, Cysteine, SAM and Taurine Metabolism | hypotaurine |
|  | Methionine, Cysteine, SAM and Taurine Metabolism | lanthionine |
|  | Methionine, Cysteine, SAM and Taurine Metabolism | methionine |
|  | Methionine, Cysteine, SAM and Taurine Metabolism | methionine sulfone |
|  | Methionine, Cysteine, SAM and Taurine Metabolism | methionine sulfoxide |
|  | Methionine, Cysteine, SAM and Taurine Metabolism | N-acetylmethionine |
|  | Methionine, Cysteine, SAM and Taurine Metabolism | N-acetylmethionine sulfoxide |
|  | Methionine, Cysteine, SAM and Taurine Metabolism | N-acetyltaurine |
|  | Methionine, Cysteine, SAM and Taurine Metabolism | N-formylmethionine |
|  | Methionine, Cysteine, SAM and Taurine Metabolism | N-methyltaurine |
|  | Methionine, Cysteine, SAM and Taurine Metabolism | S-carboxyethylcysteine |
|  | Methionine, Cysteine, SAM and Taurine Metabolism | S-methylcysteine |
|  | Methionine, Cysteine, SAM and Taurine Metabolism | S-methylcysteine sulfoxide |
|  | Methionine, Cysteine, SAM and Taurine Metabolism | S-methylmethionine |
|  | Methionine, Cysteine, SAM and Taurine Metabolism | succinoyltaurine |
|  | Methionine, Cysteine, SAM and Taurine Metabolism | taurine |
|  | Phenylalanine Metabolism | 1-carboxyethylphenylalanine |
|  | Phenylalanine Metabolism | 2-hydroxyphenylacetate |
|  | Phenylalanine Metabolism | N-acetylphenylalanine |
|  | Phenylalanine Metabolism | phenylalanine |
|  | Phenylalanine Metabolism | phenyllactate (PLA) |
|  | Phenylalanine Metabolism | phenylpyruvate |
|  | Polyamine Metabolism | (N(1) + N(8))-acetylspermidine |
|  | Polyamine Metabolism | 4-acetamidobutanoate |
|  | Polyamine Metabolism | 5-methylthioadenosine (MTA) |
|  | Polyamine Metabolism | acisoga |
|  | Polyamine Metabolism | N-acetyl-isoputreanine |
|  | Polyamine Metabolism | N-acetylputrescine |
|  | Polyamine Metabolism | spermidine |
|  | Tryptophan Metabolism | 3-indoxyl sulfate |
|  | Tryptophan Metabolism | 6-bromotryptophan |
|  | Tryptophan Metabolism | 8-methoxykynurenate |
|  | Tryptophan Metabolism | anthranilate |
|  | Tryptophan Metabolism | C-glycosyltryptophan |
|  | Tryptophan Metabolism | indoleacetate |
|  | Tryptophan Metabolism | indoleacetoylcarnitine* |
|  | Tryptophan Metabolism | indoleacetylglutamine |
|  | Tryptophan Metabolism | indolelactate |
|  | Tryptophan Metabolism | indolepropionate |
|  | Tryptophan Metabolism | kynurenate |
|  | Tryptophan Metabolism | kynurenine |
|  | Tryptophan Metabolism | N-acetylkynurenine (2) |
|  | Tryptophan Metabolism | N-acetyltryptophan |
|  | Tryptophan Metabolism | N-formylanthranilic acid |
|  | Tryptophan Metabolism | picolinate |
|  | Tryptophan Metabolism | serotonin |
|  | Tryptophan Metabolism | tryptophan |
|  | Tryptophan Metabolism | tryptophan betaine |
|  | Tryptophan Metabolism | xanthurenate |
|  | Tyrosine Metabolism | 1-carboxyethyltyrosine |
|  | Tyrosine Metabolism | 3-(4-hydroxyphenyl)lactate |
|  | Tyrosine Metabolism | 3-methoxytyramine sulfate |
|  | Tyrosine Metabolism | 3-methoxytyrosine |
|  | Tyrosine Metabolism | 4-hydroxyphenylacetatoylcarnitine |
|  | Tyrosine Metabolism | 4-hydroxyphenylpyruvate |
|  | Tyrosine Metabolism | 4-methoxyphenol sulfate |
|  | Tyrosine Metabolism | dopamine 3-O-sulfate |
|  | Tyrosine Metabolism | dopamine 4-sulfate |
|  | Tyrosine Metabolism | gentisate |
|  | Tyrosine Metabolism | N-acetyltyrosine |
|  | Tyrosine Metabolism | p-cresol glucuronide* |
|  | Tyrosine Metabolism | phenol sulfate |
|  | Tyrosine Metabolism | thyroxine |
|  | Tyrosine Metabolism | tyramine O-sulfate |
|  | Tyrosine Metabolism | tyrosine |
|  | Tyrosine Metabolism | vanillactate |
|  | Tyrosine Metabolism | vanillic alcohol sulfate |
|  | Tyrosine Metabolism | vanillylmandelate (VMA) |
|  | Urea cycle; Arginine and Proline Metabolism | 2-oxoarginine* |
|  | Urea cycle; Arginine and Proline Metabolism | 3-amino-2-piperidone |
|  | Urea cycle; Arginine and Proline Metabolism | argininate* |
|  | Urea cycle; Arginine and Proline Metabolism | arginine |
|  | Urea cycle; Arginine and Proline Metabolism | argininosuccinate |
|  | Urea cycle; Arginine and Proline Metabolism | citrulline |
|  | Urea cycle; Arginine and Proline Metabolism | dimethylarginine (SDMA + ADMA) |
|  | Urea cycle; Arginine and Proline Metabolism | homoarginine |
|  | Urea cycle; Arginine and Proline Metabolism | homocitrulline |
|  | Urea cycle; Arginine and Proline Metabolism | N,N,N-trimethyl-alanylproline betaine (TMAP) |
|  | Urea cycle; Arginine and Proline Metabolism | N-acetylarginine |
|  | Urea cycle; Arginine and Proline Metabolism | N-acetylcitrulline |
|  | Urea cycle; Arginine and Proline Metabolism | N-acetylproline |
|  | Urea cycle; Arginine and Proline Metabolism | N-delta-acetylornithine |
|  | Urea cycle; Arginine and Proline Metabolism | N-methylhydroxyproline** |
|  | Urea cycle; Arginine and Proline Metabolism | N-methylproline |
|  | Urea cycle; Arginine and Proline Metabolism | ornithine |
|  | Urea cycle; Arginine and Proline Metabolism | pro-hydroxy-pro |
|  | Urea cycle; Arginine and Proline Metabolism | proline |
|  | Urea cycle; Arginine and Proline Metabolism | trans-4-hydroxyproline |
|  | Urea cycle; Arginine and Proline Metabolism | urea |
| Carbohydrate | Advanced Glycation End-product | N6-carboxymethyllysine |
|  | Aminosugar Metabolism | erythronate* |
|  | Aminosugar Metabolism | glucuronate |
|  | Aminosugar Metabolism | N-acetylglucosamine/N-acetylgalactosamine |
|  | Aminosugar Metabolism | N-acetylglucosaminylasparagine |
|  | Aminosugar Metabolism | N-acetylneuraminate |
|  | Disaccharides and Oligosaccharides | sucrose |
|  | Fructose, Mannose and Galactose Metabolism | fructose |
|  | Fructose, Mannose and Galactose Metabolism | galactonate |
|  | Fructose, Mannose and Galactose Metabolism | mannitol/sorbitol |
|  | Fructose, Mannose and Galactose Metabolism | mannose |
|  | Glycogen Metabolism | maltose |
|  | Glycolysis, Gluconeogenesis, and Pyruvate Metabolism | 1,5-anhydroglucitol (1,5-AG) |
|  | Glycolysis, Gluconeogenesis, and Pyruvate Metabolism | glucose |
|  | Glycolysis, Gluconeogenesis, and Pyruvate Metabolism | glycerate |
|  | Glycolysis, Gluconeogenesis, and Pyruvate Metabolism | lactate |
|  | Glycolysis, Gluconeogenesis, and Pyruvate Metabolism | pyruvate |
|  | Pentose Metabolism | arabinose |
|  | Pentose Metabolism | arabitol/xylitol |
|  | Pentose Metabolism | arabonate/xylonate |
|  | Pentose Metabolism | ribitol |
|  | Pentose Metabolism | ribonate |
|  | Pentose Metabolism | ribose |
|  | Pentose Metabolism | ribulonate/xylulonate/lyxonate* |
|  | Pentose Metabolism | sedoheptulose |
|  | Pentose Metabolism | xylose |
| Cofactors and Vitamins | Ascorbate and Aldarate Metabolism | 2-O-methylascorbic acid |
|  | Ascorbate and Aldarate Metabolism | ascorbic acid 2-sulfate |
|  | Ascorbate and Aldarate Metabolism | ascorbic acid 3-sulfate* |
|  | Ascorbate and Aldarate Metabolism | gulonate* |
|  | Ascorbate and Aldarate Metabolism | oxalate (ethanedioate) |
|  | Ascorbate and Aldarate Metabolism | threonate |
|  | Hemoglobin and Porphyrin Metabolism | bilirubin (E,E)* |
|  | Hemoglobin and Porphyrin Metabolism | bilirubin (E,Z or Z,E)* |
|  | Hemoglobin and Porphyrin Metabolism | bilirubin (Z,Z) |
|  | Hemoglobin and Porphyrin Metabolism | biliverdin |
|  | Hemoglobin and Porphyrin Metabolism | heme |
|  | Hemoglobin and Porphyrin Metabolism | I-urobilinogen |
|  | Nicotinate and Nicotinamide Metabolism | 1-methylnicotinamide |
|  | Nicotinate and Nicotinamide Metabolism | N1-Methyl-2-pyridone-5-carboxamide |
|  | Nicotinate and Nicotinamide Metabolism | N1-Methyl-4-pyridone-3-carboxamide |
|  | Nicotinate and Nicotinamide Metabolism | nicotinamide |
|  | Nicotinate and Nicotinamide Metabolism | quinolinate |
|  | Nicotinate and Nicotinamide Metabolism | trigonelline (N'-methylnicotinate) |
|  | Pantothenate and CoA Metabolism | pantoate |
|  | Pantothenate and CoA Metabolism | pantothenate |
|  | Tocopherol Metabolism | alpha-tocopherol |
|  | Tocopherol Metabolism | delta-CEHC |
|  | Tocopherol Metabolism | gamma-CEHC |
|  | Tocopherol Metabolism | gamma-CEHC glucuronide* |
|  | Tocopherol Metabolism | gamma-tocopherol/beta-tocopherol |
|  | Vitamin A Metabolism | beta-cryptoxanthin |
|  | Vitamin A Metabolism | carotene diol (1) |
|  | Vitamin A Metabolism | carotene diol (2) |
|  | Vitamin A Metabolism | carotene diol (3) |
|  | Vitamin A Metabolism | retinol (Vitamin A) |
|  | Vitamin B6 Metabolism | pyridoxal |
|  | Vitamin B6 Metabolism | pyridoxate |
| Energy | Oxidative Phosphorylation | phosphate |
|  | TCA Cycle | 2-methylcitrate/homocitrate |
|  | TCA Cycle | aconitate [cis or trans] |
|  | TCA Cycle | alpha-ketoglutarate |
|  | TCA Cycle | citraconate/glutaconate |
|  | TCA Cycle | citrate |
|  | TCA Cycle | fumarate |
|  | TCA Cycle | malate |
|  | TCA Cycle | succinate |
|  | TCA Cycle | succinylcarnitine (C4-DC) |
| Lipid | Androgenic Steroids | 11beta-hydroxyandrosterone glucuronide |
|  | Androgenic Steroids | 16a-hydroxy DHEA 3-sulfate |
|  | Androgenic Steroids | 5alpha-androstan-3alpha,17alpha-diol monosulfate |
|  | Androgenic Steroids | 5alpha-androstan-3alpha,17beta-diol 17-glucuronide |
|  | Androgenic Steroids | 5alpha-androstan-3alpha,17beta-diol disulfate |
|  | Androgenic Steroids | 5alpha-androstan-3alpha,17beta-diol monosulfate (1) |
|  | Androgenic Steroids | 5alpha-androstan-3alpha,17beta-diol monosulfate (2) |
|  | Androgenic Steroids | 5alpha-androstan-3beta,17alpha-diol disulfate |
|  | Androgenic Steroids | 5alpha-androstan-3beta,17beta-diol disulfate |
|  | Androgenic Steroids | 5alpha-androstan-3beta,17beta-diol monosulfate (2) |
|  | Androgenic Steroids | andro steroid monosulfate C19H28O6S (1)* |
|  | Androgenic Steroids | androstenediol (3alpha, 17alpha) monosulfate (2) |
|  | Androgenic Steroids | androstenediol (3alpha, 17alpha) monosulfate (3) |
|  | Androgenic Steroids | androstenediol (3beta,17beta) disulfate (1) |
|  | Androgenic Steroids | androstenediol (3beta,17beta) disulfate (2) |
|  | Androgenic Steroids | androstenediol (3beta,17beta) monosulfate (1) |
|  | Androgenic Steroids | androstenediol (3beta,17beta) monosulfate (2) |
|  | Androgenic Steroids | androsterone glucuronide |
|  | Androgenic Steroids | androsterone sulfate |
|  | Androgenic Steroids | dehydroepiandrosterone sulfate (DHEA-S) |
|  | Androgenic Steroids | epiandrosterone sulfate |
|  | Androgenic Steroids | etiocholanolone glucuronide |
|  | Carnitine Metabolism | carnitine |
|  | Carnitine Metabolism | deoxycarnitine |
|  | Ceramide PEs | palmitoyl-sphingosine-phosphoethanolamine (d18:1/16:0) |
|  | Ceramides | ceramide (d18:1/14:0, d16:1/16:0)* |
|  | Ceramides | ceramide (d18:1/17:0, d17:1/18:0)* |
|  | Ceramides | ceramide (d18:2/24:1, d18:1/24:2)* |
|  | Ceramides | N-palmitoyl-heptadecasphingosine (d17:1/16:0)* |
|  | Ceramides | N-palmitoyl-sphingadienine (d18:2/16:0)* |
|  | Ceramides | N-palmitoyl-sphingosine (d18:1/16:0) |
|  | Ceramides | N-stearoyl-sphingadienine (d18:2/18:0)* |
|  | Ceramides | N-stearoyl-sphingosine (d18:1/18:0)* |
|  | Corticosteroids | corticosterone |
|  | Corticosteroids | cortisol |
|  | Corticosteroids | cortisone |
|  | Corticosteroids | cortolone glucuronide (1) |
|  | Diacylglycerol | diacylglycerol (14:0/18:1, 16:0/16:1) [2]* |
|  | Diacylglycerol | diacylglycerol (16:1/18:2 [2], 16:0/18:3 [1])* |
|  | Diacylglycerol | linoleoyl-arachidonoyl-glycerol (18:2/20:4) [1]* |
|  | Diacylglycerol | linoleoyl-arachidonoyl-glycerol (18:2/20:4) [2]* |
|  | Diacylglycerol | linoleoyl-docosahexaenoyl-glycerol (18:2/22:6) [1]* |
|  | Diacylglycerol | linoleoyl-docosahexaenoyl-glycerol (18:2/22:6) [2]* |
|  | Diacylglycerol | linoleoyl-linolenoyl-glycerol (18:2/18:3) [1]* |
|  | Diacylglycerol | linoleoyl-linolenoyl-glycerol (18:2/18:3) [2]* |
|  | Diacylglycerol | linoleoyl-linoleoyl-glycerol (18:2/18:2) [1]* |
|  | Diacylglycerol | oleoyl-arachidonoyl-glycerol (18:1/20:4) [1]* |
|  | Diacylglycerol | oleoyl-arachidonoyl-glycerol (18:1/20:4) [2]* |
|  | Diacylglycerol | oleoyl-linoleoyl-glycerol (18:1/18:2) [1] |
|  | Diacylglycerol | oleoyl-linoleoyl-glycerol (18:1/18:2) [2] |
|  | Diacylglycerol | oleoyl-oleoyl-glycerol (18:1/18:1) [1]* |
|  | Diacylglycerol | oleoyl-oleoyl-glycerol (18:1/18:1) [2]* |
|  | Diacylglycerol | palmitoleoyl-arachidonoyl-glycerol (16:1/20:4) [2]* |
|  | Diacylglycerol | palmitoleoyl-linoleoyl-glycerol (16:1/18:2) [1]* |
|  | Diacylglycerol | palmitoyl-arachidonoyl-glycerol (16:0/20:4) [1]* |
|  | Diacylglycerol | palmitoyl-arachidonoyl-glycerol (16:0/20:4) [2]* |
|  | Diacylglycerol | palmitoyl-docosahexaenoyl-glycerol (16:0/22:6) [1]* |
|  | Diacylglycerol | palmitoyl-linolenoyl-glycerol (16:0/18:3) [2]* |
|  | Diacylglycerol | palmitoyl-linoleoyl-glycerol (16:0/18:2) [1]* |
|  | Diacylglycerol | palmitoyl-linoleoyl-glycerol (16:0/18:2) [2]* |
|  | Diacylglycerol | palmitoyl-oleoyl-glycerol (16:0/18:1) [1]* |
|  | Diacylglycerol | palmitoyl-oleoyl-glycerol (16:0/18:1) [2]* |
|  | Diacylglycerol | stearoyl-arachidonoyl-glycerol (18:0/20:4) [1]* |
|  | Diacylglycerol | stearoyl-arachidonoyl-glycerol (18:0/20:4) [2]* |
|  | Dihydroceramides | N-palmitoyl-sphinganine (d18:0/16:0) |
|  | Dihydroceramides | N-stearoyl-sphinganine (d18:0/18:0)* |
|  | Dihydrosphingomyelins | behenoyl dihydrosphingomyelin (d18:0/22:0)* |
|  | Dihydrosphingomyelins | myristoyl dihydrosphingomyelin (d18:0/14:0)* |
|  | Dihydrosphingomyelins | palmitoyl dihydrosphingomyelin (d18:0/16:0)* |
|  | Dihydrosphingomyelins | sphingomyelin (d18:0/18:0, d19:0/17:0)* |
|  | Dihydrosphingomyelins | sphingomyelin (d18:0/20:0, d16:0/22:0)* |
|  | Eicosanoid | 12-HETE |
|  | Endocannabinoid | linoleoyl ethanolamide |
|  | Endocannabinoid | N-oleoylserine |
|  | Endocannabinoid | N-palmitoylserine |
|  | Endocannabinoid | N-stearoyltaurine |
|  | Endocannabinoid | oleoyl ethanolamide |
|  | Endocannabinoid | palmitoyl ethanolamide |
|  | Fatty Acid Metabolism (Acyl Carnitine, Dicarboxylate) | adipoylcarnitine (C6-DC) |
|  | Fatty Acid Metabolism (Acyl Carnitine, Dicarboxylate) | octadecanedioylcarnitine (C18-DC)* |
|  | Fatty Acid Metabolism (Acyl Carnitine, Dicarboxylate) | octadecenedioylcarnitine (C18:1-DC)* |
|  | Fatty Acid Metabolism (Acyl Carnitine, Dicarboxylate) | pimeloylcarnitine/3-methyladipoylcarnitine (C7-DC) |
|  | Fatty Acid Metabolism (Acyl Carnitine, Dicarboxylate) | suberoylcarnitine (C8-DC) |
|  | Fatty Acid Metabolism (Acyl Carnitine, Hydroxy) | (R)-3-hydroxybutyrylcarnitine |
|  | Fatty Acid Metabolism (Acyl Carnitine, Hydroxy) | (S)-3-hydroxybutyrylcarnitine |
|  | Fatty Acid Metabolism (Acyl Carnitine, Hydroxy) | 3-hydroxydecanoylcarnitine |
|  | Fatty Acid Metabolism (Acyl Carnitine, Hydroxy) | 3-hydroxyhexanoylcarnitine (1) |
|  | Fatty Acid Metabolism (Acyl Carnitine, Hydroxy) | 3-hydroxyoctanoylcarnitine (1) |
|  | Fatty Acid Metabolism (Acyl Carnitine, Hydroxy) | 3-hydroxyoctanoylcarnitine (2) |
|  | Fatty Acid Metabolism (Acyl Carnitine, Hydroxy) | 3-hydroxyoleoylcarnitine |
|  | Fatty Acid Metabolism (Acyl Carnitine, Hydroxy) | cis-3,4-methyleneheptanoylcarnitine |
|  | Fatty Acid Metabolism (Acyl Carnitine, Long Chain Saturated) | arachidoylcarnitine (C20)* |
|  | Fatty Acid Metabolism (Acyl Carnitine, Long Chain Saturated) | behenoylcarnitine (C22)* |
|  | Fatty Acid Metabolism (Acyl Carnitine, Long Chain Saturated) | cerotoylcarnitine (C26)* |
|  | Fatty Acid Metabolism (Acyl Carnitine, Long Chain Saturated) | lignoceroylcarnitine (C24)* |
|  | Fatty Acid Metabolism (Acyl Carnitine, Long Chain Saturated) | margaroylcarnitine (C17)* |
|  | Fatty Acid Metabolism (Acyl Carnitine, Long Chain Saturated) | myristoylcarnitine (C14) |
|  | Fatty Acid Metabolism (Acyl Carnitine, Long Chain Saturated) | palmitoylcarnitine (C16) |
|  | Fatty Acid Metabolism (Acyl Carnitine, Long Chain Saturated) | stearoylcarnitine (C18) |
|  | Fatty Acid Metabolism (Acyl Carnitine, Medium Chain) | decanoylcarnitine (C10) |
|  | Fatty Acid Metabolism (Acyl Carnitine, Medium Chain) | hexanoylcarnitine (C6) |
|  | Fatty Acid Metabolism (Acyl Carnitine, Medium Chain) | laurylcarnitine (C12) |
|  | Fatty Acid Metabolism (Acyl Carnitine, Medium Chain) | nonanoylcarnitine (C9) |
|  | Fatty Acid Metabolism (Acyl Carnitine, Medium Chain) | octanoylcarnitine (C8) |
|  | Fatty Acid Metabolism (Acyl Carnitine, Monounsaturated) | 3-decenoylcarnitine |
|  | Fatty Acid Metabolism (Acyl Carnitine, Monounsaturated) | cis-4-decenoylcarnitine (C10:1) |
|  | Fatty Acid Metabolism (Acyl Carnitine, Monounsaturated) | eicosenoylcarnitine (C20:1)* |
|  | Fatty Acid Metabolism (Acyl Carnitine, Monounsaturated) | myristoleoylcarnitine (C14:1)* |
|  | Fatty Acid Metabolism (Acyl Carnitine, Monounsaturated) | nervonoylcarnitine (C24:1)* |
|  | Fatty Acid Metabolism (Acyl Carnitine, Monounsaturated) | oleoylcarnitine (C18:1) |
|  | Fatty Acid Metabolism (Acyl Carnitine, Monounsaturated) | palmitoleoylcarnitine (C16:1)* |
|  | Fatty Acid Metabolism (Acyl Carnitine, Monounsaturated) | undecenoylcarnitine (C11:1) |
|  | Fatty Acid Metabolism (Acyl Carnitine, Monounsaturated) | ximenoylcarnitine (C26:1)* |
|  | Fatty Acid Metabolism (Acyl Carnitine, Polyunsaturated) | adrenoylcarnitine (C22:4)* |
|  | Fatty Acid Metabolism (Acyl Carnitine, Polyunsaturated) | arachidonoylcarnitine (C20:4) |
|  | Fatty Acid Metabolism (Acyl Carnitine, Polyunsaturated) | dihomo-linolenoylcarnitine (C20:3n3 or 6)* |
|  | Fatty Acid Metabolism (Acyl Carnitine, Polyunsaturated) | dihomo-linoleoylcarnitine (C20:2)* |
|  | Fatty Acid Metabolism (Acyl Carnitine, Polyunsaturated) | linolenoylcarnitine (C18:3)* |
|  | Fatty Acid Metabolism (Acyl Carnitine, Polyunsaturated) | linoleoylcarnitine (C18:2)* |
|  | Fatty Acid Metabolism (Acyl Carnitine, Short Chain) | acetylcarnitine (C2) |
|  | Fatty Acid Metabolism (Acyl Choline) | arachidonoylcholine |
|  | Fatty Acid Metabolism (Acyl Choline) | dihomo-linolenoyl-choline |
|  | Fatty Acid Metabolism (Acyl Choline) | docosahexaenoylcholine |
|  | Fatty Acid Metabolism (Acyl Choline) | eicosapentaenoylcholine |
|  | Fatty Acid Metabolism (Acyl Choline) | linoleoylcholine* |
|  | Fatty Acid Metabolism (Acyl Choline) | oleoylcholine |
|  | Fatty Acid Metabolism (Acyl Choline) | palmitoloelycholine |
|  | Fatty Acid Metabolism (Acyl Choline) | palmitoylcholine |
|  | Fatty Acid Metabolism (Acyl Choline) | stearoylcholine* |
|  | Fatty Acid Metabolism (Acyl Glutamine) | hexanoylglutamine |
|  | Fatty Acid Metabolism (Acyl Glycine) | 3-hydroxybutyroylglycine** |
|  | Fatty Acid Metabolism (Acyl Glycine) | cis-3,4-methyleneheptanoylglycine |
|  | Fatty Acid Metabolism (Acyl Glycine) | N-palmitoylglycine |
|  | Fatty Acid Metabolism (Acyl Glycine) | picolinoylglycine |
|  | Fatty Acid Metabolism (also BCAA Metabolism) | butyrylcarnitine (C4) |
|  | Fatty Acid Metabolism (also BCAA Metabolism) | butyrylglycine |
|  | Fatty Acid Metabolism (also BCAA Metabolism) | methylmalonate (MMA) |
|  | Fatty Acid Metabolism (also BCAA Metabolism) | propionylcarnitine (C3) |
|  | Fatty Acid Metabolism (also BCAA Metabolism) | propionylglycine |
|  | Fatty Acid, Amino | 2-aminoheptanoate |
|  | Fatty Acid, Amino | 2-aminooctanoate |
|  | Fatty Acid, Amino | N-acetyl-2-aminooctanoate* |
|  | Fatty Acid, Branched | (14 or 15)-methylpalmitate (a17:0 or i17:0) |
|  | Fatty Acid, Branched | (16 or 17)-methylstearate (a19:0 or i19:0) |
|  | Fatty Acid, Branched | cis-3,4-methyleneheptanoate |
|  | Fatty Acid, Dicarboxylate | 2-hydroxyadipate |
|  | Fatty Acid, Dicarboxylate | 2-hydroxyglutarate |
|  | Fatty Acid, Dicarboxylate | 2-hydroxysebacate |
|  | Fatty Acid, Dicarboxylate | 3-carboxy-4-methyl-5-pentyl-2-furanpropionate (3-CMPFP)** |
|  | Fatty Acid, Dicarboxylate | 3-carboxy-4-methyl-5-propyl-2-furanpropanoate (CMPF) |
|  | Fatty Acid, Dicarboxylate | 3-hydroxyadipate* |
|  | Fatty Acid, Dicarboxylate | 3-methyladipate |
|  | Fatty Acid, Dicarboxylate | azelate (C9-DC) |
|  | Fatty Acid, Dicarboxylate | branched chain 14:0 dicarboxylic acid** |
|  | Fatty Acid, Dicarboxylate | decadienedioic acid (C10:2-DC)** |
|  | Fatty Acid, Dicarboxylate | docosadioate (C22-DC) |
|  | Fatty Acid, Dicarboxylate | dodecadienoate (12:2)* |
|  | Fatty Acid, Dicarboxylate | dodecanedioate (C12-DC) |
|  | Fatty Acid, Dicarboxylate | dodecenedioate (C12:1-DC)* |
|  | Fatty Acid, Dicarboxylate | eicosanedioate (C20-DC) |
|  | Fatty Acid, Dicarboxylate | eicosenedioate (C20:1-DC)* |
|  | Fatty Acid, Dicarboxylate | glutarate (C5-DC) |
|  | Fatty Acid, Dicarboxylate | heptenedioate (C7:1-DC)* |
|  | Fatty Acid, Dicarboxylate | hexadecanedioate (C16-DC) |
|  | Fatty Acid, Dicarboxylate | hexadecenedioate (C16:1-DC)* |
|  | Fatty Acid, Dicarboxylate | hydroxy-CMPF* |
|  | Fatty Acid, Dicarboxylate | maleate |
|  | Fatty Acid, Dicarboxylate | octadecadienedioate (C18:2-DC)* |
|  | Fatty Acid, Dicarboxylate | octadecanedioate (C18-DC) |
|  | Fatty Acid, Dicarboxylate | octadecenedioate (C18:1-DC) |
|  | Fatty Acid, Dicarboxylate | pimelate (C7-DC) |
|  | Fatty Acid, Dicarboxylate | sebacate (C10-DC) |
|  | Fatty Acid, Dicarboxylate | suberate (C8-DC) |
|  | Fatty Acid, Dicarboxylate | tetradecadienedioate (C14:2-DC)* |
|  | Fatty Acid, Dicarboxylate | tetradecanedioate (C14-DC) |
|  | Fatty Acid, Dicarboxylate | tridecenedioate (C13:1-DC)* |
|  | Fatty Acid, Dicarboxylate | undecanedioate (C11-DC) |
|  | Fatty Acid, Dihydroxy | 12,13-DiHOME |
|  | Fatty Acid, Dihydroxy | 2R,3R-dihydroxybutyrate |
|  | Fatty Acid, Dihydroxy | 2S,3R-dihydroxybutyrate |
|  | Fatty Acid, Dihydroxy | 3,4-dihydroxybutyrate |
|  | Fatty Acid, Dihydroxy | 9,10-DiHOME |
|  | Fatty Acid, Monohydroxy | 13-HODE + 9-HODE |
|  | Fatty Acid, Monohydroxy | 16-hydroxypalmitate |
|  | Fatty Acid, Monohydroxy | 2-hydroxydecanoate |
|  | Fatty Acid, Monohydroxy | 2-hydroxynervonate* |
|  | Fatty Acid, Monohydroxy | 2-hydroxyoctanoate |
|  | Fatty Acid, Monohydroxy | 2-hydroxypalmitate |
|  | Fatty Acid, Monohydroxy | 2-hydroxystearate |
|  | Fatty Acid, Monohydroxy | 3-hydroxydecanoate |
|  | Fatty Acid, Monohydroxy | 3-hydroxyhexanoate |
|  | Fatty Acid, Monohydroxy | 3-hydroxyoctanoate |
|  | Fatty Acid, Monohydroxy | 3-hydroxysebacate |
|  | Fatty Acid, Monohydroxy | 5-hydroxyhexanoate |
|  | Fatty Acid, Monohydroxy | 7-hydroxyoctanoate |
|  | Fatty Acid, Monohydroxy | 8-hydroxyoctanoate |
|  | Fatty Acid, Monohydroxy | alpha-hydroxycaproate |
|  | Glycerolipid Metabolism | glycerol |
|  | Glycerolipid Metabolism | glycerol 3-phosphate |
|  | Glycerolipid Metabolism | glycerophosphoglycerol |
|  | Hexosylceramides (HCER) | glycosyl ceramide (d16:1/24:1, d18:1/22:1)* |
|  | Hexosylceramides (HCER) | glycosyl ceramide (d18:1/20:0, d16:1/22:0)* |
|  | Hexosylceramides (HCER) | glycosyl ceramide (d18:1/23:1, d17:1/24:1)* |
|  | Hexosylceramides (HCER) | glycosyl ceramide (d18:2/24:1, d18:1/24:2)* |
|  | Hexosylceramides (HCER) | glycosyl-N-(2-hydroxynervonoyl)-sphingosine (d18:1/24:1(2OH))* |
|  | Hexosylceramides (HCER) | glycosyl-N-behenoyl-sphingadienine (d18:2/22:0)* |
|  | Hexosylceramides (HCER) | glycosyl-N-palmitoyl-sphingosine (d18:1/16:0) |
|  | Hexosylceramides (HCER) | glycosyl-N-stearoyl-sphingosine (d18:1/18:0) |
|  | Hexosylceramides (HCER) | glycosyl-N-tricosanoyl-sphingadienine (d18:2/23:0)* |
|  | Inositol Metabolism | myo-inositol |
|  | Ketone Bodies | 3-hydroxybutyrate (BHBA) |
|  | Ketone Bodies | acetoacetate |
|  | Lactosylceramides (LCER) | lactosyl-N-behenoyl-sphingosine (d18:1/22:0)* |
|  | Lactosylceramides (LCER) | lactosyl-N-nervonoyl-sphingosine (d18:1/24:1)* |
|  | Lactosylceramides (LCER) | lactosyl-N-palmitoyl-sphingosine (d18:1/16:0) |
|  | Long Chain Monounsaturated Fatty Acid | 10-heptadecenoate (17:1n7) |
|  | Long Chain Monounsaturated Fatty Acid | 10-nonadecenoate (19:1n9) |
|  | Long Chain Monounsaturated Fatty Acid | eicosenoate (20:1) |
|  | Long Chain Monounsaturated Fatty Acid | erucate (22:1n9) |
|  | Long Chain Monounsaturated Fatty Acid | myristoleate (14:1n5) |
|  | Long Chain Monounsaturated Fatty Acid | nervonate (24:1n9)* |
|  | Long Chain Monounsaturated Fatty Acid | oleate/vaccenate (18:1) |
|  | Long Chain Monounsaturated Fatty Acid | palmitoleate (16:1n7) |
|  | Long Chain Polyunsaturated Fatty Acid (n3 and n6) | adrenate (22:4n6) |
|  | Long Chain Polyunsaturated Fatty Acid (n3 and n6) | arachidonate (20:4n6) |
|  | Long Chain Polyunsaturated Fatty Acid (n3 and n6) | dihomo-linoleate (20:2n6) |
|  | Long Chain Polyunsaturated Fatty Acid (n3 and n6) | dihomo-linolenate (20:3n3 or n6) |
|  | Long Chain Polyunsaturated Fatty Acid (n3 and n6) | docosadienoate (22:2n6) |
|  | Long Chain Polyunsaturated Fatty Acid (n3 and n6) | docosahexaenoate (DHA; 22:6n3) |
|  | Long Chain Polyunsaturated Fatty Acid (n3 and n6) | docosapentaenoate (n3 DPA; 22:5n3) |
|  | Long Chain Polyunsaturated Fatty Acid (n3 and n6) | docosapentaenoate (n6 DPA; 22:5n6) |
|  | Long Chain Polyunsaturated Fatty Acid (n3 and n6) | docosatrienoate (22:3n3) |
|  | Long Chain Polyunsaturated Fatty Acid (n3 and n6) | eicosapentaenoate (EPA; 20:5n3) |
|  | Long Chain Polyunsaturated Fatty Acid (n3 and n6) | hexadecadienoate (16:2n6) |
|  | Long Chain Polyunsaturated Fatty Acid (n3 and n6) | linoleate (18:2n6) |
|  | Long Chain Polyunsaturated Fatty Acid (n3 and n6) | linolenate [alpha or gamma; (18:3n3 or 6)] |
|  | Long Chain Polyunsaturated Fatty Acid (n3 and n6) | stearidonate (18:4n3) |
|  | Long Chain Polyunsaturated Fatty Acid (n3 and n6) | tetradecadienoate (14:2)* |
|  | Long Chain Saturated Fatty Acid | arachidate (20:0) |
|  | Long Chain Saturated Fatty Acid | behenate (22:0)* |
|  | Long Chain Saturated Fatty Acid | margarate (17:0) |
|  | Long Chain Saturated Fatty Acid | myristate (14:0) |
|  | Long Chain Saturated Fatty Acid | nonadecanoate (19:0) |
|  | Long Chain Saturated Fatty Acid | palmitate (16:0) |
|  | Long Chain Saturated Fatty Acid | pentadecanoate (15:0) |
|  | Long Chain Saturated Fatty Acid | stearate (18:0) |
|  | Lysophospholipid | 1-arachidonoyl-GPA (20:4) |
|  | Lysophospholipid | 1-arachidonoyl-GPC (20:4n6)* |
|  | Lysophospholipid | 1-arachidonoyl-GPE (20:4n6)* |
|  | Lysophospholipid | 1-arachidonoyl-GPI (20:4)* |
|  | Lysophospholipid | 1-cerotoyl-GPC (26:0)* |
|  | Lysophospholipid | 1-lignoceroyl-GPC (24:0) |
|  | Lysophospholipid | 1-linolenoyl-GPC (18:3)* |
|  | Lysophospholipid | 1-linoleoyl-GPA (18:2)* |
|  | Lysophospholipid | 1-linoleoyl-GPC (18:2) |
|  | Lysophospholipid | 1-linoleoyl-GPE (18:2)* |
|  | Lysophospholipid | 1-linoleoyl-GPG (18:2)* |
|  | Lysophospholipid | 1-linoleoyl-GPI (18:2)* |
|  | Lysophospholipid | 1-oleoyl-GPC (18:1) |
|  | Lysophospholipid | 1-oleoyl-GPE (18:1) |
|  | Lysophospholipid | 1-oleoyl-GPG (18:1)* |
|  | Lysophospholipid | 1-oleoyl-GPI (18:1) |
|  | Lysophospholipid | 1-palmitoleoyl-GPC (16:1)* |
|  | Lysophospholipid | 1-palmitoyl-GPA (16:0) |
|  | Lysophospholipid | 1-palmitoyl-GPC (16:0) |
|  | Lysophospholipid | 1-palmitoyl-GPE (16:0) |
|  | Lysophospholipid | 1-palmitoyl-GPG (16:0)* |
|  | Lysophospholipid | 1-palmitoyl-GPI (16:0) |
|  | Lysophospholipid | 1-stearoyl-GPC (18:0) |
|  | Lysophospholipid | 1-stearoyl-GPE (18:0) |
|  | Lysophospholipid | 1-stearoyl-GPI (18:0) |
|  | Lysophospholipid | 2-palmitoyl-GPC (16:0)* |
|  | Lysophospholipid | 2-stearoyl-GPE (18:0)* |
|  | Lysoplasmalogen | 1-(1-enyl-oleoyl)-GPE (P-18:1)* |
|  | Lysoplasmalogen | 1-(1-enyl-palmitoyl)-GPC (P-16:0)* |
|  | Lysoplasmalogen | 1-(1-enyl-palmitoyl)-GPE (P-16:0)* |
|  | Lysoplasmalogen | 1-(1-enyl-stearoyl)-GPE (P-18:0)* |
|  | Medium Chain Fatty Acid | (2 or 3)-decenoate (10:1n7 or n8) |
|  | Medium Chain Fatty Acid | 10-undecenoate (11:1n1) |
|  | Medium Chain Fatty Acid | 5-dodecenoate (12:1n7) |
|  | Medium Chain Fatty Acid | caprate (10:0) |
|  | Medium Chain Fatty Acid | caproate (6:0) |
|  | Medium Chain Fatty Acid | caprylate (8:0) |
|  | Medium Chain Fatty Acid | cis-4-decenoate (10:1n6)* |
|  | Medium Chain Fatty Acid | heptanoate (7:0) |
|  | Medium Chain Fatty Acid | laurate (12:0) |
|  | Medium Chain Fatty Acid | pelargonate (9:0) |
|  | Medium Chain Fatty Acid | undecanoate (11:0) |
|  | Mevalonate Metabolism | 3-hydroxy-3-methylglutarate |
|  | Monoacylglycerol | 1-arachidonylglycerol (20:4) |
|  | Monoacylglycerol | 1-dihomo-linolenylglycerol (20:3) |
|  | Monoacylglycerol | 1-linolenoylglycerol (18:3) |
|  | Monoacylglycerol | 1-linoleoylglycerol (18:2) |
|  | Monoacylglycerol | 1-oleoylglycerol (18:1) |
|  | Monoacylglycerol | 1-palmitoylglycerol (16:0) |
|  | Monoacylglycerol | 2-arachidonoylglycerol (20:4) |
|  | Monoacylglycerol | 2-linoleoylglycerol (18:2) |
|  | Monoacylglycerol | 2-myristoylglycerol (14:0) |
|  | Monoacylglycerol | 2-oleoylglycerol (18:1) |
|  | Monoacylglycerol | 2-palmitoylglycerol (16:0) |
|  | Phosphatidylcholine (PC) | 1,2-dilinoleoyl-GPC (18:2/18:2) |
|  | Phosphatidylcholine (PC) | 1,2-dipalmitoyl-GPC (16:0/16:0) |
|  | Phosphatidylcholine (PC) | 1-linoleoyl-2-arachidonoyl-GPC (18:2/20:4n6)* |
|  | Phosphatidylcholine (PC) | 1-linoleoyl-2-linolenoyl-GPC (18:2/18:3)* |
|  | Phosphatidylcholine (PC) | 1-myristoyl-2-arachidonoyl-GPC (14:0/20:4)* |
|  | Phosphatidylcholine (PC) | 1-myristoyl-2-palmitoyl-GPC (14:0/16:0) |
|  | Phosphatidylcholine (PC) | 1-oleoyl-2-docosahexaenoyl-GPC (18:1/22:6)* |
|  | Phosphatidylcholine (PC) | 1-palmitoleoyl-2-linolenoyl-GPC (16:1/18:3)* |
|  | Phosphatidylcholine (PC) | 1-palmitoyl-2-arachidonoyl-GPC (16:0/20:4n6) |
|  | Phosphatidylcholine (PC) | 1-palmitoyl-2-dihomo-linolenoyl-GPC (16:0/20:3n3 or 6)* |
|  | Phosphatidylcholine (PC) | 1-palmitoyl-2-docosahexaenoyl-GPC (16:0/22:6) |
|  | Phosphatidylcholine (PC) | 1-palmitoyl-2-linoleoyl-GPC (16:0/18:2) |
|  | Phosphatidylcholine (PC) | 1-palmitoyl-2-oleoyl-GPC (16:0/18:1) |
|  | Phosphatidylcholine (PC) | 1-palmitoyl-2-palmitoleoyl-GPC (16:0/16:1)* |
|  | Phosphatidylcholine (PC) | 1-palmitoyl-2-stearoyl-GPC (16:0/18:0) |
|  | Phosphatidylcholine (PC) | 1-stearoyl-2-arachidonoyl-GPC (18:0/20:4) |
|  | Phosphatidylcholine (PC) | 1-stearoyl-2-docosahexaenoyl-GPC (18:0/22:6) |
|  | Phosphatidylcholine (PC) | 1-stearoyl-2-linoleoyl-GPC (18:0/18:2)* |
|  | Phosphatidylcholine (PC) | 1-stearoyl-2-oleoyl-GPC (18:0/18:1) |
|  | Phosphatidylethanolamine (PE) | 1,2-dilinoleoyl-GPE (18:2/18:2)* |
|  | Phosphatidylethanolamine (PE) | 1-oleoyl-2-docosahexaenoyl-GPE (18:1/22:6)* |
|  | Phosphatidylethanolamine (PE) | 1-oleoyl-2-linoleoyl-GPE (18:1/18:2)* |
|  | Phosphatidylethanolamine (PE) | 1-palmitoyl-2-arachidonoyl-GPE (16:0/20:4)* |
|  | Phosphatidylethanolamine (PE) | 1-palmitoyl-2-docosahexaenoyl-GPE (16:0/22:6)* |
|  | Phosphatidylethanolamine (PE) | 1-palmitoyl-2-linoleoyl-GPE (16:0/18:2) |
|  | Phosphatidylethanolamine (PE) | 1-palmitoyl-2-oleoyl-GPE (16:0/18:1) |
|  | Phosphatidylethanolamine (PE) | 1-palmitoyl-2-stearoyl-GPE (16:0/18:0)* |
|  | Phosphatidylethanolamine (PE) | 1-stearoyl-2-arachidonoyl-GPE (18:0/20:4) |
|  | Phosphatidylethanolamine (PE) | 1-stearoyl-2-docosahexaenoyl-GPE (18:0/22:6)* |
|  | Phosphatidylethanolamine (PE) | 1-stearoyl-2-linoleoyl-GPE (18:0/18:2)* |
|  | Phosphatidylethanolamine (PE) | 1-stearoyl-2-oleoyl-GPE (18:0/18:1) |
|  | Phosphatidylinositol (PI) | 1-palmitoyl-2-arachidonoyl-GPI (16:0/20:4)* |
|  | Phosphatidylinositol (PI) | 1-palmitoyl-2-linoleoyl-GPI (16:0/18:2) |
|  | Phosphatidylinositol (PI) | 1-palmitoyl-2-oleoyl-GPI (16:0/18:1)* |
|  | Phosphatidylinositol (PI) | 1-stearoyl-2-arachidonoyl-GPI (18:0/20:4) |
|  | Phosphatidylinositol (PI) | 1-stearoyl-2-linoleoyl-GPI (18:0/18:2) |
|  | Phosphatidylinositol (PI) | 1-stearoyl-2-oleoyl-GPI (18:0/18:1)* |
|  | Phosphatidylserine (PS) | 1-stearoyl-2-oleoyl-GPS (18:0/18:1) |
|  | Phospholipid Metabolism | choline |
|  | Phospholipid Metabolism | choline phosphate |
|  | Phospholipid Metabolism | glycerophosphoethanolamine |
|  | Phospholipid Metabolism | glycerophosphoinositol* |
|  | Phospholipid Metabolism | glycerophosphorylcholine (GPC) |
|  | Phospholipid Metabolism | phosphoethanolamine |
|  | Phospholipid Metabolism | trimethylamine N-oxide |
|  | Plasmalogen | 1-(1-enyl-palmitoyl)-2-arachidonoyl-GPC (P-16:0/20:4)* |
|  | Plasmalogen | 1-(1-enyl-palmitoyl)-2-arachidonoyl-GPE (P-16:0/20:4)* |
|  | Plasmalogen | 1-(1-enyl-palmitoyl)-2-linoleoyl-GPC (P-16:0/18:2)* |
|  | Plasmalogen | 1-(1-enyl-palmitoyl)-2-linoleoyl-GPE (P-16:0/18:2)* |
|  | Plasmalogen | 1-(1-enyl-palmitoyl)-2-oleoyl-GPC (P-16:0/18:1)* |
|  | Plasmalogen | 1-(1-enyl-palmitoyl)-2-oleoyl-GPE (P-16:0/18:1)* |
|  | Plasmalogen | 1-(1-enyl-palmitoyl)-2-palmitoleoyl-GPC (P-16:0/16:1)* |
|  | Plasmalogen | 1-(1-enyl-palmitoyl)-2-palmitoyl-GPC (P-16:0/16:0)* |
|  | Plasmalogen | 1-(1-enyl-stearoyl)-2-arachidonoyl-GPE (P-18:0/20:4)* |
|  | Plasmalogen | 1-(1-enyl-stearoyl)-2-linoleoyl-GPE (P-18:0/18:2)* |
|  | Plasmalogen | 1-(1-enyl-stearoyl)-2-oleoyl-GPE (P-18:0/18:1) |
|  | Pregnenolone Steroids | 17alpha-hydroxypregnenolone 3-sulfate |
|  | Pregnenolone Steroids | 21-hydroxypregnenolone disulfate |
|  | Pregnenolone Steroids | 21-hydroxypregnenolone monosulfate (1) |
|  | Pregnenolone Steroids | pregnenediol disulfate (C21H34O8S2)* |
|  | Pregnenolone Steroids | pregnenediol sulfate (C21H34O5S)* |
|  | Pregnenolone Steroids | pregnenetriol disulfate* |
|  | Pregnenolone Steroids | pregnenetriol sulfate* |
|  | Pregnenolone Steroids | pregnenolone sulfate |
|  | Primary Bile Acid Metabolism | chenodeoxycholate |
|  | Primary Bile Acid Metabolism | cholate |
|  | Primary Bile Acid Metabolism | cholic acid glucuronide |
|  | Primary Bile Acid Metabolism | glyco-beta-muricholate** |
|  | Primary Bile Acid Metabolism | glycochenodeoxycholate |
|  | Primary Bile Acid Metabolism | glycochenodeoxycholate 3-sulfate |
|  | Primary Bile Acid Metabolism | glycochenodeoxycholate glucuronide (1) |
|  | Primary Bile Acid Metabolism | glycocholate |
|  | Primary Bile Acid Metabolism | tauro-beta-muricholate |
|  | Primary Bile Acid Metabolism | taurochenodeoxycholate |
|  | Primary Bile Acid Metabolism | taurocholate |
|  | Progestin Steroids | 5alpha-pregnan-3beta,20alpha-diol disulfate |
|  | Progestin Steroids | 5alpha-pregnan-3beta,20alpha-diol monosulfate (2) |
|  | Progestin Steroids | 5alpha-pregnan-3beta,20beta-diol monosulfate (1) |
|  | Progestin Steroids | 5alpha-pregnan-diol disulfate |
|  | Progestin Steroids | pregnanediol-3-glucuronide |
|  | Progestin Steroids | pregnanolone/allopregnanolone sulfate |
|  | Secondary Bile Acid Metabolism | deoxycholate |
|  | Secondary Bile Acid Metabolism | deoxycholic acid 12-sulfate* |
|  | Secondary Bile Acid Metabolism | deoxycholic acid glucuronide |
|  | Secondary Bile Acid Metabolism | glycocholenate sulfate* |
|  | Secondary Bile Acid Metabolism | glycodeoxycholate |
|  | Secondary Bile Acid Metabolism | glycodeoxycholate 3-sulfate |
|  | Secondary Bile Acid Metabolism | glycohyocholate |
|  | Secondary Bile Acid Metabolism | glycolithocholate |
|  | Secondary Bile Acid Metabolism | glycolithocholate sulfate* |
|  | Secondary Bile Acid Metabolism | glycoursodeoxycholate |
|  | Secondary Bile Acid Metabolism | glycoursodeoxycholic acid sulfate (1) |
|  | Secondary Bile Acid Metabolism | hyocholate |
|  | Secondary Bile Acid Metabolism | isoursodeoxycholate |
|  | Secondary Bile Acid Metabolism | lithocholate sulfate (1) |
|  | Secondary Bile Acid Metabolism | taurochenodeoxycholic acid 3-sulfate |
|  | Secondary Bile Acid Metabolism | taurocholenate sulfate* |
|  | Secondary Bile Acid Metabolism | taurodeoxycholate |
|  | Secondary Bile Acid Metabolism | taurodeoxycholic acid 3-sulfate |
|  | Secondary Bile Acid Metabolism | taurolithocholate 3-sulfate |
|  | Secondary Bile Acid Metabolism | ursodeoxycholate |
|  | Short Chain Fatty Acid | butyrate/isobutyrate (4:0) |
|  | Sphingolipid Synthesis | 3-ketosphinganine |
|  | Sphingolipid Synthesis | sphinganine |
|  | Sphingolipid Synthesis | sphinganine-1-phosphate |
|  | Sphingomyelins | behenoyl sphingomyelin (d18:1/22:0)* |
|  | Sphingomyelins | hydroxypalmitoyl sphingomyelin (d18:1/16:0(OH))** |
|  | Sphingomyelins | lignoceroyl sphingomyelin (d18:1/24:0) |
|  | Sphingomyelins | palmitoyl sphingomyelin (d18:1/16:0) |
|  | Sphingomyelins | sphingomyelin (d17:1/14:0, d16:1/15:0)* |
|  | Sphingomyelins | sphingomyelin (d17:1/16:0, d18:1/15:0, d16:1/17:0)* |
|  | Sphingomyelins | sphingomyelin (d17:2/16:0, d18:2/15:0)* |
|  | Sphingomyelins | sphingomyelin (d18:1/14:0, d16:1/16:0)* |
|  | Sphingomyelins | sphingomyelin (d18:1/17:0, d17:1/18:0, d19:1/16:0) |
|  | Sphingomyelins | sphingomyelin (d18:1/18:1, d18:2/18:0) |
|  | Sphingomyelins | sphingomyelin (d18:1/19:0, d19:1/18:0)* |
|  | Sphingomyelins | sphingomyelin (d18:1/20:0, d16:1/22:0)* |
|  | Sphingomyelins | sphingomyelin (d18:1/20:1, d18:2/20:0)* |
|  | Sphingomyelins | sphingomyelin (d18:1/20:2, d18:2/20:1, d16:1/22:2)* |
|  | Sphingomyelins | sphingomyelin (d18:1/21:0, d17:1/22:0, d16:1/23:0)* |
|  | Sphingomyelins | sphingomyelin (d18:1/22:1, d18:2/22:0, d16:1/24:1)* |
|  | Sphingomyelins | sphingomyelin (d18:1/22:2, d18:2/22:1, d16:1/24:2)* |
|  | Sphingomyelins | sphingomyelin (d18:1/24:1, d18:2/24:0)* |
|  | Sphingomyelins | sphingomyelin (d18:1/25:0, d19:0/24:1, d20:1/23:0, d19:1/24:0)* |
|  | Sphingomyelins | sphingomyelin (d18:2/14:0, d18:1/14:1)* |
|  | Sphingomyelins | sphingomyelin (d18:2/16:0, d18:1/16:1)* |
|  | Sphingomyelins | sphingomyelin (d18:2/18:1)* |
|  | Sphingomyelins | sphingomyelin (d18:2/21:0, d16:2/23:0)* |
|  | Sphingomyelins | sphingomyelin (d18:2/23:0, d18:1/23:1, d17:1/24:1)* |
|  | Sphingomyelins | sphingomyelin (d18:2/23:1)* |
|  | Sphingomyelins | sphingomyelin (d18:2/24:1, d18:1/24:2)* |
|  | Sphingomyelins | sphingomyelin (d18:2/24:2)* |
|  | Sphingomyelins | stearoyl sphingomyelin (d18:1/18:0) |
|  | Sphingomyelins | tricosanoyl sphingomyelin (d18:1/23:0)* |
|  | Sphingosines | hexadecasphingosine (d16:1)* |
|  | Sphingosines | sphingosine |
|  | Sphingosines | sphingosine 1-phosphate |
|  | Sterol | 3beta,7alpha-dihydroxy-5-cholestenoate |
|  | Sterol | 3beta-hydroxy-5-cholestenoate |
|  | Sterol | 4-cholesten-3-one |
|  | Sterol | 7-alpha-hydroxy-3-oxo-4-cholestenoate (7-Hoca) |
|  | Sterol | cholesterol |
| Unknown | N/A | X - 07765 |
|  | N/A | X - 11299 |
|  | N/A | X - 11308 |
|  | N/A | X - 11315 |
|  | N/A | X - 11372 |
|  | N/A | X - 11378 |
|  | N/A | X - 11381 |
|  | N/A | X - 11407 |
|  | N/A | X - 11441 - retired for bilirubin degradation product, C17H20N2O5 (1)** |
|  | N/A | X - 11442 - retired for bilirubin degradation product, C17H20N2O5 (2)** |
|  | N/A | X - 11444 |
|  | N/A | X - 11470 |
|  | N/A | X - 11478 |
|  | N/A | X - 11483 |
|  | N/A | X - 11522 - retired for bilirubin degradation product, C17H18N2O4 (2)** |
|  | N/A | X - 11530 - retired for bilirubin degradation product, C17H18N2O4 (3)** |
|  | N/A | X - 11787 |
|  | N/A | X - 11795 |
|  | N/A | X - 11843 |
|  | N/A | X - 11847 |
|  | N/A | X - 11849 |
|  | N/A | X - 11852 |
|  | N/A | X - 11858 |
|  | N/A | X - 11880 |
|  | N/A | X - 12007 |
|  | N/A | X - 12015 |
|  | N/A | X - 12026 |
|  | N/A | X - 12027 |
|  | N/A | X - 12096 |
|  | N/A | X - 12100 |
|  | N/A | X - 12101 |
|  | N/A | X - 12104 |
|  | N/A | X - 12111 |
|  | N/A | X - 12112 |
|  | N/A | X - 12117 |
|  | N/A | X - 12127 |
|  | N/A | X - 12193 |
|  | N/A | X - 12199 |
|  | N/A | X - 12216 |
|  | N/A | X - 12283 |
|  | N/A | X - 12410 |
|  | N/A | X - 12411 |
|  | N/A | X - 12456 |
|  | N/A | X - 12462 |
|  | N/A | X - 12544 |
|  | N/A | X - 12680 |
|  | N/A | X - 12707 |
|  | N/A | X - 12714 |
|  | N/A | X - 12729 |
|  | N/A | X - 12740 |
|  | N/A | X - 12753 |
|  | N/A | X - 12798 |
|  | N/A | X - 12812 |
|  | N/A | X - 12822 |
|  | N/A | X - 12830 |
|  | N/A | X - 12844 |
|  | N/A | X - 12847 |
|  | N/A | X - 12849 |
|  | N/A | X - 12906 |
|  | N/A | X - 13007 |
|  | N/A | X - 13431 |
|  | N/A | X - 13507 |
|  | N/A | X - 13658 |
|  | N/A | X - 13684 |
|  | N/A | X - 13695 |
|  | N/A | X - 13728 |
|  | N/A | X - 13729 |
|  | N/A | X - 13835 |
|  | N/A | X - 13866 |
|  | N/A | X - 14056 |
|  | N/A | X - 14904 |
|  | N/A | X - 14939 |
|  | N/A | X - 15220 |
|  | N/A | X - 15245 |
|  | N/A | X - 15461 |
|  | N/A | X - 15486 |
|  | N/A | X - 15492 - retired for urocortisol glucuronide (4) |
|  | N/A | X - 15503 |
|  | N/A | X - 15666 |
|  | N/A | X - 15674 |
|  | N/A | X - 15728 |
|  | N/A | X - 16087 |
|  | N/A | X - 16245 |
|  | N/A | X - 16397 |
|  | N/A | X - 16576 |
|  | N/A | X - 16580 |
|  | N/A | X - 16649 |
|  | N/A | X - 16935 |
|  | N/A | X - 16946 - retired for bilirubin degradation product, C16H18N2O5 (2)** |
|  | N/A | X - 16964 |
|  | N/A | X - 17146 |
|  | N/A | X - 17185 |
|  | N/A | X - 17325 |
|  | N/A | X - 17328 |
|  | N/A | X - 17335 |
|  | N/A | X - 17340 |
|  | N/A | X - 17343 |
|  | N/A | X - 17357 |
|  | N/A | X - 17438 |
|  | N/A | X - 17653 |
|  | N/A | X - 17676 |
|  | N/A | X - 17685 |
|  | N/A | X - 17690 |
|  | N/A | X - 18779 |
|  | N/A | X - 18886 |
|  | N/A | X - 18887 |
|  | N/A | X - 18899 |
|  | N/A | X - 18901 |
|  | N/A | X - 18913 |
|  | N/A | X - 18921 |
|  | N/A | X - 18922 |
|  | N/A | X - 19141 |
|  | N/A | X - 19299 |
|  | N/A | X - 19438 |
|  | N/A | X - 21258 |
|  | N/A | X - 21285 |
|  | N/A | X - 21286 |
|  | N/A | X - 21310 |
|  | N/A | X - 21312 |
|  | N/A | X - 21319 |
|  | N/A | X - 21339 |
|  | N/A | X - 21353 |
|  | N/A | X - 21364 |
|  | N/A | X - 21383 |
|  | N/A | X - 21441 |
|  | N/A | X - 21467 |
|  | N/A | X - 21470 |
|  | N/A | X - 21471 |
|  | N/A | X - 21607 |
|  | N/A | X - 21628 |
|  | N/A | X - 21736 |
|  | N/A | X - 21796 |
|  | N/A | X - 21829 |
|  | N/A | X - 21834 |
|  | N/A | X - 21845 |
|  | N/A | X - 22143 |
|  | N/A | X - 22162 |
|  | N/A | X - 22771 |
|  | N/A | X - 22834 |
|  | N/A | X - 23587 |
|  | N/A | X - 23593 |
|  | N/A | X - 23639 |
|  | N/A | X - 23641 |
|  | N/A | X - 23644 |
|  | N/A | X - 23648 |
|  | N/A | X - 23659 |
|  | N/A | X - 23662 |
|  | N/A | X - 23665 |
|  | N/A | X - 23666 |
|  | N/A | X - 23678 |
|  | N/A | X - 23680 |
|  | N/A | X - 23739 |
|  | N/A | X - 23780 |
|  | N/A | X - 23782 |
|  | N/A | X - 23786 |
|  | N/A | X - 23787 |
|  | N/A | X - 23997 |
|  | N/A | X - 24243 |
|  | N/A | X - 24295 |
|  | N/A | X - 24309 |
|  | N/A | X - 24328 |
|  | N/A | X - 24337 |
|  | N/A | X - 24411 |
|  | N/A | X - 24418 |
|  | N/A | X - 24422 |
|  | N/A | X - 24425 |
|  | N/A | X - 24431 |
|  | N/A | X - 24432 |
|  | N/A | X - 24435 |
|  | N/A | X - 24456 |
|  | N/A | X - 24473 |
|  | N/A | X - 24475 |
|  | N/A | X - 24494 |
|  | N/A | X - 24544 |
|  | N/A | X - 24546 |
|  | N/A | X - 24549 |
|  | N/A | X - 24556 |
|  | N/A | X - 24588 |
|  | N/A | X - 24699 |
|  | N/A | X - 24747 |
|  | N/A | X - 24748 |
|  | N/A | X - 24812 |
|  | N/A | X - 24849 - retired for bilirubin degradation product, C17H18N2O4 (1)** |
|  | N/A | X - 24928 |
|  | N/A | X - 24947 |
|  | N/A | X - 24949 |
|  | N/A | X - 24951 |
|  | N/A | X - 24953 |
|  | N/A | X - 25172 |
|  | N/A | X - 25217 |
|  | N/A | X - 25271 |
|  | N/A | X - 25343 |
|  | N/A | X - 25371 |
|  | N/A | X - 25397 |
|  | N/A | X - 25402 |
|  | N/A | X - 25407 |
|  | N/A | X - 25417 |
|  | N/A | X - 25419 |
|  | N/A | X - 25420 |
|  | N/A | X - 25422 |
|  | N/A | X - 25452 |
|  | N/A | X - 25519 |
|  | N/A | X - 25520 |
|  | N/A | X - 25790 |
|  | N/A | X - 25810 |
|  | N/A | X - 25828 |
|  | N/A | X - 25936 |
|  | N/A | X - 25948 |
|  | N/A | X - 25957 |
| Nucleotide | Purine Metabolism, (Hypo)Xanthine/Inosine containing | allantoin |
|  | Purine Metabolism, (Hypo)Xanthine/Inosine containing | hypoxanthine |
|  | Purine Metabolism, (Hypo)Xanthine/Inosine containing | inosine |
|  | Purine Metabolism, (Hypo)Xanthine/Inosine containing | N1-methylinosine |
|  | Purine Metabolism, (Hypo)Xanthine/Inosine containing | urate |
|  | Purine Metabolism, (Hypo)Xanthine/Inosine containing | xanthine |
|  | Purine Metabolism, Adenine containing | adenine |
|  | Purine Metabolism, Adenine containing | adenosine |
|  | Purine Metabolism, Adenine containing | adenosine 5'-monophosphate (AMP) |
|  | Purine Metabolism, Adenine containing | N1-methyladenosine |
|  | Purine Metabolism, Adenine containing | N6-carbamoylthreonyladenosine |
|  | Purine Metabolism, Adenine containing | N6-methyladenosine |
|  | Purine Metabolism, Guanine containing | 7-methylguanine |
|  | Purine Metabolism, Guanine containing | guanine |
|  | Purine Metabolism, Guanine containing | guanosine |
|  | Purine Metabolism, Guanine containing | N2,N2-dimethylguanosine |
|  | Pyrimidine Metabolism, Cytidine containing | 2'-O-methylcytidine |
|  | Pyrimidine Metabolism, Cytidine containing | 3-methylcytidine |
|  | Pyrimidine Metabolism, Cytidine containing | cytidine |
|  | Pyrimidine Metabolism, Cytidine containing | cytosine |
|  | Pyrimidine Metabolism, Cytidine containing | N4-acetylcytidine |
|  | Pyrimidine Metabolism, Orotate containing | dihydroorotate |
|  | Pyrimidine Metabolism, Orotate containing | orotate |
|  | Pyrimidine Metabolism, Orotate containing | orotidine |
|  | Pyrimidine Metabolism, Thymine containing | 3-aminoisobutyrate |
|  | Pyrimidine Metabolism, Thymine containing | 5,6-dihydrothymine |
|  | Pyrimidine Metabolism, Uracil containing | 2'-deoxyuridine |
|  | Pyrimidine Metabolism, Uracil containing | 2'-O-methyluridine |
|  | Pyrimidine Metabolism, Uracil containing | 3-(3-amino-3-carboxypropyl)uridine* |
|  | Pyrimidine Metabolism, Uracil containing | 3-ureidopropionate |
|  | Pyrimidine Metabolism, Uracil containing | 5,6-dihydrouridine |
|  | Pyrimidine Metabolism, Uracil containing | 5-methyluridine (ribothymidine) |
|  | Pyrimidine Metabolism, Uracil containing | beta-alanine |
|  | Pyrimidine Metabolism, Uracil containing | N-acetyl-beta-alanine |
|  | Pyrimidine Metabolism, Uracil containing | pseudouridine |
|  | Pyrimidine Metabolism, Uracil containing | uracil |
|  | Pyrimidine Metabolism, Uracil containing | uridine |
| Partially Characterized Molecules | Partially Characterized Molecules | branched-chain, straight-chain, or cyclopropyl 10:1 fatty acid (1)* |
|  | Partially Characterized Molecules | branched-chain, straight-chain, or cyclopropyl 10:1 fatty acid (2)* |
|  | Partially Characterized Molecules | branched-chain, straight-chain, or cyclopropyl 12:1 fatty acid* |
|  | Partially Characterized Molecules | glucuronide of C10H18O2 (1)* |
|  | Partially Characterized Molecules | glucuronide of C10H18O2 (7)* |
|  | Partially Characterized Molecules | glucuronide of C12H22O4 (1)* |
|  | Partially Characterized Molecules | glutamine conjugate of C6H10O2 (1)* |
|  | Partially Characterized Molecules | glutamine conjugate of C6H10O2 (2)* |
|  | Partially Characterized Molecules | glutamine conjugate of C7H12O2* |
|  | Partially Characterized Molecules | glycine conjugate of C10H12O2* |
|  | Partially Characterized Molecules | glycine conjugate of C10H14O2 (1)* |
|  | Partially Characterized Molecules | metabolonic lactone sulfate |
|  | Partially Characterized Molecules | pentose acid* |
| Peptide | Acetylated Peptides | phenylacetylcarnitine |
|  | Acetylated Peptides | phenylacetylglutamine |
|  | Dipeptide | glycylvaline |
|  | Dipeptide | isoleucylglycine |
|  | Dipeptide | leucylglycine |
|  | Dipeptide | phenylalanylalanine |
|  | Dipeptide | prolylglycine |
|  | Dipeptide | threonylphenylalanine |
|  | Dipeptide | valylglycine |
|  | Fibrinogen Cleavage Peptide | Fibrinopeptide A (2-15)** |
|  | Fibrinogen Cleavage Peptide | Fibrinopeptide A (3-15)** |
|  | Fibrinogen Cleavage Peptide | Fibrinopeptide A (3-16)** |
|  | Fibrinogen Cleavage Peptide | Fibrinopeptide A (4-15)** |
|  | Fibrinogen Cleavage Peptide | Fibrinopeptide A (5-16)* |
|  | Fibrinogen Cleavage Peptide | Fibrinopeptide A (7-16)* |
|  | Fibrinogen Cleavage Peptide | Fibrinopeptide A (8-16)** |
|  | Fibrinogen Cleavage Peptide | Fibrinopeptide A* |
|  | Fibrinogen Cleavage Peptide | Fibrinopeptide A, des-ala(1)* |
|  | Fibrinogen Cleavage Peptide | Fibrinopeptide A, phosphono-ser(3)* |
|  | Fibrinogen Cleavage Peptide | Fibrinopeptide B (1-11)** |
|  | Fibrinogen Cleavage Peptide | Fibrinopeptide B (1-12)** |
|  | Fibrinogen Cleavage Peptide | Fibrinopeptide B (1-13)** |
|  | Fibrinogen Cleavage Peptide | Fibrinopeptide B (1-9)** |
|  | Gamma-glutamyl Amino Acid | gamma-glutamyl-2-aminobutyrate |
|  | Gamma-glutamyl Amino Acid | gamma-glutamylalanine |
|  | Gamma-glutamyl Amino Acid | gamma-glutamyl-alpha-lysine |
|  | Gamma-glutamyl Amino Acid | gamma-glutamylcitrulline* |
|  | Gamma-glutamyl Amino Acid | gamma-glutamyl-epsilon-lysine |
|  | Gamma-glutamyl Amino Acid | gamma-glutamylglutamate |
|  | Gamma-glutamyl Amino Acid | gamma-glutamylglutamine |
|  | Gamma-glutamyl Amino Acid | gamma-glutamylglycine |
|  | Gamma-glutamyl Amino Acid | gamma-glutamylhistidine |
|  | Gamma-glutamyl Amino Acid | gamma-glutamylisoleucine* |
|  | Gamma-glutamyl Amino Acid | gamma-glutamylleucine |
|  | Gamma-glutamyl Amino Acid | gamma-glutamylmethionine |
|  | Gamma-glutamyl Amino Acid | gamma-glutamylphenylalanine |
|  | Gamma-glutamyl Amino Acid | gamma-glutamylthreonine |
|  | Gamma-glutamyl Amino Acid | gamma-glutamyltryptophan |
|  | Gamma-glutamyl Amino Acid | gamma-glutamyltyrosine |
|  | Gamma-glutamyl Amino Acid | gamma-glutamylvaline |
|  | Polypeptide | glu-gly-asn-val** |
|  | Polypeptide | HWESASXX* |
| Xenobiotics | Bacterial/Fungal | N-methylpipecolate |
|  | Benzoate Metabolism | 2-hydroxyhippurate (salicylurate) |
|  | Benzoate Metabolism | 3-(3-hydroxyphenyl)propionate |
|  | Benzoate Metabolism | 3-hydroxyhippurate |
|  | Benzoate Metabolism | 3-methyl catechol sulfate (1) |
|  | Benzoate Metabolism | 3-methyl catechol sulfate (2) |
|  | Benzoate Metabolism | 3-phenylpropionate (hydrocinnamate) |
|  | Benzoate Metabolism | 4-acetylphenol sulfate |
|  | Benzoate Metabolism | 4-allylcatechol sulfate |
|  | Benzoate Metabolism | 4-ethylcatechol sulfate |
|  | Benzoate Metabolism | 4-ethylphenylsulfate |
|  | Benzoate Metabolism | 4-hydroxyhippurate |
|  | Benzoate Metabolism | 4-methylcatechol sulfate |
|  | Benzoate Metabolism | 4-methylguaiacol sulfate |
|  | Benzoate Metabolism | 4-vinylphenol sulfate |
|  | Benzoate Metabolism | benzoate |
|  | Benzoate Metabolism | catechol sulfate |
|  | Benzoate Metabolism | guaiacol sulfate |
|  | Benzoate Metabolism | hippurate |
|  | Benzoate Metabolism | methyl-4-hydroxybenzoate sulfate |
|  | Benzoate Metabolism | o-cresol sulfate |
|  | Benzoate Metabolism | p-cresol sulfate |
|  | Benzoate Metabolism | propyl 4-hydroxybenzoate sulfate |
|  | Chemical | (2-butoxyethoxy)acetic acid |
|  | Chemical | 1,2,3-benzenetriol sulfate (2) |
|  | Chemical | 2,2'-Methylenebis(6-tert-butyl-p-cresol) |
|  | Chemical | 2,4-di-tert-butylphenol |
|  | Chemical | 2-naphthol sulfate |
|  | Chemical | 3,5-dichloro-2,6-dihydroxybenzoic acid |
|  | Chemical | 3-bromo-5-chloro-2,6-dihydroxybenzoic acid* |
|  | Chemical | 3-hydroxy-2-methylpyridine sulfate |
|  | Chemical | 3-hydroxypyridine sulfate |
|  | Chemical | 4-hydroxychlorothalonil |
|  | Chemical | 6-hydroxyindole sulfate |
|  | Chemical | dimethyl sulfone |
|  | Chemical | ectoine |
|  | Chemical | O-sulfo-L-tyrosine |
|  | Chemical | perfluorooctanesulfonate (PFOS) |
|  | Chemical | perfluorooctanoate (PFOA) |
|  | Chemical | sulfate* |
|  | Chemical | thioproline |
|  | Drug - Topical Agents | 2,6-dihydroxybenzoic acid |
|  | Drug - Topical Agents | hydroquinone sulfate |
|  | Drug - Topical Agents | salicylate |
|  | Food Component/Plant | (2,4 or 2,5)-dimethylphenol sulfate |
|  | Food Component/Plant | 1,6-anhydroglucose |
|  | Food Component/Plant | 2,3-dihydroxyisovalerate |
|  | Food Component/Plant | 2,3-dihydroxypyridine |
|  | Food Component/Plant | 2-acetamidophenol sulfate |
|  | Food Component/Plant | 2-aminophenol sulfate |
|  | Food Component/Plant | 2-isopropylmalate |
|  | Food Component/Plant | 2-keto-3-deoxy-gluconate |
|  | Food Component/Plant | 2-oxindole-3-acetate |
|  | Food Component/Plant | 2-piperidinone |
|  | Food Component/Plant | 3-ethylcatechol sulfate (1) |
|  | Food Component/Plant | 3-formylindole |
|  | Food Component/Plant | 3-hydroxystachydrine* |
|  | Food Component/Plant | 3-indoleglyoxylic acid |
|  | Food Component/Plant | 4-acetylcatechol sulfate (1) |
|  | Food Component/Plant | 4-allylphenol sulfate |
|  | Food Component/Plant | acesulfame |
|  | Food Component/Plant | alliin |
|  | Food Component/Plant | beta-guanidinopropanoate |
|  | Food Component/Plant | caffeic acid sulfate |
|  | Food Component/Plant | cinnamoylglycine |
|  | Food Component/Plant | dihydrocaffeate sulfate (2) |
|  | Food Component/Plant | ergothioneine |
|  | Food Component/Plant | erythritol |
|  | Food Component/Plant | ethyl alpha-glucopyranoside |
|  | Food Component/Plant | ethyl beta-glucopyranoside |
|  | Food Component/Plant | eugenol sulfate |
|  | Food Component/Plant | ferulic acid 4-sulfate |
|  | Food Component/Plant | gluconate |
|  | Food Component/Plant | glucuronide of piperine metabolite C17H21NO3 (3)* |
|  | Food Component/Plant | glucuronide of piperine metabolite C17H21NO3 (4)* |
|  | Food Component/Plant | glucuronide of piperine metabolite C17H21NO3 (5)* |
|  | Food Component/Plant | histidine betaine (hercynine)* |
|  | Food Component/Plant | homostachydrine* |
|  | Food Component/Plant | indolin-2-one |
|  | Food Component/Plant | maltol sulfate |
|  | Food Component/Plant | mannonate* |
|  | Food Component/Plant | methyl glucopyranoside (alpha + beta) |
|  | Food Component/Plant | methyl indole-3-acetate |
|  | Food Component/Plant | N-(2-furoyl)glycine |
|  | Food Component/Plant | N-acetylalliin |
|  | Food Component/Plant | piperine |
|  | Food Component/Plant | pyrraline |
|  | Food Component/Plant | quinate |
|  | Food Component/Plant | saccharin |
|  | Food Component/Plant | S-allylcysteine |
|  | Food Component/Plant | stachydrine |
|  | Food Component/Plant | sulfate of piperine metabolite C16H19NO3 (2)* |
|  | Food Component/Plant | sulfate of piperine metabolite C16H19NO3 (3)* |
|  | Food Component/Plant | sulfate of piperine metabolite C18H21NO3 (1)* |
|  | Food Component/Plant | sulfate of piperine metabolite C18H21NO3 (3)* |
|  | Food Component/Plant | tartarate |
|  | Food Component/Plant | tartronate (hydroxymalonate) |
|  | Food Component/Plant | thymol sulfate |
|  | Food Component/Plant | trans-3,4-methyleneheptanoate |
|  | Food Component/Plant | umbelliferone sulfate |
|  | Food Component/Plant | vanillic acid glycine |
|  | Tobacco Metabolite | cotinine |
|  | Xanthine Metabolism | 1,3-dimethylurate |
|  | Xanthine Metabolism | 1,7-dimethylurate |
|  | Xanthine Metabolism | 1-methylurate |
|  | Xanthine Metabolism | 1-methylxanthine |
|  | Xanthine Metabolism | 3,7-dimethylurate |
|  | Xanthine Metabolism | 3-methylxanthine |
|  | Xanthine Metabolism | 5-acetylamino-6-amino-3-methyluracil |
|  | Xanthine Metabolism | 5-acetylamino-6-formylamino-3-methyluracil |
|  | Xanthine Metabolism | 7-methylxanthine |
|  | Xanthine Metabolism | caffeine |
|  | Xanthine Metabolism | paraxanthine |
|  | Xanthine Metabolism | theobromine |
|  | Xanthine Metabolism | theophylline |

# Table S2. Results from univariate analysis (P<0.05, Q<0.05*)

| **Sub Pathway** | **Biochemical Name** | **Fold-Change**  **(FC)**  **T2/T1†** | **Log2FC†** | **P-value** | **Q-value** | ± Correlated metabolite *changes*, Pearson r>\|.80\|  ± Correlated metabolite *changes*, Pearson r >\|.50\| AND significant in partial correlation | **Description^7,8^ and/or potential connection to football participation** |
| --- | --- | --- | --- | --- | --- | --- | --- |
| Alanine and Aspartate Metabolism | N-acetylalanine | **1.11** | **0.15** | 0.008 | 0.033 | + cis-4-decanoylcarnitine (C10:1) | N-terminal acetylation of amino acids potentially functions in protein stability, localization, metabolism, apoptosis and synthesis. Mechanisms for acetylation of free amino acids are unknown; free acetylated amino acids may be a product of breakdown of acetylated residues in protein.  Blood levels ↑ after intense, long-duration training ^9,10^. |
| Creatine Metabolism | creatine | **0.80** | **-0.32** | 1.00E-03 | 0.010 |  | Often used as a supplement to enhance anaerobic exercise performance^11^. Also synthesized from arginine (NS in the current study), producing ornithine (↓ in the current study) in the process. Creatinine (NS in the current study), derived from creatine, is a marker of muscle metabolism and muscle mass^12^.  Blood levels ↓ after high intensive training ^10,13^.  Athletes have higher urinary levels vs non-athletes ^14^. Athletes in high cardiovascular demand (CD) activity have higher blood levels vs athletes in low/moderate CD activity^15^.  Concussed athletes have altered (not-specified) blood levels vs non-concussed athletes^16^. |
| Glutamate Metabolism | citramalate | **0.76** | **-0.40** | 0.002 | 0.016 |  | Also considered an hydroxy fatty acid. |
| Glutathione Metabolism | cysteinylglycine | **0.80** | **-0.32** | 0.008 | 0.033 |  | Derived from breakdown of glutathione (not assayed in current study).  Blood levels ↑ after strenuous exercise ^17^. Blood levels ↓ after intense military training^10^. |
|  | cysteine-glutathione disulfide | **0.82** | **-0.29** | 0.002 | 0.015 |  | Formed upon of oxidative stress of glutathione. |
| Glycine, Serine and Threonine Metabolism | glycine | **1.08** | **0.11** | 0.003 | 0.018 | + pipecolate | Urinary levels ↑ 24-72 h after eccentric exercise-induced muscle damage ^18^.  Blood and urine levels tend to ↓ after exercise ^19-21^. |
| Guanidino and Acetamido Metabolism | guanidinosuccinate | **0.86** | **-0.22** | 0.002 | 0.014 | + phenylacetylglutamine | Most often studied in the context of kidney dysfunction.  Blood levels ↓ ^13^ or ↑ ^10^ after intensive training.  Patients with severe TBI have lower blood levels vs healthy controls ^22^. |
| Histidine Metabolism | formiminoglutamate | **0.74** | **-0.43** | 4.00E-04 | 0.005 | + X-23665, hydantoin-5-proprionate, 2-aminoadipate, gamma-glutamyl-alpha-lysine. | Glutamic acid derivative; an intermediate in metabolism of histidine to glutamate. |
|  | hydantoin-5-propionate | **0.79** | **-0.34** | 0.003 | 0.020 | + formiminoglutamate | An intermediate in metabolism of histidine to glutamate.  Blood levels ↑ after intense military training^10^.  Athletes have higher urinary levels vs non-athletes ^14^. |
|  | imidazole lactate | **0.87** | **-0.20** | 0.004 | 0.022 | - X-18779 | An end-product of histidine metabolism.  Athletes in high CD activity have higher blood levels vs athletes in low/moderate CD activity ^15^. Athletes in high-power activity have higher levels vs athletes in moderate power activity ^23^. |
|  | histidine | **0.91** | **-0.14** | 0.003 | 0.017 | + gamma-glutamylhistidine | Multiple functions; low levels linked to inflammation and oxidative stress.  Urinary levels ↑ 48 h after eccentric exercise-induced muscle damage ^18^.  Blood levels ↓ after intense, long duration training (marathon, military)^10,24^. |
|  | 1-methyl-4-imidazoleacetate | **1.15** | **0.20** | 0.016 | 0.048 |  | Main end-product of histamine metabolism. Indicator of histamine turnover; typically expressed as a concentration of creatinine (NS in the current study). |
|  | N-acetyl-1-methylhistidine* | **1.36** | **0.44** | 0.013 | 0.043 |  | An acetylated derivative of 1-methylhistidine (NS in the current study) and a very strong basic compound.  Blood levels ↓ after intense military training^10^. |
| Leucine, Isoleucine and Valine Metabolism | 1-carboxyethylvaline | **0.63** | **-0.67** | 1.47E-05 | 5.36E-04 | RF-predictor (Fig S5) | Carboxyethyl-modified amino acids are poorly characterized. The parent amino acids of these derivatives did not significantly change in the current study. |
|  | 1-carboxyethylleucine | **0.66** | **-0.60** | 1.00E-04 | 0.002 | + 1-carboxyethylisoleucine. |  |
|  | 1-carboxyethylisoleucine | **0.73** | **-0.45** | 1.00E-03 | 0.010 | + 1-carboxyethylphenylalanine |  |
| Lysine Metabolism | pipecolate | **0.76** | **-0.40** | 9.00E-04 | 0.009 | + glycine | Lysine metabolite. Might originate from food or gut microbiota metabolism, but unclear.  Blood levels ↓ after intense military training^10^. |
|  | 2-aminoadipate | **0.82** | **-0.29** | 0.003 | 0.019 | + gamma-glutamyl-alpha-lysine, formiminoglutamate | Lysine metabolite. *Elevated* levels potentially signify lysine breakdown via oxidative stress.  Blood levels ↑ after exercise ^19^ and intense, long duration training but ↓ in 14 h recovery period^9,21^.  Blood levels of its metabolite, 2-oxoadipate (not assayed in current study), ↓ after an 80 day exercise program in male soldiers^25^ |
|  | lysine | **0.87** | **-0.20** | 0.003 | 0.017 | + gamma-glutamyl-alpha-lysine | An essential amino acid highly concentrated in muscle compared with other amino acids.  Blood levels ↓ after resistance training and intense, long duration training^10,21,24,26^.  In high-fit subjects, blood levels ↑ after endurance exercise then ↓ in recovery period^27^. |
|  | N6,N6,N6-trimethyllysine | **1.45** | **0.54** | 0.005 | 0.024 |  | Methylated derivative of lysine. It is a component of histone proteins, a precursor of carnitine and a coenzyme of fatty acid oxidation.  Blood levels ↓ after intense military training^10^.  Athletes in high CD activity have lower blood levels vs athletes in low/moderate CD activity ^15^. |
| Methionine, Cysteine, SAM and Taurine Metabolism | cysteine | **0.90** | **-0.15** | 0.004 | 0.022 | + X-14056, fibrinopeptide A (5-16)* | Naturally occurring sulfur-containing amino acid. Contains a thiol group which can undergo redox reactions; has antioxidant properties.  Blood levels ↓ at peak exercise time point ^24^.  Athletes in high CD activity have higher blood levels vs athletes in low/moderate CD activity ^15^. |
| Phenylalanine Metabolism | 1-carboxyethylphenylalanine | **0.62** | **-0.69** | 3.52E-05 | 9.30E-04 | + 1-carboxyethylisoleucine, 1-carboxyethyltyrosine | Carboxyethyl-modified amino acids are poorly characterized. The parent amino acid, phenylalanine, did not significantly change in the current study. |
|  | phenyllactate (PLA) | **0.76** | **-0.40** | 0.002 | 0.015 |  | Phenylalanine→phenylpyruvate→PLA  Phenylpyruvate nominally (P<0.05, Q>0.05) decreased in the current study.  Blood levels ↓ after intense military training^10^.  Weak positive association with % lean body mass^28^. Athletes in high-power activity have higher levels vs athletes in moderate power activity ^23^. |
| Polyamine Metabolism | 5-methylthioadenosine (MTA) | **0.89** | **-0.17** | 0.008 | 0.032 | + propionylcarnitine (C3) | Product of polyamine metabolism that can be used in methionine synthesis via the methionine salvage pathway. Methionine was not assayed in the current study. |
|  | (N(1) + N(8))-acetylspermidine | **1.28** | **0.36** | 7.25E-05 | 1.47E-03 | +androstenediol (3alpha, 17alpha) monosulfate (2) | N-acetylspermidine ↑ post 60 min endurance trial among elite cyclists^29^ |
|  | acisoga | **1.43** | **0.52** | 0.006 | 0.028 |  | Formed from N1-acetylspermidine.  Blood levels ↑ after endurance exercise ^23^. |
| Tryptophan Metabolism | N-acetyltryptophan | **1.46** | **0.55** | 0.016 | 0.049 |  | Catabolite of tryptophan (NS in the current study) converted by the gut microbiota. N-terminal acetylation of amino acids potentially functions in protein stability, localization, metabolism, apoptosis and synthesis.  Blood levels ↓ after intense military training^10^. |
|  | anthranilate | **1.71** | **0.77** | 0.015 | 0.047 | + X-21286 | Participates in several enzymatic reactions. In the tryptophan metabolism pathway, it is a metabolite of kynurenine and indoleacetate metabolism; neither of these compounds significantly changed in the current study.  Blood levels ↑ after marathon^24^ |
| Tyrosine Metabolism | 1-carboxyethyltyrosine | **0.67** | **-0.58** | 3.82E-05 | 9.85E-04 | + 1-carboxyethylphenylalanine | Carboxyethyl-modified amino acids are poorly characterized. The parent amino acid, tyrosine, did not significantly change in the current study. |
|  | 4-hydroxyphenylpyruvate | **0.73** | **-0.45** | 3.00E-04 | 0.004 |  | Metabolite of tyrosine via tyrosine aminotransferase.  Blood levels ↑ after intense, long duration running but ↓ in 14 h recover period ^9,21^.  Athletes in high CD activity have higher blood levels vs athletes in low/moderate CD activity ^15^. |
|  | 3-methoxytyramine sulfate | **0.77** | **-0.38** | 0.007 | 0.029 |  | A sulfated methoxyphenol; a metabolite of dopamine.  Well-trained subjects have higher urinary levels vs minimally trained subjects^30^ |
|  | vanillylmandelate (VMA) | **0.83** | **-0.27** | 0.005 | 0.023 |  | A methoxyphenol; a metabolite of epinephrine and norepinephrine.  High-endurance athletes have higher blood levels vs moderate-endurance athletes ^23^ |
|  | dopamine 3-O-sulfate | **0.92** | **-0.12** | 0.011 | 0.039 |  | In human blood dopamine exists predominantly in the sulfated form.  Free and sulfated catecholamines tend to ↑ with acute exercise onset^30,31^. |
|  | p-cresol glucuronide* | **3.80** | **1.93** | 0.016 | 0.049 |  | P-Cresol (the precursor) is mainly generated as an end product of tyrosine biotransformation by anaerobic intestinal bacteria. |
| Urea cycle; Arginine and Proline Metabolism | N-acetylproline | **0.77** | **-0.38** | 0.004 | 0.022 |  | N-terminal acetylation of amino acids potentially functions in protein stability, localization, metabolism, apoptosis and synthesis.  Blood levels ↓ after intense military training^10^. |
|  | proline | **0.86** | **-0.22** | 0.002 | 0.017 | + ornithine | A non-essential, proteinogenic amino acid synthesized from glutamate (NS in the current study).  Blood levels generally ↑ with acute exercise ^32^ and decrease in recovery phase ^19^ but effect may attenuate with improved fitness ^27^.  Blood levels ↓ after intense, long duration training (running, military)^10,24^.  Blood levels negatively correlate with TBI severity ^33,34^. Concussed athletes have altered (not-specified) blood levels vs non-concussed athletes^16^. |
|  | ornithine | **0.90** | **-0.15** | 0.009 | 0.034 | + proline | Synthesized from arginine (NS in the current study). May attenuate physical fatigue^35,36^. Frequently marketed to bodybuilders/weightlifters.  Blood levels ↓ after resistance or intense, long duration training (running, military)^10,24,26^. Also ↓ after an 80-day exercise program (male soldiers)^37^.  Blood levels negatively correlate with TBI severity^33^. |
|  | N,N,N-trimethyl-alanylproline betaine (TMAP) | **1.27** | **0.34** | 2.00E-04 | 0.003 |  | Elevated levels with reduced kidney function^38^. |
|  | pro-hydroxy-pro | **1.56** | **0.64** | 3.09E-06 | 2.13E-04 |  | Prolylhydroxyproline is a marker of bone collagen degradation, showing high sensitivity for the diagnosis of osteoporosis. |
| Fructose, Mannose and Galactose Metabolism | fructose | **0.57** | **-0.81** | 3.24E-06 | 2.17E-04 | ML predictor (Table S3) | Glucose is the preferred fuel in glycolysis. Fructose, galactose and mannose are monosaccharides that can also undergo glycolysis after phosphorylation. The preferred use of each for glycolysis may have changed over the course of the season.  High-endurance athletes have higher blood levels vs moderate-endurance athletes ^23^ |
|  | galactonate | **0.84** | **-0.25** | 0.007 | 0.030 |  | Conjugated galactonic acid, a metabolite of galactose (derived from lactose) that may enter may enter the pentose phosphate pathway.  Blood levels ↑ after marathon^39^. |
|  | mannose | **1.83** | **0.87** | 4.72E-06 | 2.85E-04 | RF/ML predictor (Table S3, Fig S5) | An essential hexose for glycoprotein synthesis.  Blood levels ↓ after high intensive training ^13^. Blood levels ↑ after marathon, normalized 24-48 h recovery ^20,40^ |
| Glycolysis, Gluconeogenesis, and Pyruvate Metabolism | glucose | **1.10** | **0.14** | 0.019 | 0.053 | Not Significant (Q>0.05) | During high intensity exercise, the metabolism of carbohydrate reserves (blood glucose and liver and muscle glycogen) provides the predominant fuel for muscle contraction. Thus, reliance on carbohydrates as an energy source decreases with the fitness level of the individual.  Blood levels ↑ immediately after endurance exercise^21^. |
|  | glycerate | **0.91** | **-0.14** | 0.031 | 0.072 | Not Significant (Q>0.05) |  |
|  | pyruvate | **0.80** | **-0.32** | 0.002 | 0.015 | + X-25343 | By-product of anaerobic glycolysis. During high-intensity exercise, ATP generation demands increase, and glucose becomes the predominant muscle substrate for ATP generation. Increased glycolysis leads to pyruvate formation.  Blood and urine levels ↑ up to 3 h after endurance/resistance exercise^21,29^ but generally ↓ in recovery period^19^.  Blood levels ↑ after marathon^20,24,39^. Blood levels ↓ after intense military training^10^. |
|  | lactate | **0.88** | **-0.18** | 0.002 | 0.015 |  | By-product of anaerobic glycolysis; formed from pyruvate^41^. Lactate inhibits lipolysis ^41,42^  Blood and urine levels ↑ up to 3 h after endurance/resistance exercise^19,21,24,26,29^ and generally ↓ in recovery period^19^.  Blood levels ↑ after marathon^24^.  Blood levels ↓ after intense military training^10^. |
| Tocopherol Metabolism | gamma-tocopherol/beta-tocopherol  [beta-tocopherol represents a very  small fraction of total tocopherols  and co-elutes with gamma] | **1.50** | **0.58** | 0.003 | 0.017 |  | Dietary supplements of vitamin E typically provide only alpha-tocopherol (NS in the current study) and thus supplementation is unlikely contributing to the increase observed. Gamma-, but not alpha-, tocopherol levels may increase in response to inflammation ^43,44^.  Blood levels ↓ after exercise and in recovery period among high-fit subjects^27^ |
| Oxidative Phosphorylation | phosphate | **1.27** | **0.34** | 0.004 | 0.021 |  | High amounts of phosphate are present in processed, fast food. Levels reportedly increase in response to intense anaerobic and aerobic exercise^45,46^, and partly relates to phosphate efflux from the intracellular stores in the muscle to the blood^46^. Endurance athletes have been reported to have elevated resting levels that may represent a metabolic training effect^46^.  Blood levels ↓ after exercise but ↑ in recovery period among high-fit subjects^27^. Blood levels ↑ after intense military training^10^. |
| TCA Cycle | aconitate [cis or trans] | **0.85** | **-0.23** | 0.004 | 0.022 |  | TCA cycle is a key source of energy and uses carbons supplied from glucose through the glycolysis pathway as well as amino acids that can enter into the cycle at multiple points.  Blood levels ↑ up to 3 h after endurance exercise^10,21,24,39^. |
|  | fumarate | **0.89** | **-0.17** | 0.009 | 0.035 |  | Blood and urine levels ↑ after exercise ^21,24,29^.  Blood levels ↑ after intense, long duration training (running, military)^9,10,20,24,40^. |
|  | citrate | **0.91** | **-0.14** | 0.013 | 0.044 |  | Blood levels ↑ after moderate/high intensive training and long duration training ^10,13,19-21,27,40^.  Urine levels ↓ after exercise^21^.  Urinary levels ↑ up to 48 h after eccentric exercise-induced muscle damage ^18^.  High-endurance athletes have higher blood levels vs moderate-endurance athletes ^23^  Blunt-trauma patients have lower blood levels vs healthy controls ^47^. |
| Androgenic Steroids | androstenediol (3beta,17beta) disulfate (1) | **1.26** | **0.33** | 0.008 | 0.032 | + 2-hydroxysebacate | Resistance exercise elicits an acute hormonal response^48^. Testosterone (not measured) is stimulated by DHEA-S (NS in the current study), the predominant adrenal steroid and reflects long-term adrenal function. Other testosterone precursors assayed in the current study did not change. Changes in their sulphated metabolites may be delayed indicators of acute hormonal response to previous exercise.  With the exception of “androstenediol (3alpha, 17alpha) monosulfate (2)” (which decreased), these metabolites also ↑ after intense military training^10^. |
|  | 5alpha-androstan-3alpha,17beta-diol monosulfate (1) | **1.29** | **0.37** | 0.010 | 0.038 |  |  |
|  | androstenediol (3beta,17beta) monosulfate (1) | **1.30** | **0.38** | 0.007 | 0.030 | + 5alpha-androstan-3alpha,17beta-diol monosulfate (1)  - cytosine |  |
|  | androstenediol (3alpha, 17alpha) monosulfate (2) | **1.41** | **0.50** | 1.47E-05 | 5.36E-04 | + urate, cortisol, (N(1) + N(8))-acetylspermindine |  |
|  | 5alpha-androstan-3beta,17beta-diol monosulfate (2) | **1.59** | **0.67** | 0.009 | 0.034 | + X-22834, caffeic acid sulfate |  |
| Ceramides | N-palmitoyl-sphingosine (d18:1/16:0) | **0.89** | **-0.17** | 0.002 | 0.016 | - X-17146. | Ceramides are one of the hydrolysis byproducts of sphingomyelin and have strong biological activity and  play a central role in cell membrane integrity, cellular stress  response, inflammatory signaling, and apoptosis. None of the 29 sphingomyelins assayed in the current study changed. |
| Corticosteroids | cortolone glucuronide (1) | **0.82** | **-0.29** | 0.004 | 0.021 |  | Natural metabolite of cortolone, a downstream metabolite of cortisone. Reduced levels may be a consequence of elevated cortisone (P<0.05, Q>0.05 in the current study) & cortisol rather than impaired cortisone/cortisol metabolism. |
|  | cortisol | **1.77** | **0.82** | 5.49E-07 | 6.63E-05 | + androstenediol (3alpha, 17alpha) monosulfate (2) , 17alpha-hydroxypregnenolone 3-sulfate  RF/ML-predictor (Table S3, Fig S5) | Catabolic hormone involved in lipolysis, glycogenolysis, and proteolysis to mobilize fatty acids, glucose, and amino acids for energy purposes^42^. Generally catabolic effects of cortisol can be counteracted by anabolic hormones^42^. Protocols high in total work volume, moderate to high intensity with short rest periods elicit greater acute elevations in cortisol than conventional strength/power training^48^. Resting levels generally reflect a long-term training stress but no consistent patterns have emerged with different training protocols^48^.  Blood levels ↑ after intense, long duration running ^9^.  High-endurance athletes have higher blood levels vs moderate-endurance athletes ^23^.  Athletes in high CD activity have higher blood levels vs athletes in low/moderate CD activity ^15^.  Blood levels ↓ after head injury (via hypothalamic-pituitary-adrenal (HPA) axis damage) ^49^. |
|  | corticosterone | **3.34** | **1.74** | 1.87E-05 | 5.88E-04 | Pre: detectable in 13% of samples  Post: 65%  + 3-hydroxy-3-methylglutarate | Steroid lipid.  Blood levels ↑ after intense, long duration running and 14 h recovery period ^9^. |
| Diacylglycerol | palmitoleoyl-arachidonoyl-glycerol (16:1/20:4) [2]* | **0.73** | **-0.45** | 7.00E-04 | 0.008 | - 1-stearoyl-2-docosahexaenoyl-GPE (18:0/22:6)* | A diacylglycerol (DAG) is a glyceride consisting of two fatty acid chains covalently bonded to a glycerol (↑ in the current study) molecule through ester linkages. Synthesis begins with glycerol-3 phosphate (NS in the current study), which is derived primarily from dihydroxyacetone phosphate (not assayed), a product of glycolysis. DAGs are also derived from phospholipid metabolism. They are also common food additives.  With the exception of “diacylglycerol (14:0/18:1, 16:0/16:1) [2]*” and “stearoyl-arachidonoyl-glycerol (18:0/20:4) [2]*”, the same diacylglycerols reportedly ↓ in the blood after intense military training^10^.  High-endurance athletes also have lower blood levels of “palmitoleoyl-arachidonoyl-glycerol (16:1/20:4) [2]*” and “palmitoleoyl-linoleoyl-glycerol (16:1/18:2) [1]*” vs moderate-endurance athletes ^23^ |
|  | oleoyl-oleoyl-glycerol (18:1/18:1) [1]* | **0.79** | **-0.34** | 0.004 | 0.021 |  |  |
|  | palmitoleoyl-linoleoyl-glycerol (16:1/18:2) [1]* | **0.81** | **-0.30** | 0.006 | 0.028 | + in diacylglycerol (16:1/18:2 [2], 16:0/18:3 [1])* |  |
|  | diacylglycerol (14:0/18:1, 16:0/16:1) [2]* | **0.81** | **-0.30** | 0.012 | 0.040 |  |  |
|  | diacylglycerol (16:1/18:2 [2], 16:0/18:3 [1])* | **0.82** | **-0.29** | 0.008 | 0.032 | + palmitoleoyl-linoleoyl-glycerol (16:1/18:2) [1]* |  |
|  | oleoyl-linoleoyl-glycerol (18:1/18:2) [1] | **0.84** | **-0.25** | 0.007 | 0.031 | + oleoyl-linoleoyl-glycerol (18:1/18:2) [2] |  |
|  | oleoyl-linoleoyl-glycerol (18:1/18:2) [2] | **0.84** | **-0.25** | 0.008 | 0.033 | + oleoyl-linoleoyl-glycerol (18:1/18:2) [1] and maltol sulfate. |  |
|  | stearoyl-arachidonoyl-glycerol (18:0/20:4) [2]* | **0.84** | **-0.25** | 0.011 | 0.039 |  |  |
| Endocannabinoid | N-palmitoylserine | **0.75** | **-0.42** | 5.00E-04 | 0.006 |  | Blood levels of certain endocannabinoids decrease in the presence of chronic stress possibly contributing to stress adaptation ^50^. N-acylethanolamines, endocannabinoid-like molecules, reportedly increase in specific cerebral areas as protective response to neural damage in animal models^51^. |
| Fatty Acid Metabolism (Acyl Carnitine, Monounsaturated) | undecenoylcarnitine (C11:1) | **0.71** | **-0.49** | 4.00E-04 | 0.005 |  | Medium-chain acyl fatty acid derivative ester of carnitine. Not well characterized. Metabolite of incomplete fatty acid beta-oxidation. |
|  | cis-4-decenoylcarnitine (C10:1) | **1.35** | **0.43** | 0.014 | 0.045 | + N-acetylalanine, linolenoylcarnitine (C18:3)* | Medium-chain acyl fatty acid derivative ester of carnitine. Not well characterized. Metabolite of incomplete fatty acid beta-oxidation.  Blood levels ↑ up to 24 h after moderate/intense, medium/long duration training ^9,21^. |
| Fatty Acid Metabolism (Acyl Carnitine, Polyunsaturated) | linolenoylcarnitine (C18:3)* | **1.35** | **0.43** | 0.016 | 0.049 | + cis-4-decenoylcarnitine (C10:1) | Long-chain acyl fatty acid derivative ester of carnitine. Energy production from LCFAs requires the transport of LCFAs into the mitochondrial matrix. This transport is carnitine-dependent and involves translocation machinery. Also a metabolite of incomplete fatty acid beta-oxidation.  Blood levels ↑ after intense military training^10^. |
| Fatty Acid Metabolism (Acyl Choline) | oleoylcholine | **0.87** | **-0.20** | 0.021 | 0.057 | Not Significant (Q>0.05) | Not well characterized. See Discussion |
|  | stearoylcholine* | **0.86** | **-0.22** | 0.022 | 0.058 | Not Significant (Q>0.05) |  |
|  | palmitoylcholine | **0.88** | **-0.18** | 0.025 | 0.063 | Not Significant (Q>0.05) |  |
|  | palmitoloelycholine | **0.89** | **-0.17** | 0.033 | 0.075 | Not Significant (Q>0.05) |  |
|  | dihomo-linolenoyl-choline | **0.80** | **-0.32** | 0.004 | 0.021 | + docosahexaenoylcholine, arachidonoylcholine. |  |
|  | docosahexaenoylcholine | **0.84** | **-0.25** | 0.012 | 0.042 | + dihomo-linolenoyl-choline, arachidonoylcholine |  |
|  | arachidonoylcholine | **0.85** | **-0.23** | 0.014 | 0.044 | + docosahexaenoylcholine, dihomo-linolenoyl-choline. |  |
| Fatty Acid Metabolism (Acyl Glycine) | 3-hydroxybutyroylglycine** | **3.35** | **1.74** | 0.011 | 0.040 | + glutamine conjugate of C6H10O2 (1)* | Not well characterized. |
| Fatty Acid Metabolism (also BCAA Metabolism) | propionylcarnitine (C3) | **0.86** | **-0.22** | 0.015 | 0.048 | + 5-methlythioadenosine (MTA) | Acylcarnitine, generally a marker of incomplete fatty acid beta-oxidation.  Blood levels ↓ after intense military training^10^.  Blood levels ↑ after intense, long duration running but ↓ in 14 h recover period ^9,21^.  Blood levels negatively correlate with TBI severity^52^. |
| Fatty Acid, Dicarboxylate | tridecenedioate (C13:1-DC)* | **0.60** | **-0.74** | 1.05E-05 | 4.59E-04 | + heptenedioate (C7:1-DC)*, 10-undecenoate (11:1n1) | Medium-chain (C6-C12) dicarboxylic acids are produced by animals via fatty acid ω-oxidation (or by β-oxidation of longer-chain dicarboxylic acids). The ω -oxidative pathway is prominent in brain and could play a role in brain fatty acid metabolism^53^. Recently regarded as possible alternate fuel substrates in both normal and pathological conditions in man, since they are rapidly beta-oxidized in both mitochondria and peroxisomes^53^. Even-numbered, medium-chain dicarboxylic acids, through their end product, succinyl-CoA,  produce oxaloacetate and thus ultimately glucose. The process has an amino acid sparing effect in the gluconeogenic pathway.  In the current study medium chained unsaturated dicarboxylic acids generally decreased while their saturated counterparts increased. The latter is generally consistent with metabolomic studies of samples collected after intense, long duration training (running, military) ^9,10^ and acute exercise ^19^.  Saturated dicarboxylic acids may derive partially from metabolic degradation of unsaturated dicarboxylic acids ^54^; requiring additional enzymes and NADPH. |
|  | heptenedioate (C7:1-DC)* | **0.72** | **-0.47** | 5.00E-04 | 0.006 | + tridecenedioate (C13:1-DC)* |  |
|  | 2-hydroxysebacate | **0.80** | **-0.32** | 0.005 | 0.024 | + androstenediol (3beta, 17beta) disulfate (1) |  |
|  | hexadecenedioate (C16:1-DC)* | **0.87** | **-0.20** | 0.008 | 0.033 |  |  |
|  | 2-hydroxyglutarate | **1.39** | **0.48** | 3.20E-08 | 9.66E-06 | + X-23786  ML/RF predictor (Table S3, Fig S5) |  |
|  | pimelate (C7-DC) | **1.44** | **0.53** | 0.007 | 0.031 |  |  |
|  | undecanedioate (C11-DC) | **1.57** | **0.65** | 7.09E-05 | 1.45E-03 | + X-25397, X-21829, X-25452, X-24928, 8-hydroxyoctanoate, azelate (C9-DC), glycylvaline, sebacate (C10-DC), suberate (C8-DC), pelargonate (9:0) |  |
|  | suberate (C8-DC) | **1.72** | **0.78** | 1.99E-05 | 6.01E-04 | + X-25452, X-25397, X-24928, azelate (C9-DC), sebacate (C10-DC), undecanedioate (C11-DC) |  |
|  | azelate (C9-DC) | **1.73** | **0.79** | 1.00E-04 | 0.002 | + undecanedioate (C11-DC), suberate (C8-DC), X-21829, X-25397, X-24928, X-25452, 8-hydroxyoctanoate, sebacate (C10-DC), pelargonate (9:0) |  |
|  | dodecadienoate (12:2)* | **1.74** | **0.80** | 0.009 | 0.036 | + stearidonate (18:4n3) |  |
|  | sebacate (C10-DC) | **1.81** | **0.86** | 2.81E-06 | 2.03E-04 | + undecanedioate (C11-DC), X-25452, X-25397, X-24928, X-21829, azelate (C9-DC), suberate (C8-DC), 8-hydroxyoctanoate, glycylvaline, pelargonate (9:0)  ML-Predictor (Table S3) |  |
| Fatty Acid, Dihydroxy | 3,4-dihydroxybutyrate | **1.33** | **0.41** | 5.00E-04 | 0.006 |  | Not well characterized. Biomarker of semialdehyde dehydrogenase (SSADH) deficiency, a genetic disorder.  Blood levels positively correlate with TBI severity^34^. |
| Fatty Acid, Monohydroxy | 8-hydroxyoctanoate | **1.59** | **0.67** | 2.93E-06 | 2.07E-04 | + X-25397, X-21829, X-24928, X-25452, undecanedioate (C11-DC), sebacate (C10-DC), azelate (C9-DC), pelargonate (9:0)  ML-predictor (Table S3) | Omega-hydroxy medium chain fatty acids; metabolites of oxidation of the medium chain fatty acid caprylate (↓ in current study) |
|  | 7-hydroxyoctanoate | **1.70** | **0.77** | 0.003 | 0.019 |  |  |
| Glycerolipid Metabolism | Glycerophosphoglycerol (GPG) | **0.68** | **-0.56** | 8.02E-05 | 0.002 |  | Head group of a class of glycerophospholipids in which a phosphoglycerol moiety occupies a glycerol substitution site. Three metabolites of this class were assayed but did not change in the current study. GPC, GPI, GPE levels also did not change. |
|  | glycerol | **1.59** | **0.67** | 0.016 | 0.049 |  | Component of triglycerides and diglycerides; sources of fatty acid.  The increase may suggest increased reliance on fatty acids as fuel and also increased phospholipid metabolism.  Blood levels ↑ after moderate or intense, long or short duration training (running, military)^9,10,20,21,24,40^.  Blood levels positively correlate with TBI severity^34^. |
| Long Chain Polyunsaturated Fatty Acid (n3 and n6) | linoleate (18:2n6) | **1.75** | **0.81** | 0.006 | 0.028 | + linolenate [alpha or gamma;(18:3n3 or 6)] | Conjugate base of a linoleic acid, an essential fatty acid that functions in biosynthesis of prostaglandins and cell membranes. It is the direct precursor to oxidized metabolites including 13- and 9-hydroxy-octadecadienoic acid (NS in the current study), stable oxidation products linked to pathological conditions.  Blood levels ↓ after acute exercise but ↑ in recovery period among high-fit subjects ^27,55^.  Blood levels ↑ after moderate/intense, medium/long duration training^9,10,20,21^.  Increased in TBI patients^56^ and animal models of TBI ^57^ |
|  | linolenate [alpha or gamma; (18:3n3 or 6)] | **1.99** | **0.99** | 1.00E-03 | 0.010 | + linoleate (18:2n6) | Conjugate of linolenic acid, an essential fatty acid shown to inhibit the synthesis of prostaglandin resulting in reduced inflammation and prevention of certain chronic diseases. Proposed benefits for TBI prevention and treatment^58,59^.  Blood levels ↑ after moderate/intense, moderate/long duration training^9,10,21,29,40^ & recovery period. |
|  | stearidonate (18:4n3) | **3.55** | **1.83** | 9.00E-04 | 0.009 | + dodecadienoate (12:2)* | First metabolite of α‐linolenic acid (↑ in current study) in the metabolic pathway leading to C20–22 fatty acids, such as eicosapentaenoic acid (EPA, 20:5n‐3; NS in the current study), and docosahexaenoic acid (DHA, 22:6n‐3; NS in the current study). Increased in animal models of TBI ^57^.  Blood levels ↑ after moderate/intense, medium/long duration training ^9,10,21^ & recovery period. |
| Lysophospholipid | 1-linoleoyl-GPI (18:2)* | **1.41** | **0.50** | 0.011 | 0.039 |  | Lysophospholipids (and free fatty acids) are products of phospholipid degradation. They usually reacylate to generate new phospholipids or are degraded further. Several other lysophosphatidylcholines and lysophosphatidylinositols increased nominally (P<0.05, Q>0.05); each had unsaturated fatty acid chains.  LysoGPEs generally decrease up to 24 h after exercise ^21^ but LysoGPEs did not change in the current study.  Blood levels of 1-linolenoyl-GPC (18:3) ↓ after intense military training^10^. Athletes in high-power activity have higher levels of 1-linolenoyl-GPC (18:3) vs athletes in moderate power activity ^23^. |
|  | 1-linolenoyl-GPC (18:3)* | **1.43** | **0.52** | 0.002 | 0.014 |  |  |
| Medium Chain Fatty Acid | caprate (10:0) (or decanoate) | **0.90** | **-0.15** | 0.019 | 0.054 | Not Significant (Q>0.05) | See Discussion.  Blood levels of caprylate,caprate,caproate, and 10-undecenoate ↑ after moderate/intense, medium/long duration training ^9,10,21^ and recovery period.  Blood levels of 10-undecenoate are higher in patients with severe (but not mild/moderate) TBI vs controls^34^. |
|  | caprylate (8:0) (or octanoate) | **0.81** | **-0.30** | 0.005 | 0.025 |  |  |
|  | 10-undecenoate (11:1n1) | **0.81** | **-0.30** | 0.006 | 0.027 | + tridecenedioate (C13:1-DC)* |  |
|  | caproate (6:0) (or hexanoate) | **0.83** | **-0.27** | 1.00E-04 | 0.002 | + taurochenodeoxycholate, taurocholate |  |
|  | heptanoate (7:0) | **0.83** | **-0.27** | 4.00E-04 | 0.005 | ML-predictor |  |
|  | pelargonate (9:0) | **0.86** | **-0.22** | 0.003 | 0.020 | + X-21829, X-16245, X-25397, X-15220, sebacate (C10-DC), 8-hydroxyoctanoate, X-23786, undecanedioate (C11-DC), X-25452, X-24928, X-25407, azelate (C9-DC) |  |
|  | undecanoate (11:0) | **0.92** | **-0.12** | 0.009 | 0.035 | +X-25172 |  |
| Mevalonate Metabolism | 3-hydroxy-3-methylglutarate (or meglutol) | **0.90** | **-0.15** | 0.013 | 0.043 | - corticosterone | “Off-product” intermediate of leucine degradation.  Blood levels ↑ after high and moderate intensive training ^10,13^. |
| Phosphatidylethanolamine (PE) | 1-stearoyl-2-arachidonoyl-GPE (18:0/20:4) | **0.79** | **-0.34** | 9.71E-06 | 4.41E-04 | + 1-palmitoyl-2-arachidonoyl-GPE (16:0/20:4)*  ML-predictor (Table S3) | See Discussion on phospholipids and this class of glycerophospholipids in particular. |
|  | 1-palmitoyl-2-arachidonoyl-GPE (16:0/20:4)* | **0.81** | **-0.30** | 8.00E-04 | 0.008 | + 1-stearoyl-2-arachidonoyl-GPE (18:0/20:4) | Athletes in high CD activity have higher blood levels vs athletes in low/moderate CD activity ^15^.  Blood levels are higher in athletes with mild/moderate TBI vs healthy athletes ^60^ |
|  | 1-stearoyl-2-docosahexaenoyl-GPE (18:0/22:6)* | **0.84** | **-0.25** | 0.007 | 0.030 | - palmitoleoyl-arachidonoyl-glycerol (16:1/20:4) [2]* |  |
|  | 1-palmitoyl-2-docosahexaenoyl-GPE (16:0/22:6)* | **0.85** | **-0.23** | 0.010 | 0.038 |  | Blood levels are lower in athletes with mild/moderate TBI vs healthy athletes ^60^ |
|  | 1-stearoyl-2-linoleoyl-GPE (18:0/18:2)* | **0.86** | **-0.22** | 0.015 | 0.048 |  | Blood levels ↓ after intense military training^10^. |
| Phospholipid Metabolism | phosphoethanolamine | **0.82** | **-0.29** | 9.00E-04 | 0.009 | + choline phosphate, 2-keto-3-deoxy-gluconate | Lipid moiety of phosphatidylethanolamines (↓ in current study). Also a precursor for the synthesis of N-acyl-phosphatidylethanolamine (thus, anandamide) and donor of ethanolamine phosphate for synthesis of GPI anchors that attach signaling proteins to the surface of plasma membrane.  Product of sphinganine-1-phosphate ((↓ in current study: P<0.05 Q>0.05) and sphinogosine-1-phosphate (NS in the current study) metabolism. |
|  | choline phosphate (or phosphocholine) | **0.85** | **-0.23** | 0.003 | 0.018 | + phosphoethanolamine | Substrate for synthesis of choline (↓ in current study: P<0.05 Q>0.05) and citicoline (not assayed). Downstream product of phosphoethanolamine (↓ in current study) metabolism. |
| Pregnenolone Steroids | 17alpha-hydroxypregnenolone 3-sulfate  (or 17-hydroxypregnenolone) | **1.85** | **0.89** | 6.00E-04 | 0.007 | + cortisol | Product of pregnenolone hydroxylation. Pregnenolone was not assayed but pregnenolone sulfate (P=0.04, Q>0.05) and 21-hydroxypregnenolone monosulfate (1) (P=0.05, Q>0.05) ,other metabolites of pregnenolone, nominally increased.  Also a prohormone in the formation of dehydroepiandrosterone (DHEA, NS in the current study). |
| Primary Bile Acid Metabolism | chenodeoxycholate | **2.78** | **1.48** | 0.028 | 0.067 | Not Significant (Q>0.05) | See Discussion.  Blood levels of primary bile acids generally ↓ up to 24 h after exercise ^61^ ^9,10,21,61^  Blood levels of taurochenodeoxycholate are lower in athletes with mild/moderate TBI vs healthy athletes ^60^. Taurochenodeoxycholate to be neuroprotective in humans through the prevention of apoptosis and other pathobiologic cascades in a variety of human neurological disorders, including TBI^62^  Athletes in high-power activity have higher levels of cholate vs athletes in moderate power activity ^23^. |
|  | glycochenodeoxycholate | **0.95** | **-0.07** | 0.040 | 0.084 | Not Significant (Q>0.05) |  |
|  | glycocholate | **0.96** | **-0.06** | 0.040 | 0.085 | Not Significant (Q>0.05) |  |
|  | taurocholate | **0.85** | **-0.23** | 0.009 | 0.035 | + taurochenodeoxycholate, taurodeoxycholate, caproate (8:0) |  |
|  | taurochenodeoxycholate | **0.97** | **-0.04** | 0.013 | 0.044 | + taurocholate, taurodeoxycholate, glycodeoxycholate, caproate (8:0) |  |
|  | cholic acid glucuronide | **1.48** | **0.57** | 0.003 | 0.017 |  |  |
|  | cholate | **6.86** | **2.78** | 1.00E-04 | 0.002 | + hyocholate |  |
| Secondary Bile Acid Metabolism | taurocholenate sulfate* | **0.81** | **-0.30** | 0.004 | 0.022 | - X-18779 | See Discussion. Bile acids are largely re-absorbed in the terminal ileum by an active transport mechanism, but less than 5% of the pool enters the colon per day. In the colon, bile acids are metabolized by bacterial flora to secondary bile acids.  Blood levels ↑ after intense, long duration training (running, military)^9,10^. |
|  | taurodeoxycholate | **0.86** | **-0.22** | 0.006 | 0.029 | + taurochenodeoxycholate, taurocholate, glycodeoxycholate  *deoxycholate was nominally increased (P=0.03, Q=0.07) | Blood levels ↓ after intense, long duration running and in 14 h recover period ^9,21^. Also ↓ after an 80-day exercise program (male soldiers)^37^. |
|  | glycodeoxycholate | **0.87** | **-0.20** | 0.011 | 0.040 | + taurodeoxycholate, taurochenodeoxycholate  *deoxycholate was nominally increased (P=0.03, Q=0.07) | Blood levels ↓ after intense, long duration running ^9,21^. |
|  | hyocholate | **1.97** | **0.98** | 0.002 | 0.013 | + cholate |  |
|  | ursodeoxycholate | **2.40** | **1.26** | 0.015 | 0.047 |  | Blood levels ↓ after endurance and intense military training^10,61^. |
| Sphingosines | hexadecasphingosine (d16:1)* | **0.87** | **-0.20** | 0.011 | 0.040 | - ethyl alpha-glucopyranoside. | Not well characterized. |
| Purine Metabolism, (Hypo)Xanthine/Inosine containing | urate | **1.10** | **0.14** | 0.004 | 0.020 | - androstenediol (3alpha, 17alpha) monosulfate (2) | Final oxidation product of purine metabolism. Endogenous antioxidant. Consistently shown to increase post-exercise.  Blood levels ↑ after exercise and recovery period, particularly in high-fit subjects ^24,27^.  Blood levels ↑ after intense military training^10^. |
| Purine Metabolism, Adenine containing | adenine | **0.93** | **-0.10** | 0.006 | 0.028 | - Fibrinopeptide B (1-11)** | Component of DNA and RNA. Ubiquitously occurring, thus modest changes are difficult to interpret. |
| Purine Metabolism, Guanine containing | 7-methylguanine | **1.07** | **0.10** | 0.011 | 0.039 | + o-cresol sulfate, (2,4 or 2,5)-dimethylphenol sulfate | Metabolite of DNA methylation and depurination. Possibly higher in smokers vs nonsmokers. |
| Pyrimidine Metabolism, Cytidine containing | cytosine | **0.78** | **-0.36** | 0.011 | 0.040 | - androstenediol (3beta,17beta) monosulfate (1) | Component of DNA and RNA. Ubiquitously occurring, thus modest changes are difficult to interpret.  Urinary levels ↑ with external training load in soccer players ^63^. |
|  | 3-methylcytidine | **0.90** | **-0.15** | 0.003 | 0.019 |  |  |
| Pyrimidine Metabolism, Thymine containing | 5,6-dihydrothymine | **1.23** | **0.30** | 0.002 | 0.013 |  | Intermediate breakdown product of thymine. |
| Pyrimidine Metabolism, Uracil containing | beta-alanine | **0.74** | **-0.43** | 7.00E-04 | 0.008 |  | A component of the naturally occurring peptides carnosine and anserine and also of pantothenic acid (vitamin B-5), which itself is a component of coenzyme A. Is metabolized into acetic acid but can undergo a transanimation reaction with pyruvate to form malonate-semialdehyde and L-alanine.  Popular nutrient supplement for physical performance enhancement; particularly exercise capacity ^64^.  Blood levels ↓ after intense military training^10^. |
|  | 5-methyluridine (ribothymidine) | **1.11** | **0.15** | 0.004 | 0.021 | - X-14056 | Endogenous methylated nucleoside.  Blood levels ↑ after intense military training^10^. |
| Partially Characterized Molecules | branched-chain, straight-chain, or cyclopropyl 10:1 fatty acid (2)* | **0.76** | **-0.40** | 7.00E-04 | 0.008 |  |  |
|  | glutamine conjugate of C6H10O2 (1)* | **2.57** | **1.36** | 0.002 | 0.017 | + 3-hydroxybutyroylglycine** |  |
| Acetylated Peptides | phenylacetylglutamine | **2.10** | **1.07** | 0.016 | 0.049 | + X-21286, guanidinosuccinate | Product formed from the conjugation of phenylacetate and glutamine. Likely a microbial metabolite^65^.  Blood levels ↑ after intense military training^10^. |
| Dipeptide | Glycylvaline | **2.53** | **1.34** | 0.014 | 0.045 | + azelate (C9-DC), undecanedioate (C11-DC), sebacate (C10-DC), X-24928, X-25452  - Fibrinopeptide A (3-16)** | Dipeptides are incomplete breakdown products of protein digestion or protein catabolism. Some dipeptides are known to have physiological or cell-signaling effects although most are simply short-lived intermediates on their way to specific amino acid degradation pathways following further proteolysis. |
| Fibrinogen Cleavage Peptide | Fibrinopeptide A (5-16)* | **0.85** | **-0.23** | 0.003 | 0.019 | + cysteine | Since thrombin cleaves fibrinopeptides A and B from the fibrinogen molecule, measurement of fibrinopeptide levels in plasma may provide a direct index of thrombin action. Fibrinogen, one of the most abundant proteins in blood, plays a key role in hemostasis, inflammation, wound healing, and additional physiological and pathological processes^66^.  The decrease in Fibrinopeptide A and increase in Fibrinopeptide B may suggest altered thrombin cleavage activity. |
|  | Fibrinopeptide A (3-16)** | **0.85** | **-0.23** | 0.007 | 0.030 | + glycylvaline |  |
|  | Fibrinopeptide B (1-11)** | **1.53** | **0.61** | 0.004 | 0.020 | - adenine |  |
| Gamma-glutamyl Amino Acid | gamma-glutamyl-alpha-lysine | **0.87** | **-0.20** | 0.012 | 0.041 | + lysine, 2-aminoadipate and formiminoglutamate. | Also considered dipeptides; incomplete breakdown products of protein digestion or protein catabolism. Some dipeptides are known to have physiological or cell-signaling effects although most are simply short-lived intermediates on their way to specific amino acid degradation pathways following further proteolysis.  Blood levels ↓ after intense military training^10^. |
|  | gamma-glutamylhistidine | **0.91** | **-0.14** | 0.007 | 0.029 | + histidine |  |
| Benzoate Metabolism | 3-methyl catechol sulfate (2) | **0.85** | **-0.23** | 0.016 | 0.048 | + o-cresol sulfate | Conjugate of sulfate and 3-methylcatechol; a bacterial metabolite.  Potential urinary biomarker of whole grain intake^67^.  Blood levels of benzoate metabolites are generally higher in football players vs other sport athletes^68^. |
|  | o-cresol sulfate | **2.64** | **1.40** | 1.79E-05 | 5.79E-04 | + (2,4 or 2,5)-dimethylphenol sulfate, 7-methylguanine, 3-methyl catechol sulfate (2) | Conjugate of sulfate and o-cresol, used commercially as a disinfectant but exists in all living species, ranging from bacteria to humans. Exposure may occur by inhalation, by cutaneous adsorption or by oral ingestion. Also present in beer. |
| Chemical | 2,2'-Methylenebis(6-tert-butyl-p-cresol) | **1.19** | **0.25** | 1.40E-03 | 0.012 |  | Present in industrial materials: paper, plastics, building materials. |
|  | O-sulfo-L-tyrosine | **1.35** | **0.43** | 1.46E-05 | 5.34E-04 | - X-17146 | Potential plasma biomarker of reduced kidney function^69,70^. |
|  | 1,2,3-benzenetriol sulfate (2) | **8.24** | **3.04** | 7.21E-05 | 1.46E-03 |  | Conjugate of sulfate and 1,2,3-benezenetriol (or pyrogallol, pyrogallic acid). A ubiquitous phenolic moiety found in flavonoids and polyphenols in a variety of edible plants, such as cacao, coffee, nuts, fruit peels, vegetables, and medicinal plants^71^.  Blood levels ↓ after intense military training^10^. |
| Food Component/Plant | maltol sulfate | **0.77** | **-0.38** | 0.002 | 0.015 | Pre: detected in 78% of samples.  Post: 30%  + oleoyl-linoleoyl-glycerol (18:1/18:2) [2] | Non-sulfated form is a sweet, baked and bread tasting compound present in or added to a variety of foods. |
|  | gluconate | **0.78** | **-0.36** | 3.00E-04 | 0.004 | RF-predictor (Fig S5) | Product of glucose metabolism (via pentose pathway). Occurs naturally in fruit, honey and wine and is used as a food additive. Also used in formulation of pharmaceuticals.  Blood levels ↓ after intense military training^10^. |
|  | 2-isopropylmalate | **0.82** | **-0.29** | 0.003 | 0.018 |  | Product of pyruvate metabolism. An intermediate metabolite in leucine biosynthesis. Also considered an hydroxy fatty acid; elevated during fasting and diabetic ketoacidosis |
|  | 2-keto-3-deoxy-gluconate  (2-dehydro-3-deoxy-D-galactonate) | **0.91** | **-0.14** | 0.009 | 0.035 | + X-25271 | Downstream product of galactose metabolism. Also referred to as a medium-chain keto acid derivative. |
|  | ferulic acid 4-sulfate | **1.10** | **0.14** | 0.007 | 0.030 |  | A phenolic acid present in a variety of plant foods.  Higher in footballers relative to other sport athletes^68^. |
|  | caffeic acid sulfate | **1.27** | **0.34** | 0.009 | 0.034 | - 5alpha-androstan-3beta,17beta-diol monosulfate (2) | An hydroxycinnamic acid present in a variety of plant foods.  Blood levels are higher in football players vs other sport athletes^68^. |
|  | (2,4 or 2,5)-dimethylphenol sulfate | **2.40** | **1.26** | 2.00E-04 | 0.003 | + o-cresol sulfate, 7-methylguanine | Non-sulfated forms are food additives. Have been identified in alcoholic beverages, coffee products. Previously shown to correlate with o-cresol ^72^ |
|  | 4-allylphenol sulfate | **2.71** | **1.44** | 0.013 | 0.044 |  | Not well characterized. |
|  | saccharin | **5.68** | **2.51** | 2.00E-04 | 0.003 | Pre: detected in 13% of samples  Post: 65% | Artificial sweetener in the diet. |
|  | ethyl alpha-glucopyranoside | **6.41** | **2.68** | 0.011 | 0.039 | - hexadecasphingosine (d16:1)* | Present in sake, a traditional Japanese alcoholic beverage^73^. |
| Xanthine Metabolism | caffeine | **11.01** | **3.46** | 0.02 | 0.062 | Not significant (Q>0.05) | See Discussion.  Caffeine is a psychostimulant widely available in the diet and implicated in physical performance enhancement. All other metabolites in this class are caffeine metabolites. |
|  | theophylline | **11.65** | **3.54** | 0.03 | 0.064 | Not significant (Q>0.05) |  |
|  | 7-methylxanthine | **5.24** | **2.39** | 0.03 | 0.071 | Not significant (Q>0.05) |  |
|  | 3-methylxanthine | **5.98** | **2.58** | 0.03 | 0.078 | Not significant (Q>0.05) |  |
|  | 1,3-dimethylurate | **2.67** | **1.42** | 0.004 | 0.022 | +1-methylurate, 1-methylxanthine, paraxanthine, 5-acetylamino-6-amino-3-methyluracil |  |
|  | paraxanthine | **3.12** | **1.64** | 0.016 | 0.048 | +1,3-dimethylurate, 1,7-dimethylurate, 1-methylxanthine, 1-methylurate, 5-acetylamino-6-amino-3-methyluracil |  |
|  | 1-methylurate | **3.41** | **1.77** | 1.10E-03 | 0.010 | +1,3-dimethylurate, 1-methylxanthine, paraxanthine, 1,7-dimethylurate, 5-acetylamino-6-amino-3-methyluracil |  |
|  | 1-methylxanthine | **4.22** | **2.08** | 7.00E-04 | 0.008 | + 1-methylurate, 1,3-dimethylurate, 1,7-dimethylurate, 5-acetylamino-6-amino-3-methyluracil, paraxanthine |  |
|  | 5-acetylamino-6-formylamino-3-methyluracil | **5.03** | **2.33** | 0.015 | 0.047 | + 5-acetylamino-6-amino-3-methyluracil |  |
|  | 5-acetylamino-6-amino-3-methyluracil | **5.75** | **2.52** | 0.004 | 0.021 | +5-acetylamino-6-formylamino-3-methyluracil, 1-methylurate, 1,3-dimethylurate, 1-methylxanthine, paraxanthine, 1,7-dimethylurate |  |
|  | 1,7-dimethylurate | **10.09** | **3.33** | 0.003 | 0.018 | + paraxanthine, 5-acetylamino-6-amino-3-methyluracil, 1,3-dimethylurate, 1-methylurate, 1-methylxanthine |  |
| Unknown | X-17146 | **0.08** | **-3.64** | 2.45E-10 | 1.48E-07 | Pre: detected in 100% of samples  Post: 35%  - N-palmitoyl-sphingosine (d18:1/16:0), O-sulfo-L-tyrosine | Note: X-17146 Log2FC was truncated to -1.00 for presentation in Fig1C |
|  | X-13431 | **0.72** | **-0.47** | 2.00E-04 | 0.003 |  |  |
|  | X-23678 | **0.74** | **-0.43** | 6.00E-04 | 0.007 | + X-17335 |  |
|  | X-17357 | **0.75** | **-0.42** | 4.82E-05 | 1.15E-03 | + X-11444  ML-predictor (Table S3) |  |
|  | X-25343 | **0.75** | **-0.42** | 1.40E-03 | 0.012 | + pyruvate |  |
|  | X-14904 | **0.78** | **-0.36** | 0.002 | 0.016 |  |  |
|  | X-16397 | **0.79** | **-0.34** | 0.003 | 0.019 |  |  |
|  | X-12844 | **0.80** | **-0.32** | 0.002 | 0.016 | + X-1444 |  |
|  | X-14056 | **0.81** | **-0.30** | 1.20E-03 | 0.011 | + cysteine  - 5-methyluridine (ribothymidine) |  |
|  | X-11444 | **0.81** | **-0.30** | 0.002 | 0.013 | +X-12844, X-17357 |  |
|  | X-17335 | **0.82** | **-0.29** | 0.006 | 0.028 | + X-23678 |  |
|  | X-15245 | **0.82** | **-0.29** | 0.010 | 0.038 |  |  |
|  | X-24295 | **0.84** | **-0.25** | 0.013 | 0.044 |  |  |
|  | X-24588 | **0.85** | **-0.23** | 0.004 | 0.021 | - X-24337 , X-25936 |  |
|  | X-21467 | **0.85** | **-0.23** | 0.005 | 0.024 |  |  |
|  | X-22834 | **0.86** | **-0.22** | 0.011 | 0.039 | - 5alpha-androstan-3beta,17beta-diol monosulfate (2) |  |
|  | X-23665 | **0.86** | **-0.22** | 0.014 | 0.045 | + Formiminoglutamate, X-25957 |  |
|  | X-25271 | **0.87** | **-0.20** | 6.00E-04 | 0.007 | + 2-keto-3-deoxy-gluconate |  |
|  | X-19438 | **0.87** | **-0.20** | 0.015 | 0.046 | None |  |
|  | X-25957 | **0.89** | **-0.17** | 5.00E-04 | 0.006 | + X-23665 |  |
|  | X-25936 | **0.90** | **-0.15** | 0.015 | 0.047 | - X-24588 |  |
|  | X-24699 | **1.16** | **0.21** | 1.40E-03 | 0.012 | RF predictor (Fig S5) |  |
|  | X-21829 | **1.23** | **0.30** | 0.002 | 0.016 | + X-25397, X-16245, sebacate (C10-DC), X-15220,  undecanedioate (C11-DC), azelate (C9-DC), X-24928,  X-25407, X-17438, 8-hydroxyoctanoate, X-23786,  X-25452, pelargonate (9:0). |  |
|  | X-25407 | **1.29** | **0.37** | 7.86E-05 | 0.002 | + X-16245, X-23786, X-21829, X-15220, pelargonate (9:0) |  |
|  | X-16245 | **1.31** | **0.39** | 1.63E-05 | 5.58E-04 | + X-21829, X-23786, X-25407, X-15220, X-17438, pelargonate (9:0)  RF/ML-predictor (Table S3, Fig S5) |  |
|  | X-25397 | **1.33** | **0.41** | 1.10E-03 | 0.010 | + sebacate (C10-DC), undecanedioate (C11-DC), azelate (C9-DC), X-24928  X-21829, X-25452, suberate (C8-DC), 8-hydroxyoctanoate,  X-15220, X-17438, pelargonate (9:0) |  |
|  | X-23786 | **1.44** | **0.53** | 2.17E-07 | 3.28E-05 | + X-16245, X-25407, X-21829, 2-hydroxyglutarate, pelargonate (9:0)  RF/ML-predictor (Table S3, Fig S5) |  |
|  | X-17438 | **1.44** | **0.53** | 6.91E-05 | 1.43E-03 | + X-21829, X-16245  RF-predictor (Fig S5) |  |
|  | X-18779 | **1.46** | **0.55** | 7.50E-08 | 1.51E-05 | + lactate  - taurocholenate sulfate*,  imidazole lactate  ML predictor (Fig S5) |  |
|  | X-24337 | **1.48** | **0.57** | 0.008 | 0.032 | - X-24588 |  |
|  | X-21286 | **1.51** | **0.59** | 0.013 | 0.044 | + phenylacetylglutamine, anthranilate |  |
|  | X-15220 | **1.60** | **0.68** | 2.00E-04 | 0.003 | + X-21829, X-16245, X-25407, X-25397, 8-hydroxyoctanoate, pelargonate (9:0) |  |
|  | X-24928 | **1.64** | **0.71** | 8.23E-05 | 0.002 | + X-25452, undecanedioate (C11-DC), azelate (C9-DC), X-25397  sebacate (C10-DC), suberate (C8-DC), X-21829, glycylvaline, 8-hydroxyoctanoate, pelargonate (9:0) |  |
|  | X-25452 | **1.72** | **0.78** | 2.71E-05 | 7.67E-04 | + X-24928, undecanedioate (C11-DC), azelate (C9-DC), sebacate (C10-DC), X-25397, suberate (C8-DC), 8-hydroxyoctanoate  Glycylvaline, X-21829, pelargonate (9:0) |  |
|  | X-21353 | **1.78** | **0.83** | 0.010 | 0.037 |  |  |
|  | X-18887 | **1.79** | **0.84** | 8.37E-06 | 4.08E-04 |  |  |
|  | X-25172 | **1.85** | **0.89** | 1.20E-03 | 0.011 | Pre: detected in 43% of samples  Post: 87%  + undecanoate (11:0) |  |
|  | X-21312 | **2.22** | **1.15** | 0.007 | 0.031 |  |  |
|  | X-17685 | **3.34** | **1.74** | 0.006 | 0.028 |  |  |
|  | X-12849 | **21.38** | **4.42** | 0.010 | 0.036 |  |  |

*Nominally significant (P<0.05, Q>0.05) metabolites are tabulated if members of pathways presenting with significant enrichment (see Results)

† Cells are red for metabolites that increased and green for metabolites that decreased from Pre- to Post-season. Box plots of all metabolites that significantly changed after a season of football based on paired t-tests are available upon request.

# Table S3. mPLSDA: Top 20 discriminative metabolites

| **Sub Pathway** | **Biochemical** | **Rank Product (RP)** |
| --- | --- | --- |
| Fatty Acid, Dicarboxylate | 2-hydroxyglutarate | 2.43* |
| Unknown | X - 18779 | 4.21* |
| Unknown | X - 23786 | 6.58* |
| Unknown | X - 17357 | 11.89* |
| Fatty Acid, Dicarboxylate | sebacate (C10-DC) | 15.48* |
| Phosphatidylethanolamine (PE) | 1-stearoyl-2-arachidonoyl-GPE (18:0/20:4) | 15.75* |
| Fatty Acid, Monohydroxy | 8-hydroxyoctanoate | 15.92* |
| Fructose, Mannose and Galactose Metabolism | fructose | 17.69* |
| Corticosteroids | cortisol | 17.75* |
| Medium Chain Fatty Acid | heptanoate (7:0) | 21.55* |
| Fructose, Mannose and Galactose Metabolism | mannose | 24.52* |
| Unknown | X - 16245 | 27.21* |
| Androgenic Steroids | androstenediol (3alpha, 17alpha) monosulfate (2) | 28.47 |
| Fatty Acid, Dicarboxylate | suberate (C8-DC) | 31.57 |
| Medium Chain Fatty Acid | pelargonate (9:0) | 40.82 |
| Urea cycle; Arginine and Proline Metabolism | pro-hydroxy-pro | 46.40 |
| Unknown | X - 25407 | 47.70 |
| Medium Chain Fatty Acid | caproate (6:0) | 49.34 |
| Unknown | X - 23678 | 49.77 |

*Significantly discriminative: RP values of the mPLSDA model were compared with the RP values obtained from 1000 permutations and those with a P value <0.05 were considered significantly discriminative.

# Table S4. HAE-metabolite regression results

Key

mPLSDA analysis

RF analysis

***appears in enriched pathway***

Results are listed for regressions with P<0.05. “Pre 🡪 Post” indicates the directionality of metabolite change across-season. Class and sub-class refer to the characterization of each metabolite. HAE = head acceleration event metric, Std. $\beta$ = normalized beta coefficient, r^2^ = r-squared, # outliers = number of Cook’s distance outliers.

# Figure S1.

A. B.


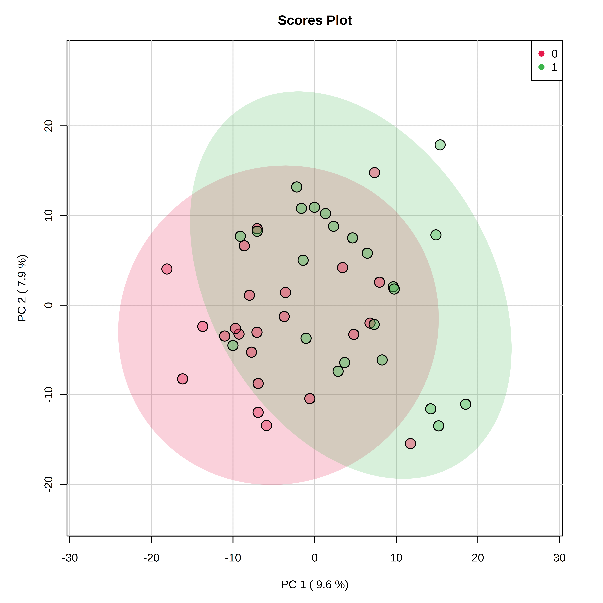

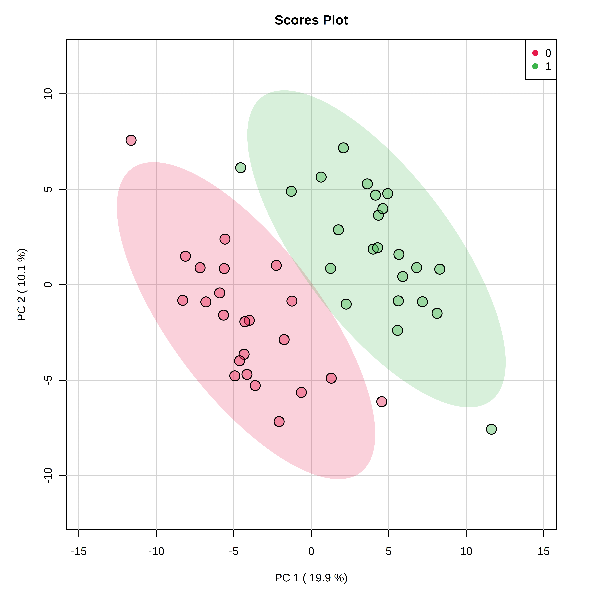


**Results from PCA (A) and multilevel PCA (B) analysis.** Score plot of the first and second PC. Pre- and Post-season samples are colored red and green, accordingly.

# Figure S2.

A


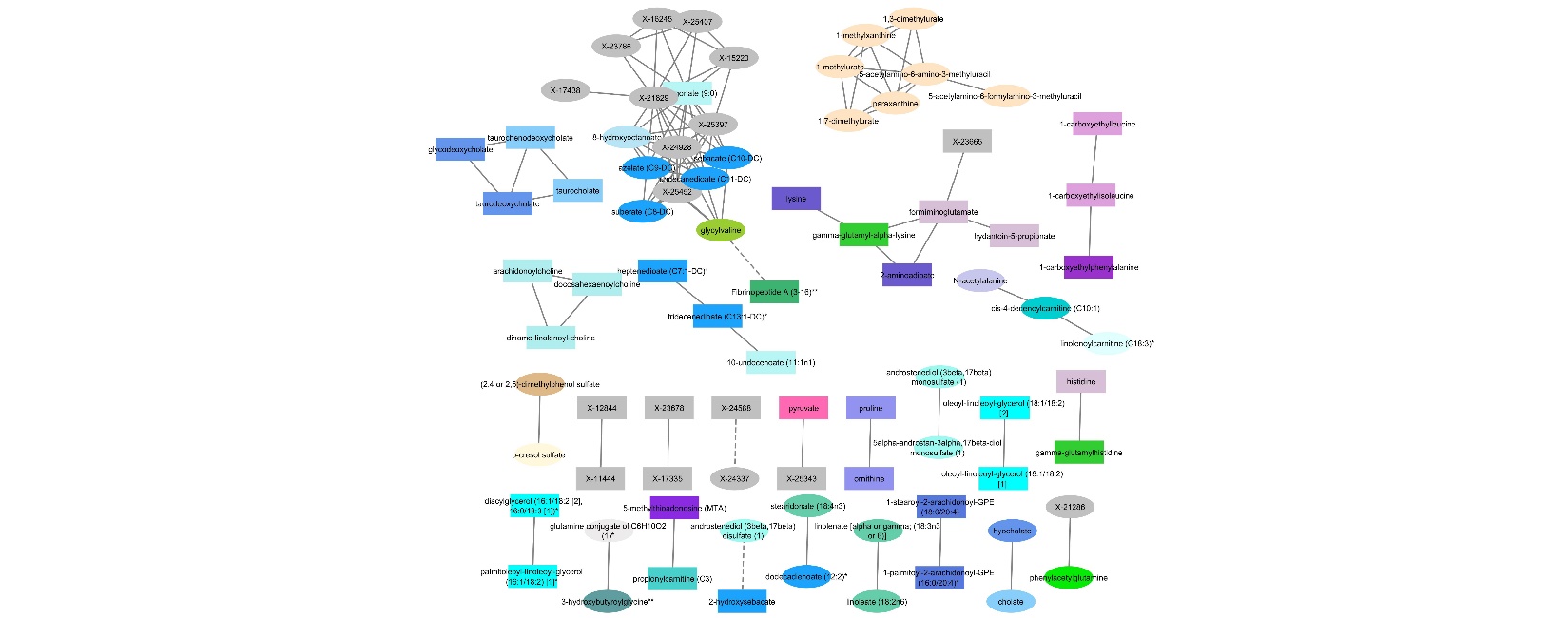


B


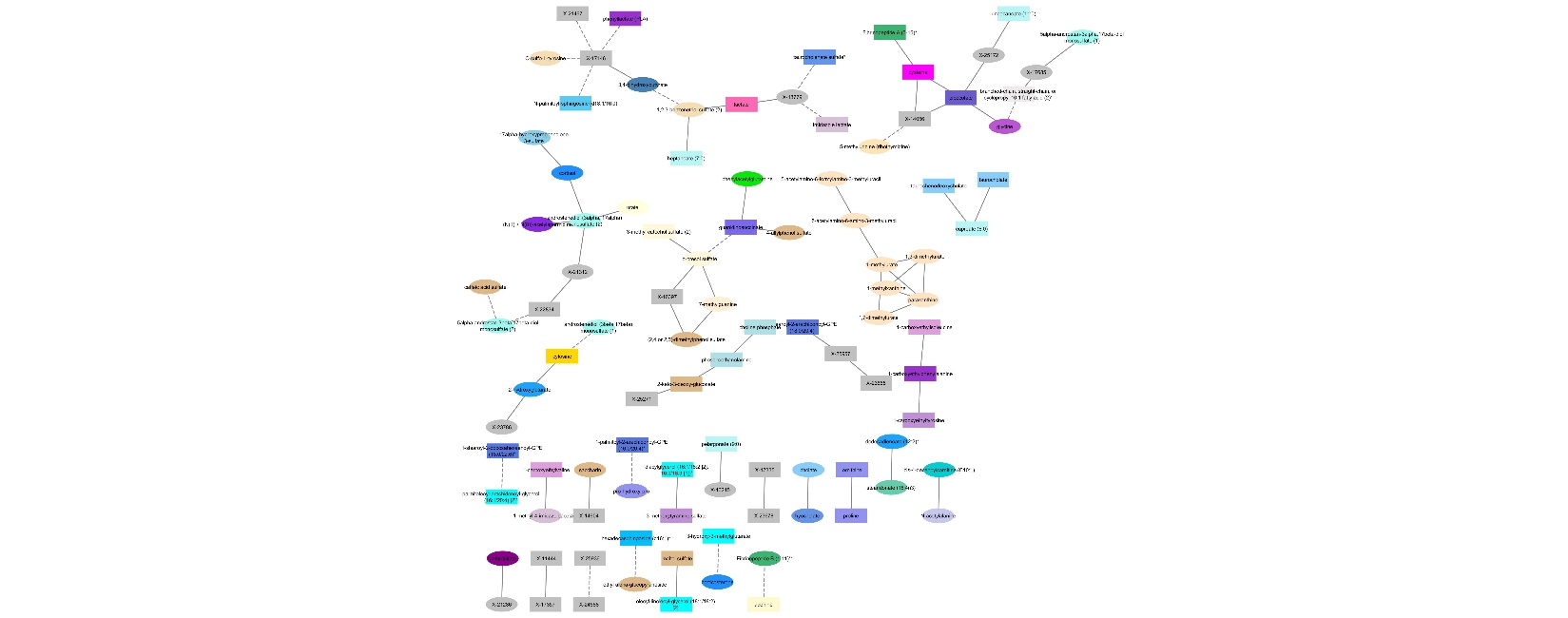


A. Pearson correlations of changes in metabolite levels after a season of football. Edges correspond to r and are shown if |r|> 0.80. B. Significant (P<0.05) partial correlations (r_par_) of changes in metabolite levels after a season of football (max |r_par_|=0.10). Node colors correspond to subpathways; see Fig 1A of main paper. Distances between nodes have no meaning.

# Figure S3.


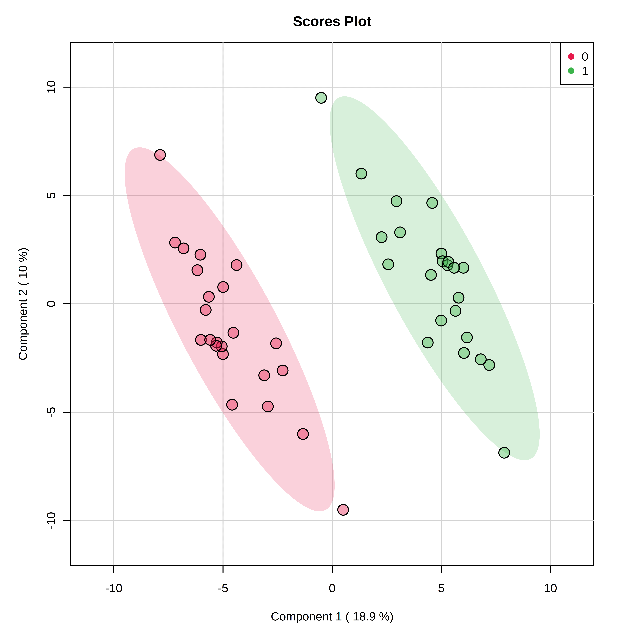

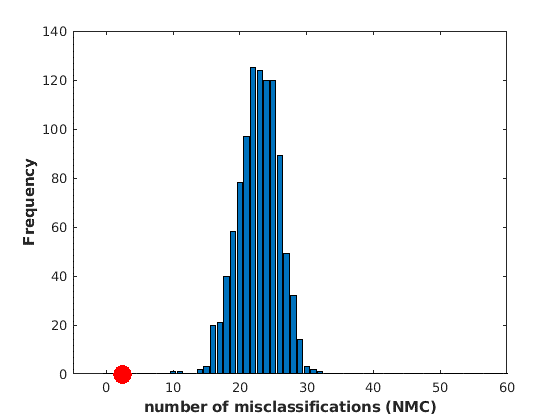


**mPLSDA score plot and corresponding permutation-testing of mPLSDA model.** Pre- and Post-season samples are colored red and green, accordingly. We caution that mPLSDA score plots present overoptimistic representation of classification but it may reveal structure within a class ^74^. Comparison of the classification error (estimated in NMC) of the mPLSDA model (red) against the permutations (blue). The prediction error is much smaller as compared to the permutations and thus the statistical significance of the model is confirmed (P<0.05).

# Figure S4.


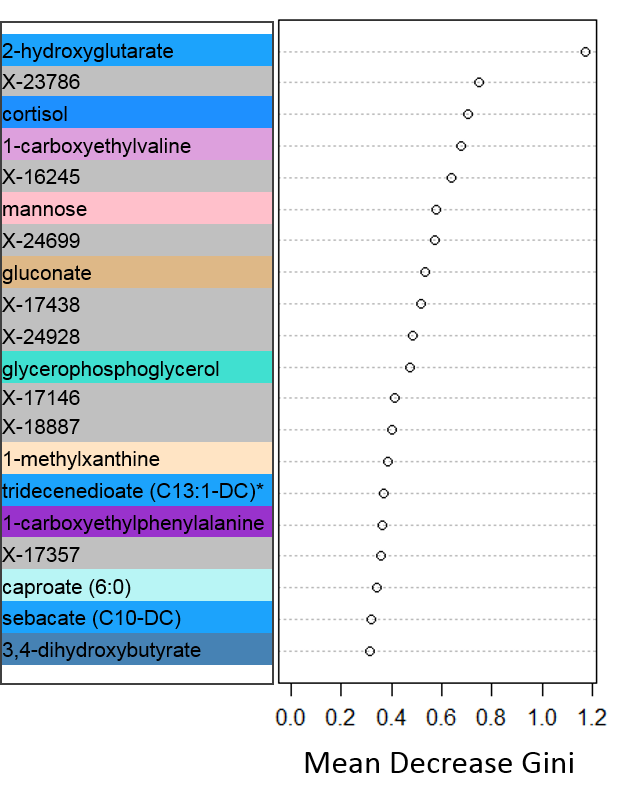


**Results from multilevel RF analysis.** Shown are the most representative metabolites that differentiate Pre- and Post-season samples. The Gini “score”, the scaled sum of votes derived from the trained trees for out-of-bag samples, was used as an indicator of the importance of a variable or how much it contributes to predictive accuracy. The larger the Gini score is (ranges from 1 to 100), the more important a variable. Cell colors correspond to supathways; see Fig 1A of main paper. CMV mean error rate was 4%, with the lowest error rates observed with predictions based on 4 to 9 metabolites (1%).

# SUPPLEMENTAL REFERENCES

1 Lee, T. *et al.* Head acceleration event metrics in youth contact sports more dependent on sport than level of play. *Proceedings of the Institution of Mechanical Engineers, Part H: Journal of engineering in medicine*, 0954411920970812 (2020).

2 Bari, S. *et al.* Dependence on subconcussive impacts of brain metabolism in collision sport athletes: an MR spectroscopic study. *Brain imaging and behavior* **13**, 735-749 (2019).

3 McCuen, E. *et al.* Collegiate women's soccer players suffer greater cumulative head impacts than their high school counterparts. *Journal of biomechanics* **48**, 3720-3723 (2015).

4 Dehaven, C. D., Evans, A. M., Dai, H. & Lawton, K. A. Organization of GC/MS and LC/MS metabolomics data into chemical libraries. *Journal of cheminformatics* **2**, 9, doi:10.1186/1758-2946-2-9 (2010).

5 Bijlsma, S. *et al.* Large-scale human metabolomics studies: a strategy for data (pre-) processing and validation. *Anal Chem* **78**, 567-574, doi:10.1021/ac051495j (2006).

6 Schafer, J. & Strimmer, K. A shrinkage approach to large-scale covariance matrix estimation and implications for functional genomics. *Stat. Appl. Genet. Molec. Biol* **4**, 1175-1189 (2005).

7 Wishart, D. S. *et al.* HMDB 3.0--The Human Metabolome Database in 2013. *Nucleic Acids Res* **41**, D801-807, doi:10.1093/nar/gks1065gks1065 [pii] (2013).

8 Kim, S. *et al.* PubChem 2019 update: improved access to chemical data. *Nucleic Acids Research* **47**, D1102-D1109, doi:10.1093/nar/gky1033 (2018).

9 Nieman, D. C., Shanely, R. A., Gillitt, N. D., Pappan, K. L. & Lila, M. A. Serum metabolic signatures induced by a three-day intensified exercise period persist after 14 h of recovery in runners. *Journal of proteome research* **12**, 4577-4584 (2013).

10 Karl, J. P. *et al.* Military training elicits marked increases in plasma metabolomic signatures of energy metabolism, lipolysis, fatty acid oxidation, and ketogenesis. *Physiol Rep* **5**, doi:10.14814/phy2.13407 (2017).

11 Kreider, R. B. *et al.* International Society of Sports Nutrition position stand: safety and efficacy of creatine supplementation in exercise, sport, and medicine. *Journal of the International Society of Sports Nutrition* **14**, 18, doi:10.1186/s12970-017-0173-z (2017).

12 Virgili, F. *et al.* Relationship between fat-free mass and urinary excretion of creatinine and 3-methylhistidine in adult humans. *Journal of applied physiology* **76**, 1946-1950, doi:10.1152/jappl.1994.76.5.1946 (1994).

13 Cao, B., Liu, S., Yang, L. & Chi, A. Changes of Differential Urinary Metabolites after High-Intensive Training in Teenage Football Players. *BioMed research international* **2020** (2020).

14 Barton, W. *et al.* The microbiome of professional athletes differs from that of more sedentary subjects in composition and particularly at the functional metabolic level. *Gut* **67**, 625-633, doi:10.1136/gutjnl-2016-313627 (2018).

15 Al-Khelaifi, F. *et al.* Metabolic profiling of elite athletes with different cardiovascular demand. *Scandinavian Journal of Medicine & Science in Sports* **29**, 933-943, doi:10.1111/sms.13425 (2019).

16 Daley, M. *et al.* Metabolomics profiling of concussion in adolescent male hockey players: a novel diagnostic method. *Metabolomics* **12**, 185 (2016).

17 Mrakic-Sposta, S. *et al.* Acute Effects of Triathlon Race on Oxidative Stress Biomarkers. *Oxid Med Cell Longev* **2020**, 3062807, doi:10.1155/2020/3062807 (2020).

18 Jang, H. J. *et al.* Metabolic Profiling of Eccentric Exercise-Induced Muscle Damage in Human Urine. *Toxicological research* **34**, 199-210, doi:10.5487/tr.2018.34.3.199 (2018).

19 Hu, C. *et al.* Muscle-liver substrate fluxes in exercising humans and potential effects on hepatic metabolism. *The Journal of Clinical Endocrinology & Metabolism* **105**, 1196-1209 (2020).

20 Stander, Z. *et al.* The altered human serum metabolome induced by a marathon. *Metabolomics* **14**, 150, doi:10.1007/s11306-018-1447-4 (2018).

21 Schranner, D., Kastenmüller, G., Schönfelder, M., Römisch-Margl, W. & Wackerhage, H. Metabolite Concentration Changes in Humans After a Bout of Exercise: a Systematic Review of Exercise Metabolomics Studies. *Sports Medicine - Open* **6**, 11, doi:10.1186/s40798-020-0238-4 (2020).

22 Dash, P. K. *et al.* Traumatic Brain Injury Alters Methionine Metabolism: Implications for Pathophysiology. *Frontiers in Systems Neuroscience* **10**, doi:10.3389/fnsys.2016.00036 (2016).

23 Al-Khelaifi, F. *et al.* A pilot study comparing the metabolic profiles of elite-level athletes from different sporting disciplines. *Sports medicine-open* **4**, 2 (2018).

24 Lewis, G. D. *et al.* Metabolic signatures of exercise in human plasma. *Science translational medicine* **2**, 33ra37, doi:10.1126/scitranslmed.3001006 (2010).

25 Brennan, A. M. *et al.* Plasma metabolite profiles in response to chronic exercise. *Medicine and science in sports and exercise* **50**, 1480 (2018).

26 Berton, R. *et al.* Metabolic time-course response after resistance exercise: A metabolomics approach. *Journal of sports sciences* **35**, 1211-1218, doi:10.1080/02640414.2016.1218035 (2017).

27 Chorell, E., Svensson, M. B., Moritz, T. & Antti, H. Physical fitness level is reflected by alterations in the human plasma metabolome. *Molecular bioSystems* **8**, 1187-1196 (2012).

28 Lustgarten, M. S. *et al.* Serum predictors of percent lean mass in young adults. *Journal of strength and conditioning research* **30**, 2194-2201 (2016).

29 San-Millán, I. *et al.* Metabolomics of Endurance Capacity in World Tour Professional Cyclists. *Frontiers in Physiology* **11**, doi:10.3389/fphys.2020.00578 (2020).

30 Rogers, P. J. *et al.* Catecholamine metabolic pathways and exercise training. Plasma and urine catecholamines, metabolic enzymes, and chromogranin-A. *Circulation* **84**, 2346-2356 (1991).

31 Sothmann, M. S. *et al.* Plasma free and sulfoconjugated catecholamines during sustained exercise. *Journal of applied physiology* **68**, 452-456, doi:10.1152/jappl.1990.68.2.452 (1990).

32 Daskalaki, E. *et al.* A study of the effects of exercise on the urinary metabolome using normalisation to individual metabolic output. *Metabolites* **5**, 119-139 (2015).

33 Jeter, C. B., Hergenroeder, G. W., Ward III, N. H., Moore, A. N. & Dash, P. K. Human traumatic brain injury alters circulating L-arginine and its metabolite levels: possible link to cerebral blood flow, extracellular matrix remodeling, and energy status. *Journal of neurotrauma* **29**, 119-127 (2012).

34 Orešič, M. *et al.* Human Serum Metabolites Associate With Severity and Patient Outcomes in Traumatic Brain Injury. *EBioMedicine* **12**, 118-126, doi:<https://doi.org/10.1016/j.ebiom.2016.07.015> (2016).

35 Sugino, T., Shirai, T., Kajimoto, Y. & Kajimoto, O. L-ornithine supplementation attenuates physical fatigue in healthy volunteers by modulating lipid and amino acid metabolism. *Nutrition research (New York, N.Y.)* **28**, 738-743, doi:10.1016/j.nutres.2008.08.008 (2008).

36 Demura, S., Yamada, T., Yamaji, S., Komatsu, M. & Morishita, K. The effect of L-ornithine hydrochloride ingestion on performance during incremental exhaustive ergometer bicycle exercise and ammonia metabolism during and after exercise. *Eur J Clin Nutr* **64**, 1166-1171, doi:10.1038/ejcn.2010.149 (2010).

37 Koay, Y. C. *et al.* Effect of chronic exercise in healthy young male adults: a metabolomic analysis. *Cardiovascular research*, doi:10.1093/cvr/cvaa051 (2020).

38 Velenosi, T. J. *et al.* Untargeted metabolomics reveals N, N, N-trimethyl-L-alanyl-L-proline betaine (TMAP) as a novel biomarker of kidney function. *Scientific reports* **9**, 1-13 (2019).

39 Shi, R. *et al.* Runners' metabolomic changes following marathon. *Nutrition & metabolism* **17**, 19, doi:10.1186/s12986-020-00436-0 (2020).

40 Stander, Z. *et al.* The unaided recovery of marathon-induced serum metabolome alterations. *Scientific reports* **10**, 11060, doi:10.1038/s41598-020-67884-9 (2020).

41 Philp, A., Macdonald, A. L. & Watt, P. W. Lactate – a signal coordinating cell and systemic function. *Journal of Experimental Biology* **208**, 4561-4575, doi:10.1242/jeb.01961 (2005).

42 San-Millán, I. in *High-Throughput Metabolomics* 431-446 (Springer, 2019).

43 Tanaka, Y., Wood, L. A. & Cooney, R. V. Enhancement of intracellular gamma-tocopherol levels in cytokine-stimulated C3H 10T1/2 fibroblasts: relation to NO synthesis, isoprostane formation, and tocopherol oxidation. *BMC chemical biology* **7**, 2, doi:10.1186/1472-6769-7-2 (2007).

44 Cooney, R. V., Franke, A. A., Wilkens, L. R., Gill, J. & Kolonel, L. N. Elevated plasma gamma-tocopherol and decreased alpha-tocopherol in men are associated with inflammatory markers and decreased plasma 25-OH vitamin D. *Nutrition and cancer* **60 Suppl 1**, 21-29, doi:10.1080/01635580802404162 (2008).

45 Bredle, D., Stager, J., Brechue, W. & Farber, M. Phosphate supplementation, cardiovascular function, and exercise performance in humans. *Journal of applied physiology* **65**, 1821-1826 (1988).

46 Dale, G., Fleetwood, J., Weddell, A., Ellis, R. & Sainsbury, J. Fitness, unfitness, and phosphate. *British medical journal (Clinical research ed.)* **294**, 939 (1987).

47 Lusczek, E. R. *et al.* Plasma metabolomics pilot study suggests age and sex-based differences in the metabolic response to traumatic injury. *Injury* **49**, 2178-2185 (2018).

48 Kraemer, W. J. & Ratamess, N. A. Hormonal responses and adaptations to resistance exercise and training. *Sports medicine* **35**, 339-361 (2005).

49 Tanriverdi, F. & Kelestimur, F. Pituitary dysfunction following traumatic brain injury: clinical perspectives. *Neuropsychiatric disease and treatment* **11**, 1835 (2015).

50 Morena, M., Patel, S., Bains, J. S. & Hill, M. N. Neurobiological interactions between stress and the endocannabinoid system. *Neuropsychopharmacology* **41**, 80-102 (2016).

51 Cristino, L., Bisogno, T. & Di Marzo, V. Cannabinoids and the expanded endocannabinoid system in neurological disorders. *Nature Reviews Neurology*, 1-21 (2019).

52 Jeter, C. B., Hergenroeder, G. W., Ward III, N. H., Moore, A. N. & Dash, P. K. Human mild traumatic brain injury decreases circulating branched-chain amino acids and their metabolite levels. *Journal of neurotrauma* **30**, 671-679 (2013).

53 Mingrone, G. & Castagneto, M. Medium-chain, even-numbered dicarboxylic acids as novel energy substrates: an update. *Nutrition reviews* **64**, 449-456 (2006).

54 Jin, S. J. & Tserng, K. Y. Metabolic origins of urinary unsaturated dicarboxylic acids. *Biochemistry* **29**, 8540-8547 (1990).

55 Nieman, D. C. *et al.* Metabolomics approach to assessing plasma 13-and 9-hydroxy-octadecadienoic acid and linoleic acid metabolite responses to 75-km cycling. *American Journal of Physiology-Regulatory, Integrative and Comparative Physiology* **307**, R68-R74 (2014).

56 Pilitsis, J. G. *et al.* Free fatty acids in cerebrospinal fluids from patients with traumatic brain injury. *Neuroscience letters* **349**, 136-138 (2003).

57 Zheng, F. *et al.* Plasma metabolomics profiles in rats with acute traumatic brain injury. *PloS one* **12**, e0182025 (2017).

58 Desai, A. *et al.* Reduced acute neuroinflammation and improved functional recovery after traumatic brain injury by α-linolenic acid supplementation in mice. *Journal of neuroinflammation* **13**, 253 (2016).

59 Kumar, P. R. *et al.* Omega-3 Fatty acids could alleviate the risks of traumatic brain injury–a mini review. *Journal of traditional and complementary medicine* **4**, 89-92 (2014).

60 Fiandaca, M. S. *et al.* Plasma metabolomic biomarkers accurately classify acute mild traumatic brain injury from controls. *PloS one* **13**, e0195318 (2018).

61 Morville, T. *et al.* Divergent effects of resistance and endurance exercise on plasma bile acids, FGF19, and FGF21 in humans. *JCI insight* **3** (2018).

62 Gronbeck, K. R. *et al.* Application of Tauroursodeoxycholic Acid for Treatment of Neurological and Non-neurological Diseases: Is There a Potential for Treating Traumatic Brain Injury? *Neurocritical care* **25**, 153-166, doi:10.1007/s12028-015-0225-7 (2016).

63 Quintas, G. *et al.* Urine metabolomic analysis for monitoring internal load in professional football players. *Metabolomics* **16**, 1-11 (2020).

64 Saunders, B. *et al.* β-alanine supplementation to improve exercise capacity and performance: a systematic review and meta-analysis. *British Journal of Sports Medicine* **51**, 658-669 (2017).

65 Barrios, C. *et al.* Gut-Microbiota-Metabolite Axis in Early Renal Function Decline. *PLoS One* **10**, e0134311, doi:10.1371/journal.pone.0134311 (2015).

66 Refaai, M. A., Riley, P., Mardovina, T. & Bell, P. D. The clinical significance of fibrin monomers. *Thrombosis and haemostasis* **118**, 1856-1866 (2018).

67 Zhu, Y., Wang, P., Sha, W. & Sang, S. Urinary Biomarkers of Whole Grain Wheat Intake Identified by Non-targeted and Targeted Metabolomics Approaches. *Scientific reports* **6**, 36278, doi:10.1038/srep36278 (2016).

68 Al-Khelaifi, F. *et al.* Metabolomics profiling of xenobiotics in elite athletes: relevance to supplement consumption. *Journal of the International Society of Sports Nutrition* **15**, 48 (2018).

69 Niewczas, M. A. *et al.* Circulating Modified Metabolites and a Risk of ESRD in Patients With Type 1 Diabetes and Chronic Kidney Disease. *Diabetes Care* **40**, 383-390, doi:10.2337/dc16-0173 (2017).

70 Velenosi, T. J. *et al.* Untargeted metabolomics reveals N, N, N-trimethyl-L-alanyl-L-proline betaine (TMAP) as a novel biomarker of kidney function. *Scientific reports* **9**, 6831, doi:10.1038/s41598-019-42992-3 (2019).

71 Rothwell, J. A. *et al.* Phenol-Explorer 3.0: a major update of the Phenol-Explorer database to incorporate data on the effects of food processing on polyphenol content. *Database* **2013** (2013).

72 Bieniek, G. Urinary excretion of phenols as an indicator of occupational exposure in the coke-plant industry. *International archives of occupational and environmental health* **70**, 334-340, doi:10.1007/s004200050227 (1997).

73 Imanari, T. & Tamura, Z. The identification of α-ethyl glucoside and sugar-alcohols in sake. *Agricultural and Biological Chemistry* **35**, 321-324 (1971).

74 Westerhuis, J. A. *et al.* Assessment of PLSDA cross validation. *Metabolomics* **4**, 81-89 (2008).
